# Advanced physical modeling approaches for high-precision TCAD simulation of GaN HEMT power devices: a review

**DOI:** 10.1186/s11671-026-04571-0

**Published:** 2026-04-30

**Authors:** Haocheng Zhao, Amirul Firdaus, Muhammad Farizuan, Weng-Hooi Tan, Hiroshi Kawarada, Shaili Falina, Mohd Syamsul

**Affiliations:** 1 Institute of Nano Optoelectronics Research and Technology (INOR), Universiti Sains Malaysia, Sains@USM, 11900 Bayan Lepas, Pulau Pinang Malaysia; 2 Collaborative Microelectronic Design Excellence Center (CEDEC), Universiti Sains Malaysia, Sains@USM, 11900 Bayan Lepas, Pulau Pinang Malaysia; 3 Faculty of Science and Engineering, Waseda University, Shinjuku, Tokyo 169-8555 Japan

**Keywords:** GaN, HEMT, TCAD, Simulation, Physical model, Power device

## Abstract

High-electron-mobility transistors (HEMTs) based on wide bandgap (WBG) materials like gallium nitride (GaN) are vital for next-generation power electronics and high-frequency applications, offering high breakdown voltage, electron mobility, and power density. The global shift toward electrification and sustainability is driving demand for GaN and silicon carbide (SiC) power devices. However, challenges such as current collapse and increased channel resistance under high-power conditions hinder performance. To address these limitations, numerous solutions have been explored, with simulation emerging as an indispensable starting point. Technology Computer-Aided Design (TCAD) simulations play a critical role by enabling accurate modeling, performance optimization, and reduced experimental effort. This paper reviews key and advanced physical models in TCAD simulations of GaN HEMTs, covering mechanisms such as carrier transport, thermal effects, and impact ionization. Mobility models—FLDMOB, Albrecht, Gansat, Yamaguchi, Brooks-Herring, and Conwell-Weisskopf—are analyzed for capturing velocity saturation and nonlocal transport. Recombination models like Shockley-Read-Hall and Auger are discussed in relation to carrier lifetime, while impact ionization models, including van-Overstraeten-de-Man, Selberherr, and Okuto-Crowell, are evaluated for breakdown prediction. Emphasis is placed on choosing models suited to specific structures and conditions to ensure simulation accuracy. Advanced modeling enhances TCAD’s predictive power, supporting innovation in GaN-based power electronics.

**Supplementary Information:**

The online version contains supplementary material available at 10.1186/s11671-026-04571-0.

## Introduction

**Fig. 1:**
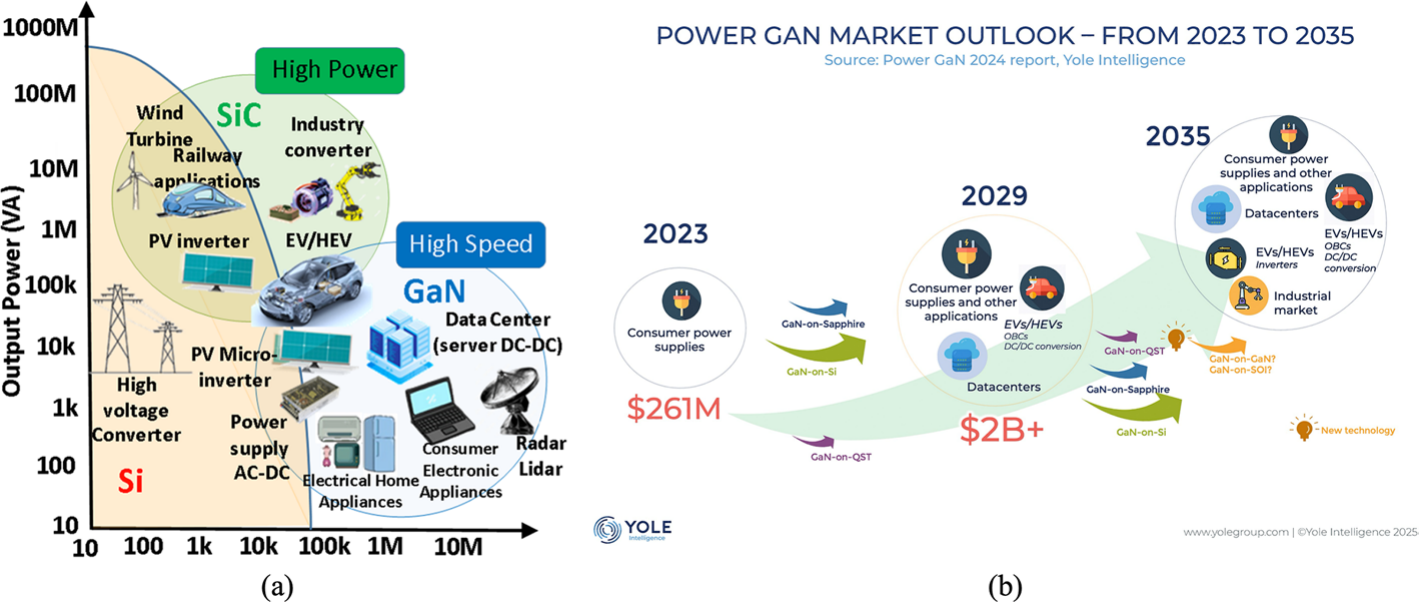
**a** Application scope based on WBG materials. Reprinted with permission from ref. [94]. Copyright 2023 MDPI.** b** GaN power electronic devices in long-term evolution for the Chinese market (LEROY, [101]. Source Power GaN report, Yole intelligence 2023

Semiconductor power devices are essential for modern electronics, enabling efficient control and conversion of current and voltage in applications such as switching power supplies, converters, inverters, and motor drives [70]. However, due to the inherent physical limitations of silicon, most of the aforementioned Si-based power devices face challenges in meeting the demands of next-generation power electronics. As a result, research efforts have increasingly focused on exploring alternative materials to replace silicon in power device applications [139]. The introduction of WBG materials, such as SiC and GaN, has marked a significant breakthrough in the field of power semiconductor devices [121]. These materials offer several advantages over silicon, including higher dielectric strength, greater saturation drift velocity, and enhanced performance in harsh environments [93]. The distinct properties of GaN and SiC make them suitable for different application domains—GaN excels in high-frequency power electronics, while SiC is better suited for high-voltage power applications, such as electric vehicles and grid infrastructure. Figure 1(a) illustrates the typical application ranges of GaN and SiC in power electronics [128].

The GaN semiconductor device market is expanding rapidly, driven by its high efficiency and strong performance in high-frequency, high-power applications. In 2024, sales are projected to grow 19% to $627 billion and reach $697 billion in 2025, putting the industry on track toward $1 trillion by 2030 and potentially $2 trillion by 2040 [94]. A major contributor is China’s GaN power device market, expected to reach $2 billion by 2029 with applications in consumer supplies, electric vehicles, and data centers, as shown in Fig. 1(b) (LEROY, [101]. At the device level, HEMTs are emerging as superior alternatives to Si MOSFETs, offering high breakdown voltage, electron mobility (~$$ 2000{\mathrm{cm}}^{{\mathrm{2}}} {\mathrm{/V}} \cdot {\mathrm{s}} $$), and carrier density (~$$ 10^{{13}} {\mathrm{cm}}^{{ - {\mathrm{2}}}} $$) [137, 184, 103]. These properties, enabled by the 2D electron gas at the AlGaN/GaN heterointerface, allow outstanding high-frequency and high-power performance [124]. Gate lengths near 100 nm have achieved unity-gain and maximum oscillation frequencies above 100 GHz, making GaN HEMTs strong candidates for 5G base stations [54, 71, 129, 162, 172]. Their robustness under high power, temperature, and frequency further supports use in telecommunications, satellites, and defense systems [171]. In addition to RF and communication applications, GaN HEMTs are increasingly adopted in commercial power electronics, such as consumer fast chargers and adapters, automotive powertrains and onboard chargers for electric vehicles, data center power supplies, and renewable energy converters for solar and wind systems [9]. They are also used in radar systems, RF front-ends, and electronic warfare platforms, as well as in aerospace and avionics for efficient power management under extreme conditions [102]. However, as GaN scales into the submicron regime, accurate prediction of device behavior becomes challenging due to nonlinear and multiphysical effects. Technology Computer-Aided Design (TCAD) provides an efficient solution, enabling physics-based simulation of transport, breakdown, and thermal effects, reducing costly experiments and accelerating development [155, 156]. The International Technology Roadmap for Semiconductors (ITRS) has estimated TCAD adoption can reduce development costs by 40% [112], underscoring its importance in advancing GaN HEMT research and innovation.

TCAD simulation enables detailed modeling of HEMT power devices with various materials and structures, where the choice of physical models plays a decisive role in determining accuracy. The impact of material selection is especially evident in heterojunctions such as AlGaAs/GaAs, AlGaN/GaN, and AlN/β-Ga_2_O_3_, each of which exhibits distinct mechanisms for two-dimensional electron gas (2DEG) formation. In AlGaAs/GaAs HEMTs, modulation doping dominates, whereas in III-nitrides, quantum confinement and strong polarization effects are critical. Similarly, p-GaN gate HEMTs require explicit modeling of p-type doping, since the primary carrier type shifts to holes, and neglecting this can lead to misleading results. Furthermore, breakdown analysis demands the use of proper impact ionization models to capture avalanche effects, without which the simulated breakdown voltage may deviate significantly from experimental data. Importantly, the selection of mobility, recombination, and transport models can lead to large variations in simulated device behavior, even under the same structural parameters. For example, using a simple constant mobility model may miss electron velocity overshoot effects, whereas advanced field-dependent mobility models more accurately capture high-field transport. Likewise, the choice between different impact ionization models can produce different breakdown characteristics, and varying recombination models can alter predictions of leakage current and reliability. These differences highlight that TCAD simulations are not universal but highly dependent on the underlying physical models. Therefore, careful model selection—tailored to both the material system and the specific operating conditions—is essential for producing reliable and predictive simulations.

Although GaN HEMTs have been widely studied, most papers focus on the results analysis, with limited attention to the specific computational models used in TCAD. This review addresses that gap by linking the fundamental semiconductor equations to their application in GaN HEMT simulations. The focus is made on a wide range of advanced models, including mobility, recombination, and impact ionization models, with emphasis on their impact on their respective simulation accuracies. This review provides comparative insights into model selection, highlighting how different physical models can lead to large variations in predicted device behaviors. Through this integrated approach, the review offers a unique contribution by bridging conventional device physics with advanced TCAD modeling, thereby serving as a practical guide for improving the predictive reliability of GaN HEMT simulations.

This paper reviews the fundamental computational models used in HEMT simulations and examines how advanced physical models can enhance the accuracy of device simulations. It is outlined in 5 sections. Section 1 introduces the background of GaN HEMTs. Section 2 focuses on the AlGaN/GaN HEMT structure, exploring the mechanism of 2DEG formation and analyzing key material parameters. Section 3 delves into the core semiconductor equations essential for TCAD simulations of AlGaN/GaN HEMTs, including Poisson’s equation, the carrier continuity equation, the drift-diffusion transport equation, and the lattice heat flow equation, which collectively model HEMT performance. Building on this foundation, Sect. 4 examines mobility models, transition models, and impact ionization models specific to HEMT structures. It further discusses the necessity of replacing standard models with advanced, parameter-dependent models—such as those accounting for doping concentration and temperature—to improve simulation accuracy. The adoption of these advanced models ultimately enhances the reliability of HEMT performance predictions, leading to more precise and efficient device simulations. Section 5 concludes the overviews.

## HEMT power devices and key parameters

HEMT has been studied for decades. In 1979, Takashi Mimura first came up with the idea of using a field effect to control electrons at the interface of a single heterojunction [122]. In 1980, the first HEMT was realized by him and his team at Fujitsu Laboratories in Japan [17, 123]. The structural design of the new HEMT is derived from the heterojunction structure between GaAs (gallium arsenide) and AlGaAs (aluminum gallium arsenide) added to the FET (field effect transistor). The heterojunction structure provides the device with higher electron mobility compared to conventional FETs. As research progresses, GaN-based HEMTs have received widespread attention. Compared with similar materials such as GaAs and Silicon (Si), GaN has more advantages and can be used in more application fields. GaN offers significantly higher power density and breakdown field compared to materials like Si and GaAs [183]. Additionally, GaN’s superior thermal conductivity enhances its performance, particularly in high-power and high-temperature applications [105]. Higher breakdown voltage and the ability to operate at high temperatures make GaN-based HEMTs ideal for high-power applications, including automotive, military, aerospace and telecommunications [130, 147, 180].

### Overview of HEMTs

**Fig. 2:**
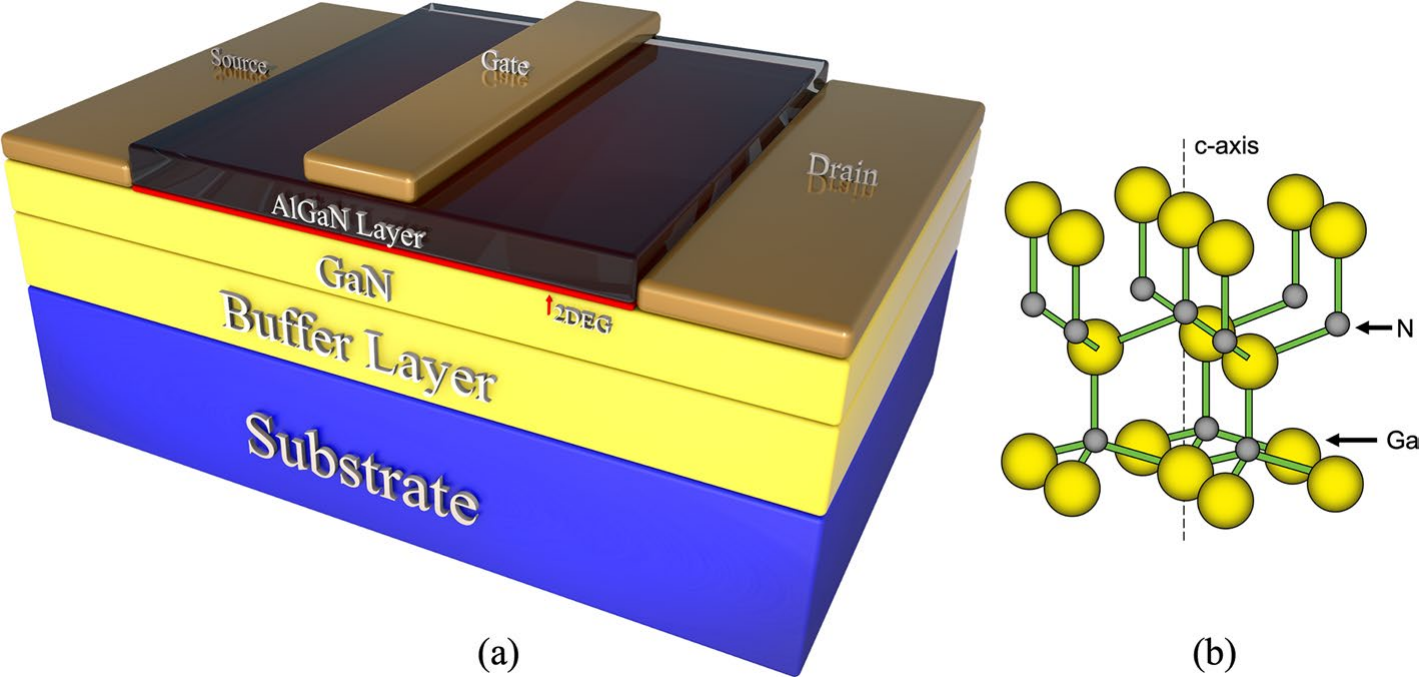
**a** the typical lateral AlGaN/GaN HEMT structure and** b** the wurtzite crystal structure of GaN. Reproduced from ref. [160] with permission from arXiv, copyright 2022

A HEMT is a type of semiconductor device that incorporates a heterojunction formed by combining materials with different band gaps. These materials typically have a crystalline structure, with some (such as GaN, ZnO, and AlN) exhibiting the wurtzite crystal structure characterized by a hexagonal lattice. The wurtzite structure is among the most prevalent in modern semiconductors, and its intrinsic spontaneous polarization is a key material property [39]. Figure 2(a) illustrates a typical lateral depletion-mode (D-mode) AlGaN/GaN HEMT configuration, which comprises multiple epitaxial layers. These include both wide and narrow bandgap materials, forming the AlGaN/GaN heterojunction. At the interface between AlGaN and GaN, a two-dimensional electron gas (2DEG) is generated, enabling electron transport without the need for intentional doping [120]. The creation of the 2DEG is driven by a combination of spontaneous polarization and piezoelectric effects [6, 7].

Epitaxially grown III-V semiconductor materials with a wurtzite structure, such as GaN (Fig. 2(b) [160]) and AlN, exhibit strong polarization effects, which are either absent or significantly reduced in zincblende III-V materials such as GaAs. The polarization phenomena is critical when modeling nitride-based heterostructures and multilayered devices, as they fundamentally alter energy band profiles, carrier distributions, and ultimately, overall device performance [16, 189, 154]. The total macroscopic polarization can be mathematically described by the following equation:

1 $$ P_{total} = P_{sp} + P_{pz} $$

where ${\mathrm{P}}_{{{\mathrm{sp}}}}$ is spontaneous polarization. ${\mathrm{P}}_{{{\mathrm{pz}}}}$ is piezoelectric polarization. Spontaneous polarization is an intrinsic property of the material, meaning it exists without any external influence. Piezoelectric polarization occurs when the material is subjected to mechanical strain, which typically occurs due to lattice mismatch between the AlGaN and GaN layers.

For III-V materials with three types of elements, such as AlGaN, where the composition includes Al, Ga and N in nitride form. Calculations of its properties, including polarization equations, generally follow similar interpolation rules as for binary alloys such as AlGaN. Here is the generalized form:

2 $$ P_{sp} (Al_{x} Ga_{1 - x} N) = xP_{sp} (AlN) + (1 - x)P_{sp} (GaN) $$

3 $$ P_{pz} (Al_{x} Ga_{1 - x} N) = 2e_{31} \frac{{a_{GaN} - a_{{Al_{x} Ga_{1 - x} N}} }}{{a_{{Al_{x} Ga_{1 - x} N}} }} + e_{33} \frac{{c_{{Al_{x} Ga_{1 - x} N}} - c_{GaN} }}{{c_{GaN} }} $$

where ${\mathrm{x}}$ is the composition of Al. ${\mathrm{e}}_{{{31}}}$ and ${\mathrm{e}}_{{{33}}}$ are effective piezoelectric constants for the mixed compound, often obtained by interpolating values between those of AlN and GaN based on composition. $\Delta {\mathrm{P}}$ stands for polarization difference. Its presence enables the formation of 2DEG at the AlGaN/GaN interface, which is crucial for high electron mobility in HEMTs.

4 $$ \Delta P = P_{total} (AlGaN) - P_{total} (GaN) $$

**Fig. 3:**
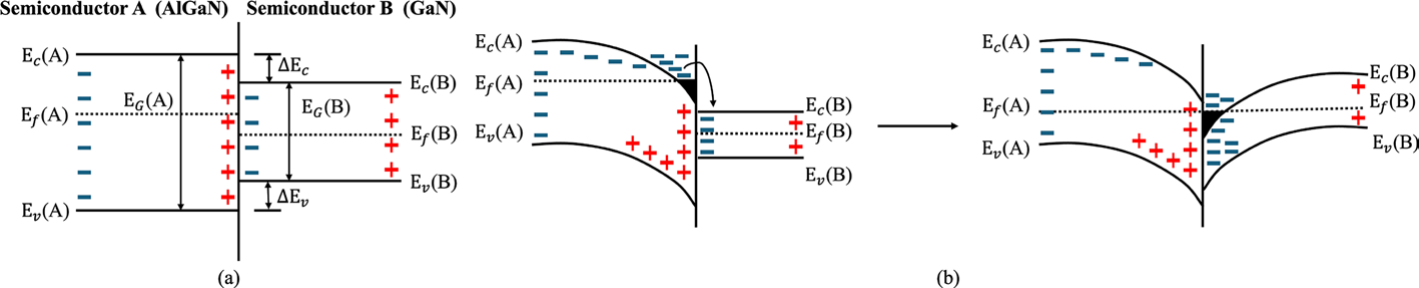
**a** Electron distribution at the heterojunction [170]. **b** Energy band changes and electron flow direction of AlGaN (left), and 2DEG at the AlGaN/GaN heterostructure after Fermi level equalization (right) [92]

The polarization reaction will eventually affect the energy band change of the material at the interface. Under this influence, the AlGaN/GaN interface is positively charged and the top of the AlGaN layer is negatively charged, as shown in Fig. 3(a) [170].

AlGaN, with a higher bandgap than GaN, creates a band alignment that naturally causes an energy band offset at the interface. This offset, combined with the built-in polarization effects in the AlGaN/GaN structure, generates an electric field that tilts the energy bands, driving electrons toward the GaN side. In the AlGaN layer, the polarization field tilts the bands more steeply, forcing electrons to migrate toward the interface. A large number of electrons will accumulate, resulting in a negative charge. When AlGaN is connected to GaN, the higher Fermi level in AlGaN compared to GaN causes electrons to flow from AlGaN to GaN, filling the conduction band edge of the GaN layer and forming the 2DEG, as shown in Fig. 3(b) [92]*.*

### Key parameters of HEMTs

Energy bandgap is a key factor in HEMT device performance because it directly influences the spontaneous polarization. Table 1 shows the characteristics of different HEMT materials at 300K [11, 42, 140, 166]. The WBG of GaN is approximately three times that of Si and GaAs [12, 41, 120], enabling it to handle higher electric fields, resulting in several advantages. Besides GaN, other WBG semiconductors such as SiC also exhibit unique material properties suitable for high-power and high-frequency applications. In particular, SiC offers a high breakdown field (5 MV/cm) and excellent thermal conductivity (4.9 W/cm·K), making it highly attractive for high-voltage and high-temperature environments such as electric vehicles and grid applications. On the other hand, it possesses an ultra-wide bandgap of 4.5 eV and a very high critical breakdown field (>7 MV/cm), indicating significant potential for next-generation power devices. However, its relatively low thermal conductivity (0.1–0.27 W/cm·K) remains a major challenge, limiting heat dissipation. While GaN is the central focus of this paper due to its superior electron mobility and high-frequency performance, these emerging materials also represent promising candidates for future power electronics research.

**Table 1:** Material properties of HEMT [31, 43, 61]

Parameters	Si	GaAs	GaN	4H-SiC	6H-SiC	$\beta -{Ga}_{2}{O}_{3}$
Bandgap (eV)	1.12	1.42	3.4	3.36	3.02	4.5
Dielectric Constant (εr)	11.8	13.1	8.9	9.7	9.7	10.2-12.4
Electron mobility (${\mathrm{cm}}^{{\mathrm{2}}} {\mathrm{/Vs}}$)	1350	8500	440	1000-1140	400-800	200
Breakdown field (${\mathrm{Mv/cm}}$)	0.3	0.4	3	5	5	>7
Thermal conductivity (${\mathrm{W/cm}} \cdot {\mathrm{k}}$)	1.5	0.5	1.3	4.9	4.9	0.11-0.27
Velocity ($\times {10}^{7}$ cm/s)	1	2	2.5	2	2	1.0-1.5

The energy band gap of semiconductor materials is related to temperature, and its calculation equation is [134]:

5 $$ E_{g} (T) = E_{g0} - \frac{{\alpha T^{2} }}{T + \beta } $$

where ${\mathrm{E}}_{{{\mathrm{g0}}}}$ is the bandgap energy at absolute zero temperature (0 K). ${\upalpha }$. and ${\upbeta }$ are material-dependent constants that adjust the temperature dependence based on the specific properties of the material. ${\mathrm{T}}$ is the temperature in Kelvin. For the group III-V materials of alloys, the dependence of the composition fraction ${\mathrm{x}}$ is described as follows:

6 $$ \begin{array}{*{20}c} {E_{g} \left( {A_{x} B_{1 - x} C} \right) = E_{g} \left( {AC} \right)x + E_{g} \left( {BC} \right)\left( {1 - x} \right) - bx\left( {1 - x} \right)} \\ \end{array} $$

A, B and C represent elements in the III-V group. ${\mathrm{E}}_{{\mathrm{g}}} \left( {{\mathrm{AC}}} \right)$ and ${\mathrm{E}}_{{\mathrm{g}}} \left( {{\mathrm{BC}}} \right)$ are the bandgap energies of the two pure materials. ${\mathrm{b}}$. is the bowing parameter specific to the AC-BC alloy system. In the bandgap equation for an alloy, the bowing parameter b adjusts the bandgap calculation to account for the complex electronic and structural interactions between the atoms of different elements in the alloy.

However, at a conductor-semiconductor junction, polarization is mainly caused by charge redistribution due to differences in work function and material properties [55]. Among these, the work function (Φ) represents the energy required to move electrons from the condensed solid surface to the external vacuum. In HEMT power devices, this parameter is essential for defining functional properties and device performance. Its equation is expressed as [161]:

7 $$ \begin{array}{*{20}c} {\Phi = E_{vac} - E_{f} } \\ \end{array} $$

where ${E}_{f}$ is the Fermi energy and ${E}_{vac}$ is the energy level in the vacuum. While the work function is a bulk property influenced by the entire material structure, electron affinity (χ) focuses specifically on the conduction band edge.

**Fig. 4:**
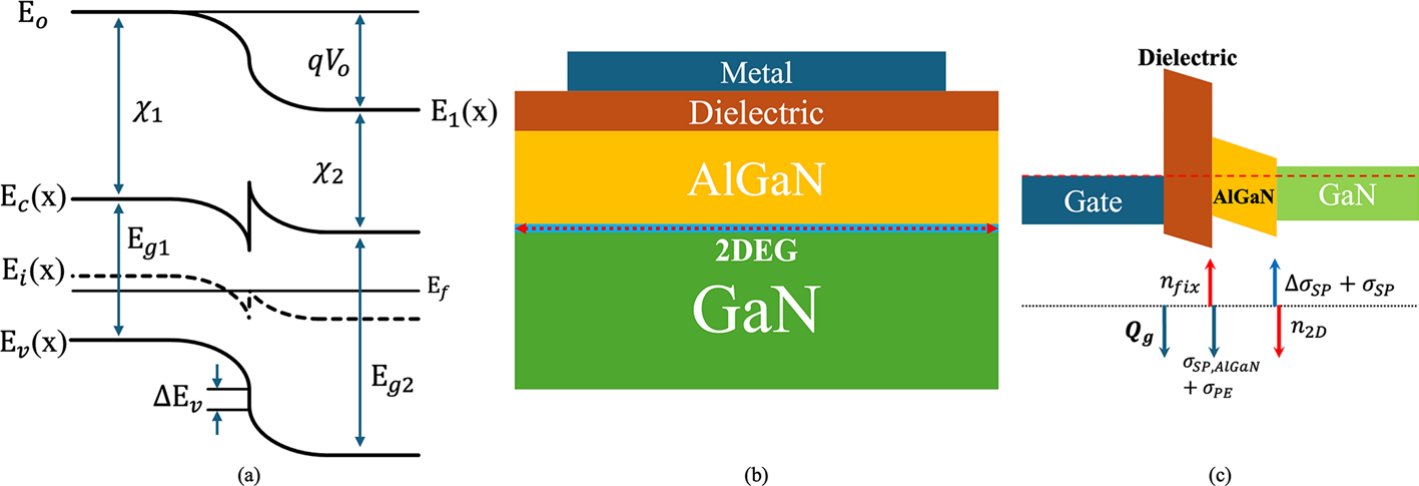
**a** Band diagram of heterojunction with band offset. **b** Metal-Dielectric HEMT structure and **c** corresponding electrostatic chart [37]

The interface between two semiconductor materials in a heterojunction is described by band alignment. Band alignment is characterized by two key parameters: band offset and electron affinity. Electron affinity refers to the energy difference between a material’s vacuum level and its conduction band minimum. This parameter is crucial in the analysis of heterojunctions, as it determines the alignment of conduction bands across different semiconductor materials. Figure 4(a) depicts a heterojunction formed by two semiconductors with differing bandgaps and electron affinities.

At the heterojunction, the conduction band offset ($\Delta {\mathrm{E}}_{{\mathrm{c}}}$), valence band offset ($\Delta {\mathrm{E}}_{{\mathrm{v}}}$. ), and the difference in bandgaps ($\Delta {\mathrm{E}}_{{\mathrm{g}}}$) can be expressed :

8 $$ \Delta E_{c} \begin{array}{*{20}c} { = \chi_{1} - \chi_{2} } \\ \end{array} $$

9 $$ \begin{array}{*{20}c} {\Delta E_{g} = E_{g2} - E_{g1} } \\ \end{array} $$

10 $$ \begin{array}{*{20}c} {\Delta E_{v} = \Delta E_{g} - \Delta E_{c} } \\ \end{array} $$

where ${\mathrm{E}}_{{{\mathrm{g1}}}}$ and ${\mathrm{E}}_{{{\mathrm{g2}}}}$ represent the bandgaps of the two materials. The $\Delta {\mathrm{E}}_{{\mathrm{c}}}$ is determined by the difference in electron affinities, while the $\Delta {\mathrm{E}}_{{\mathrm{v}}}$ accounts for the remaining difference in the band structures. This alignment creates a built-in potential and is crucial in optimizing the performance of HEMT devices, where such heterostructures enhance carrier confinement and transport properties.

Permittivity is a quantity used to describe dielectric properties that influence the reflection of electromagnetic waves at interfaces and the attenuation of wave energy within materials [90]. ${\upvarepsilon }$ is the permittivity tensor of a material and can be written as:

11 $$ \begin{array}{*{20}c} {\varepsilon = \varepsilon_{0} \varepsilon_{r} = \varepsilon_{0} \left[ {\begin{array}{*{20}c} {\varepsilon_{xx} } & {\varepsilon_{xy} } & {\varepsilon_{xz} } \\ {\varepsilon_{yx} } & {\varepsilon_{yy} } & {\varepsilon_{yz} } \\ {\varepsilon_{zx} } & {\varepsilon_{zy} } & {\varepsilon_{zz} } \\ \end{array} } \right]} \\ \end{array} $$

where ${\upvarepsilon }_{{0}}$ denotes the permittivity of free space, while ${\upvarepsilon }_{{\mathrm{r}}}$ is the relative permittivity, expressed as a dimensionless matrix-valued function over the domain Ω. The matrix ${\upvarepsilon }_{{\mathrm{r}}}$ contains nine elements that describe the coupling strength between the applied electric field and the resulting internal charge polarization. The subscripts specify the directions of the electric field and the induced polarization [26].

The energy bandgap, work function, electron affinity, and permittivity are fundamental intrinsic properties that govern the behavior of semiconductor materials. Understanding these parameters is critical for analyzing material interfaces and heterojunction performance. Building on this foundation, an accurate measurement model for the 2DEG density (${n}_{s}$) in AlGaN/GaN HEMTs becomes essential. This model links the 2DEG density to the gate voltage (${V}_{g}$), providing a quantitative framework to predict and optimize device performance in high-power and RF applications. Khandelwal et al. introduced an analytical model based on the first quantum energy level (${E}_{0}$) but neglected AlGaN layer charges [84]. Cheng et al. employed numerical methods to account for these carriers but faced limitations in circuit simulation [29]. A new unified analytical model eliminates recursive dependencies by using a fitting parameter, making ${n}_{b}$ in the AlGaN layer directly dependent on ${V}_{G}$. While charge-based drain current models [80, 145, 146, 188] are popular, many rely on semi-empirical ${n}_{s}$ models and overlook AlGaN charges, limiting their robustness for simulation. The tensile strain from growing AlGaN on GaN induces a piezoelectric polarization charge, ${\sigma }_{PE}$, which contributes to the spontaneous polarization charge, ${\upsigma }_{{{\mathrm{SP}}}}$, resulting in a positive net polarization charge at the AlGaN/GaN interface. Figure 4(b) shows the metal-dielectric HEMT structure. At the dielectric/AlGaN interface, ${\upsigma }_{{{\mathrm{PE}}}}$ and ${\upsigma }_{{{\mathrm{SP}}}}$. produce a negative net polarization charge, which is neutralized by positive charges, as illustrated in Fig. 4(c) [37]. The fixed charge density at the dielectric/AlGaN interface,, can be expressed as [67]:

12 $$ \begin{array}{*{20}c} {n_{fix} = n_{2D} + \Delta \sigma_{SP,diff} - Q_{g} /q} \\ \end{array} $$

where ${\mathrm{n}}_{{{\mathrm{2D}}}}$ represents 2DEG charge density, $\Delta {\upsigma }_{{{\mathrm{SP}}}}$ denotes the net spontaneous polarization charges at the AlGaN/GaN interface, $\Delta {\upsigma }_{{\mathrm{SP,diff}}}$ indicates the difference in the net spontaneous polarization charges between AlGaN and GaN layers, and ${\mathrm{Q}}_{{\mathrm{g}}}$ corresponds the charge in the metal gate.

The accurate determination of the two-dimensional electron gas (2DEG) concentration is essential for HEMT modeling, since it directly impacts channel conductivity and overall device performance. Several physics-based models have been proposed, each with different levels of analytical rigor and computational efficiency. Khandelwal et al. assumed that the quantum trap is a triangle and calculated the solutions of the Schrödinger equation and Poisson equation [83]. The ${\mathrm{n}}_{{{\mathrm{2D}}}}$ self-consistent solutions of two important energy levels are [89]:

13 $$ \begin{array}{*{20}c} {n_{2D} = DV_{th} \left\{ {\ln \left[ {exp\left( {\frac{{E_{f} - E_{0} }}{{V_{th} }}} \right)} \right] + \ln \left[ {exp\left( {\frac{{E_{f} - E_{0} }}{{V_{th} }}} \right) + 1} \right]} \right\}} \\ \end{array} $$

14 $$ \begin{array}{*{20}c} {E_{0} = \gamma_{0} n_{2D}^{\frac{2}{3}} , E_{1} = \gamma_{1} n_{2D}^{\frac{2}{3}} } \\ \end{array} $$

15 $$ \begin{array}{*{20}c} {n_{2D} = \frac{\varepsilon }{qd}\left( {V_{go} - E_{f} - V_{x} } \right)} \\ \end{array} $$

where ${\mathrm{V}}_{{{\mathrm{go}}}}$ is the cut-off voltage, ${\mathrm{V}}_{{\mathrm{x}}}$ is the channel potential at any point x in the channel, ${\mathrm{E}}_{{0}}$ and ${\mathrm{E}}_{{1}}$ are potential of first and second energy levels, ${\mathrm{E}}_{{\mathrm{f}}}$ is the position of Fermi level, D is density of states, d is thickness of barrier layer, ${\upgamma }_{{0}}$ and ${\upgamma }_{{1}}$ are experimentally determined parameters respectively [95]*.*

**Fig. 5:**
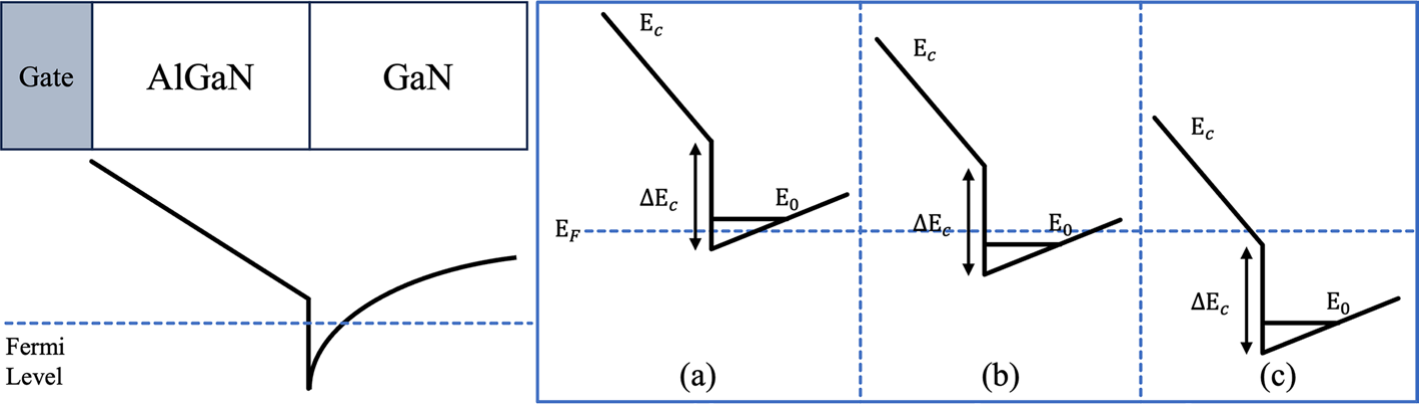
Energy band diagrams of three different regions **a** Region 1, **b** Region 2, and **c** Region 3 [81]

Karumuri et al. used numerical simulation to describe the variation of ${\mathrm{E}}_{{\mathrm{f}}}$ with gate voltage ${\mathrm{V}}_{{\mathrm{g}}}$, and this method was used as a basic model for 2DEG concentration [81]. It divides the working area into three according to the relative position of ${\mathrm{E}}_{{\mathrm{f}}}$ as: region 1: ${\mathrm{E}}_{{\mathrm{f}}} {\text{ < E}}_{{0}}$, region 2: ${\mathrm{E}}_{{0}} {\text{ < E}}_{{\mathrm{f}}} { < }\Delta {\mathrm{E}}_{{\mathrm{C}}}$, region 3: ${\mathrm{E}}_{{\mathrm{f}}} { > }\Delta {\mathrm{E}}_{{\mathrm{C}}}$, where $\Delta {\mathrm{E}}_{{\mathrm{C}}}$. is the conduction band discontinuity at the heterojunction as shown in Fig. 5. The model for ${\mathrm{n}}_{{{\mathrm{2D}}}}$ in a quantum well expressed as a function of ${\mathrm{E}}_{{\mathrm{f}}}$, in region 1, as given by:

16 $$ \begin{array}{*{20}c} {n_{2D} = DkTexp\left[ {\frac{{E_{f} - E_{0} }}{kT}} \right]} \\ \end{array} $$

where D is the 2D density of states inside the quantum well at the interface (with a typical value of ${10}^{{{14}}} {\text{ cm}}^{{ - 2}} \cdot {\mathrm{eV}}^{{ - 1}}$), $k$ is Boltzmann’s constant, and ${\mathrm{T}}$ is the absolute temperature in Kelvin. In region 2 and 3, the ${\mathrm{n}}_{{{\mathrm{2D}}}}$ are shown as:

17 $$ \begin{array}{*{20}c} {n_{2D} = D\left( {E_{f} - E_{0} } \right)} \\ \end{array} $$

At the same time, since the AlGaN layer, as a current barrier, normally does not contain mobile carriers, it can also be described as follows using parallel plate capacitor theory [33]:

18 $$ \begin{array}{*{20}c} {n_{2D} = \left[ {\frac{\varepsilon }{qd}} \right]\left( {V_{g} - V_{off} - \frac{{E_{f} }}{q} - V_{y} } \right)} \\ \end{array} $$

where ${\upvarepsilon }$ and ${\mathrm{d}}$ are the permittivity and thickness of the AlGaN layer, ${\mathrm{q}}$ is the Coulomb charge, and ${\mathrm{V}}_{{\mathrm{y}}}$ is the potential at any point in the 2DEG channel region (below the gate). ${\mathrm{V}}_{{{\mathrm{off}}}}$ is the off-voltage, derived theoretically using the equation given in [81].

N. Somashekar et al. developed a 2DEG concentration model that does not use any interpolation functions or fitting parameters [169]. The 2DEG concentrations is as follows:

19 $$ n_{2D} = \left[ {\frac{{AV_{g0} }}{1 + B}} \right]\left( {1 - \frac{{A^{\frac{2}{3}} \gamma_{0} }}{{\left[ {\left( {1 + B} \right)^{\frac{2}{3}} V_{g0}^{\frac{1}{3}} } \right]}}} \right) $$

where ${\text{A = }}{{\upvarepsilon } \mathord{\left/ {\vphantom {{\upvarepsilon } {\mathrm{(qd)}}}} \right. \kern-0pt} {\mathrm{(qd)}}}$, ${\mathrm{V}}_{{{\mathrm{g0}}}} {\text{ = V}}_{{\mathrm{g}}} {\text{ - V}}_{{{\mathrm{off}}}}$. , ${\text{B = A/(qd)}}$. , and ${\upgamma }_{{0}}$ is a parameter that determines the nature of variation of ${\mathrm{n}}_{{{\mathrm{2D}}}}$ with ${\mathrm{E}}_{{\mathrm{f}}}$ and takes into account the assumption of a quasi-constant electric field under the triangular well approximation [33].

A comparison of these models is provided in Table 2, highlighting the progression from quantum well approximations to region-based numerical approaches, and finally to efficient analytical formulations.

**Table 2:** Comparison of 2DEG electron concentration models

Refs	Model/Approach	Key features
Khandelwal [83]	Triangular quantum well approximation	Self-consistent Schrödinger–Poisson solution; analytical expressions for ${n}_{2D}$; reduced reliance on fitting parameters.
Karumuri [81]	Region-based numerical model (Schrödinger–Poisson)	Divides operation into 3 regimes based on ${E}_{f}$, illustrated in Fig. 5; uses quantum well approximation in region 1.
N. Somashekar [169]	Analytical quasi-constant electric field model	No interpolation or fitting parameters; computationally efficient; suitable for TCAD applications.

**Fig. 6:**
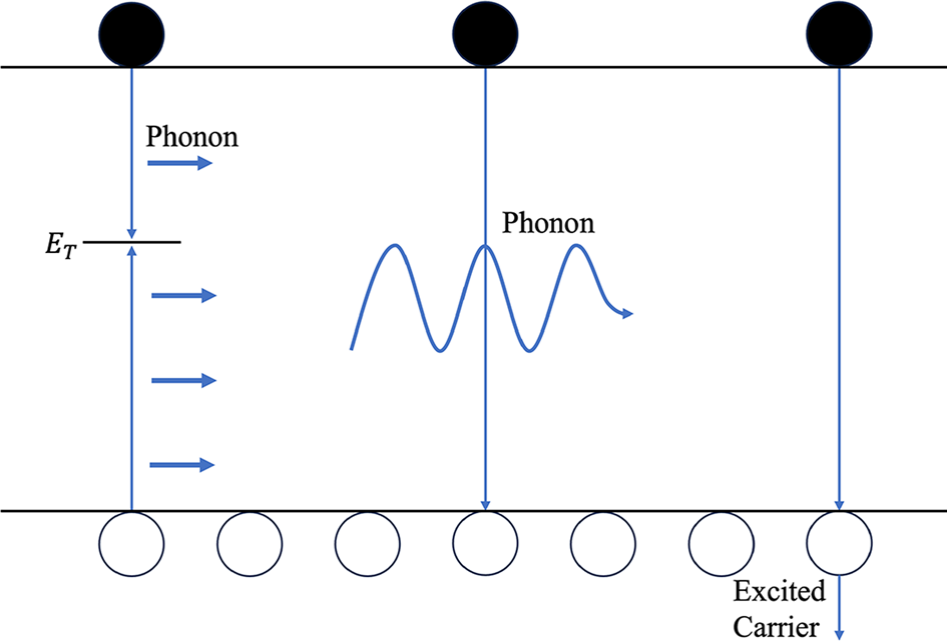
the carrier lifetime mechanisms: **a** SRH, **b** radiative and **c** Auger [8]

In semiconductor manufacturing, molecular contamination becomes increasingly critical as device dimensions shrink [48]. The composite lifetime measurement serves as an effective method to assess wafer contamination levels [141]. When the defect density in semiconductor devices is exceptionally low, the lifetime parameter stands out as one of the few indicators capable of providing valuable insights into such low concentrations. No other technique can detect defect densities as low as ${10}^{9}-{10}^{11}$ through simple, non-contact, room-temperature measurements. Furthermore, the lifetime parameter, in principle, has the potential to characterize defect densities approaching infinitesimal levels. The recombination mechanisms influencing carrier lifetime can be broadly classified into three categories: Shockley-Read-Hall (SRH) recombination, radiative recombination, and Auger recombination. The SRH theory, proposed in the 1950s by Shockley and Read and later developed by Hall, describes electron-hole pair recombination through defects or traps, characterized by the carrier lifetime ${\tau }_{srh}$ [56, 163]. Radiative recombination is associated with photon emission and is characterized by ${\tau }_{rad}$. Auger recombination, on the other hand, involves the transfer of energy to a third carrier and is characterized by ${\tau }_{Auger}$. These three mechanisms are illustrated in Fig. 6 [8].

The overall carrier lifetime $\tau$ is determined by the combined effects of these mechanisms, as expressed by the following relationship:

20 $$ \frac{1}{\tau } = \frac{1}{{\tau_{rad} }} + \frac{1}{{\tau_{Auger} }} + \frac{1}{{\tau_{srh} }} $$

SRH recombination involves carrier recombination through deep-level defects or impurities. This mechanism is governed by the defect density ${N}_{T}$, defect energy level ${E}_{T}$, and capture cross-sections ${\sigma }_{n}$ and ${\sigma }_{p}$ for electrons and holes, respectively. The SRH lifetime ${\tau }_{srh}$ is expressed as:

21 $$ \tau_{srh} = \frac{{\tau_{p} \left( {n_{o} + n_{1} + \Delta n} \right) + \tau_{n} \left( {p_{o} + p_{1} + \Delta p} \right)}}{{p_{o} + n_{o} + \Delta n}} $$

22 $$ n_{1} = n_{i} e^{{\left( {\frac{{E_{T} - E_{i} }}{kT}} \right)}} $$

23 $$ p_{1} = n_{i} e^{{\left( {\frac{{E_{i} - E_{T} }}{kT}} \right)}} $$

24 $$ \tau_{n} = \frac{1}{{\sigma_{n} V_{th} N_{T} }} $$

25 $$ \tau_{p} = \frac{1}{{\sigma_{p} V_{th} N_{T} }} $$

${\upsigma }$ is the capture cross-section of defect states. ${\mathrm{N}}_{{\mathrm{T}}}$ is the defect density. ${\mathrm{v}}_{{{\mathrm{th}}}}$ is the thermal velocity of the carriers, and ${\mathrm{n}}_{{\mathrm{i}}}$ is the intrinsic carrier concentration.

In radiative recombination, carriers transition directly from the conduction band to the valence band, releasing energy as photons. The radiative lifetime ${\uptau }_{{{\mathrm{rad}}}}$ is inversely proportional to the carrier density and is given by [177]:

26 $$ \tau_{rad} = \frac{1}{{B(p_{o} + n_{o} + \Delta n)}} $$

where B is the radiative recombination coefficient. ${\mathrm{n}}_{{\mathrm{o}}}$ and ${\mathrm{p}}_{{\mathrm{o}}}$ are the equilibrium carrier concentration. $\Delta {\mathrm{n}}$ is an excess carrier density. The radiative lifetime is inversely proportional to the carrier density, as band-to-band recombination requires the simultaneous presence of both electrons and holes. Auger recombination occurs when the recombination energy is absorbed by a third carrier, either an electron or a hole. The Auger lifetime ${\uptau }_{{{\mathrm{Auger}}}}$ decreases with increasing carrier density and is given by:

27 $$ \tau_{Auger} = \frac{1}{{C_{P} \left( {P_{o}^{2} + 2P_{0} \Delta n + \Delta n^{2} } \right) + C_{n} (n_{o}^{2} + 2n_{o} \Delta n + \Delta n^{2} )}} $$

where ${\mathrm{C}}_{\mathrm{n}}$ and ${\mathrm{C}}_{\mathrm{P}}$ are the Auger recombination coefficient for electrons and holes.

### HEMT variant structures

As discussed above, the 2DEG in a HEMT arises mainly from spontaneous polarization. In a conventional depletion-mode (D-mode) HEMT, this results in a conducting channel even at zero gate bias, making the device “normally-on.” While such devices can achieve high current density and low on-resistance, their normally-on nature presents challenges for power electronics. Specifically, additional negative gate bias is required to switch the device off, complicating circuit design. Moreover, in high-voltage systems, a normally-on device may pose safety concerns such as shoot-through current at startup, limiting its practical adoption. These issues highlight the broader limitations of D-mode HEMTs, including reduced compatibility with standard gate drivers and lower system reliability in fail-safe operation. To address these challenges and expand their applicability, several different HEMT structures have been proposed.

**Fig. 7:**
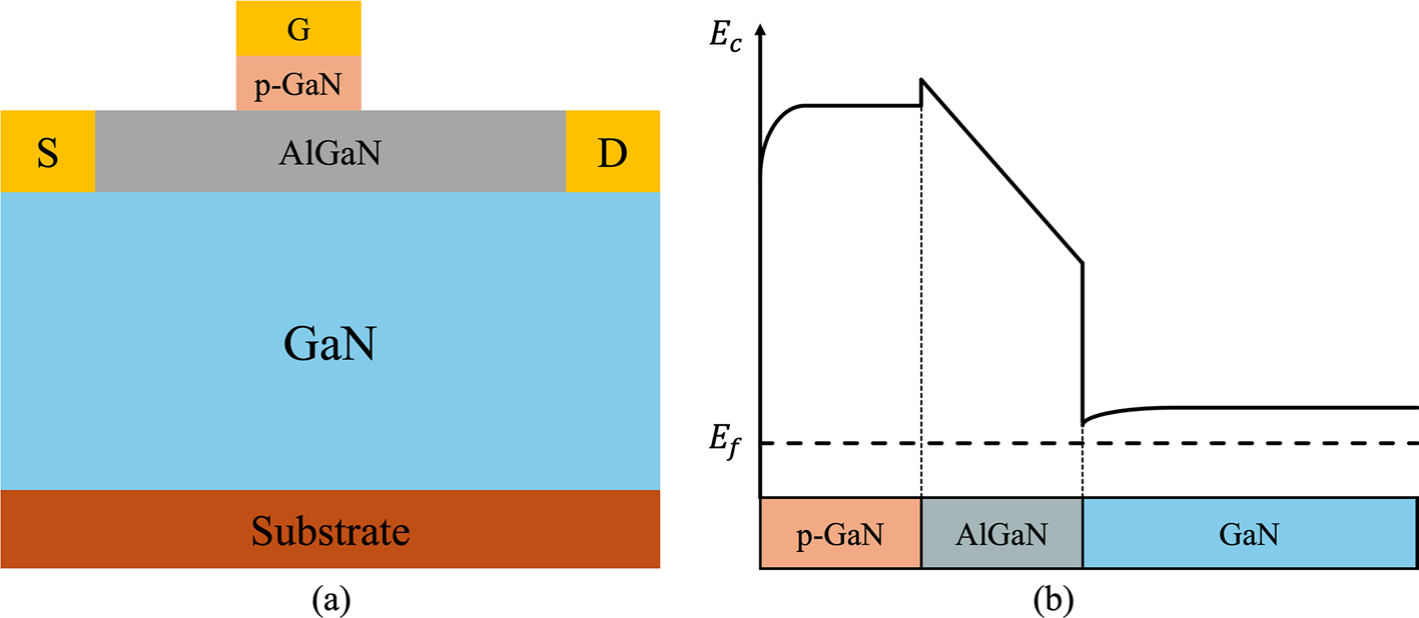
**a** p-GaN HEMT structure and **b** conduction band energy diagram

**Fig. 8:**
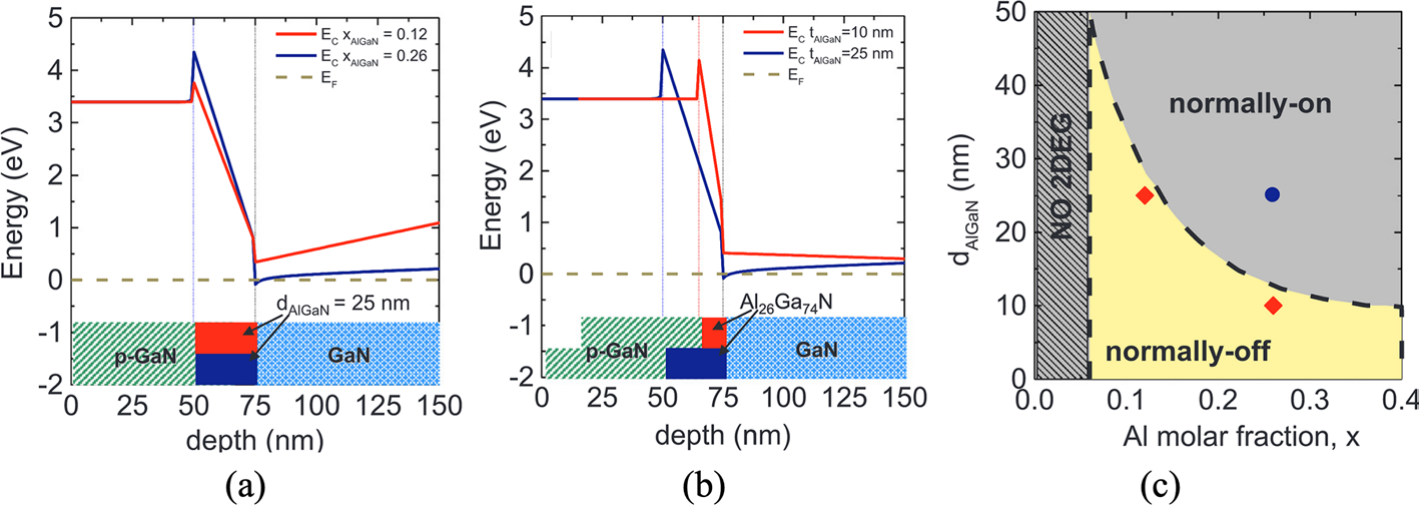
Conduction band energy diagrams in p-GaN/AlGaN/GaN HEMTs: **a** varying Al Composition, **b** varying barrier layer thickness [51], and **c** Al composition vs. AlGaN thickness for normally-On/Off boundary [45]. Copyright 2017 Elsevier

The p-GaN gate HEMT is a widely used enhancement-mode (E-mode) device. Its structure, derived from the basic AlGaN/GaN HEMT shown in Fig. 2(a), incorporates a p-doped GaN layer between the gate and the AlGaN barrier. This p-GaN layer elevates the conduction band under the gate region, shifting the 2DEG energy above the Fermi level and thereby achieving normally-off operation, as shown in Fig. 7(a) and (b). The thickness and Al composition of the AlGaN barrier layer are critical parameters that determine whether a HEMT operates in normally-off mode. Giuseppe et al. analyzed their effects on the conduction band energy of a p-GaN/AlGaN/GaN HEMT, as shown in Fig. 8(a) and (b) [51]. In their study, the p-GaN layer had a thickness of 50 nm and a doping concentration of $3\times {10}^{19} {cm}^{-3}$. The results show that reducing the Al composition in the AlGaN barrier weakens the spontaneous polarization effect, preventing the conduction band edge at the 2DEG from falling below the Fermi level and thereby achieving normally-off operation. Similarly, decreasing the AlGaN barrier thickness reduces polarization strength, which raises the conduction band energy above the Fermi level, ensuring that the device remains normally-off.

Furthermore, Fujii et al. mapped the boundary conditions of AlGaN barrier thickness and Al composition that distinguish normally-off from normally-on HEMTs, as shown in Fig. 8(c) [45]. Their simulations revealed the ranges of barrier parameters where the conduction band edge remains above the Fermi level, ensuring normally-off operation. To validate these findings, they fabricated HEMTs with different AlGaN barrier layer thicknesses and Al compositions, demonstrating that precise control of these parameters is essential for defining the HEMT operating mode.

**Fig. 9:**
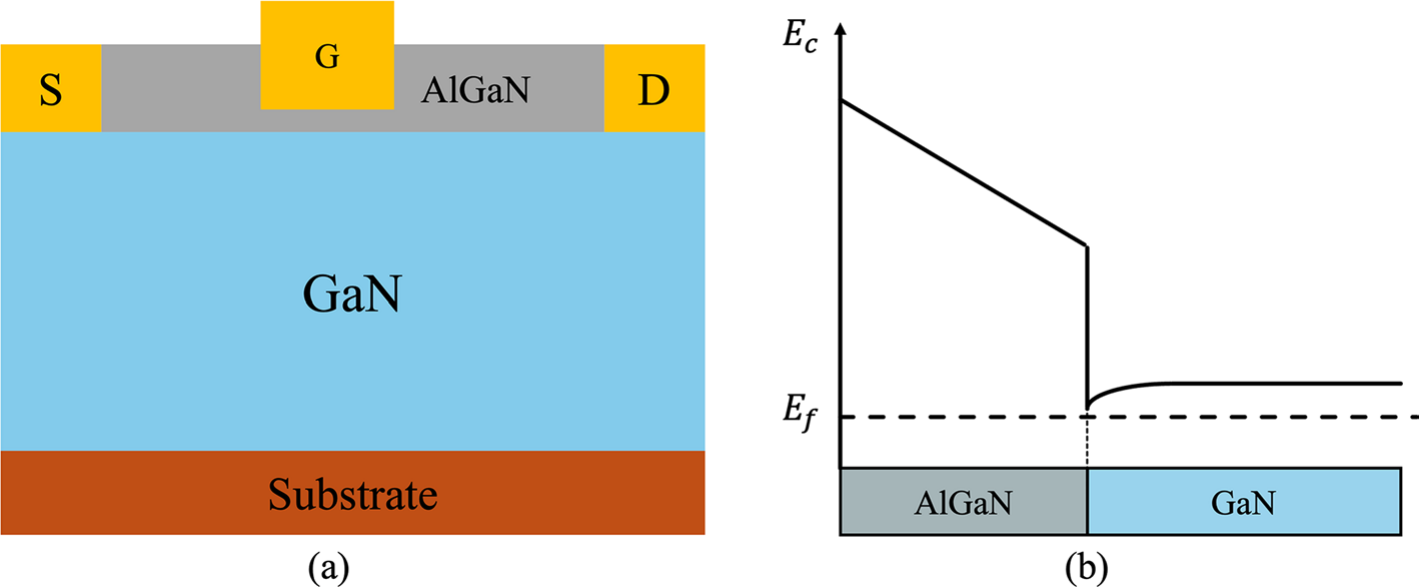
**a** Recessed-gate HEMT structure and **b** conduction band energy diagram

The recessed-gate HEMT is another E-mode structure. In this design, a portion of the AlGaN barrier is selectively etched before depositing the gate, resulting in a thinner barrier under the gate region, as shown in Fig. 9(a). The reduced barrier thickness weakens the spontaneous polarization effect, raising the conduction band edge above the Fermi level and thereby realizing normally-off operation, as shown in Fig. 9(b).

**Fig. 10:**
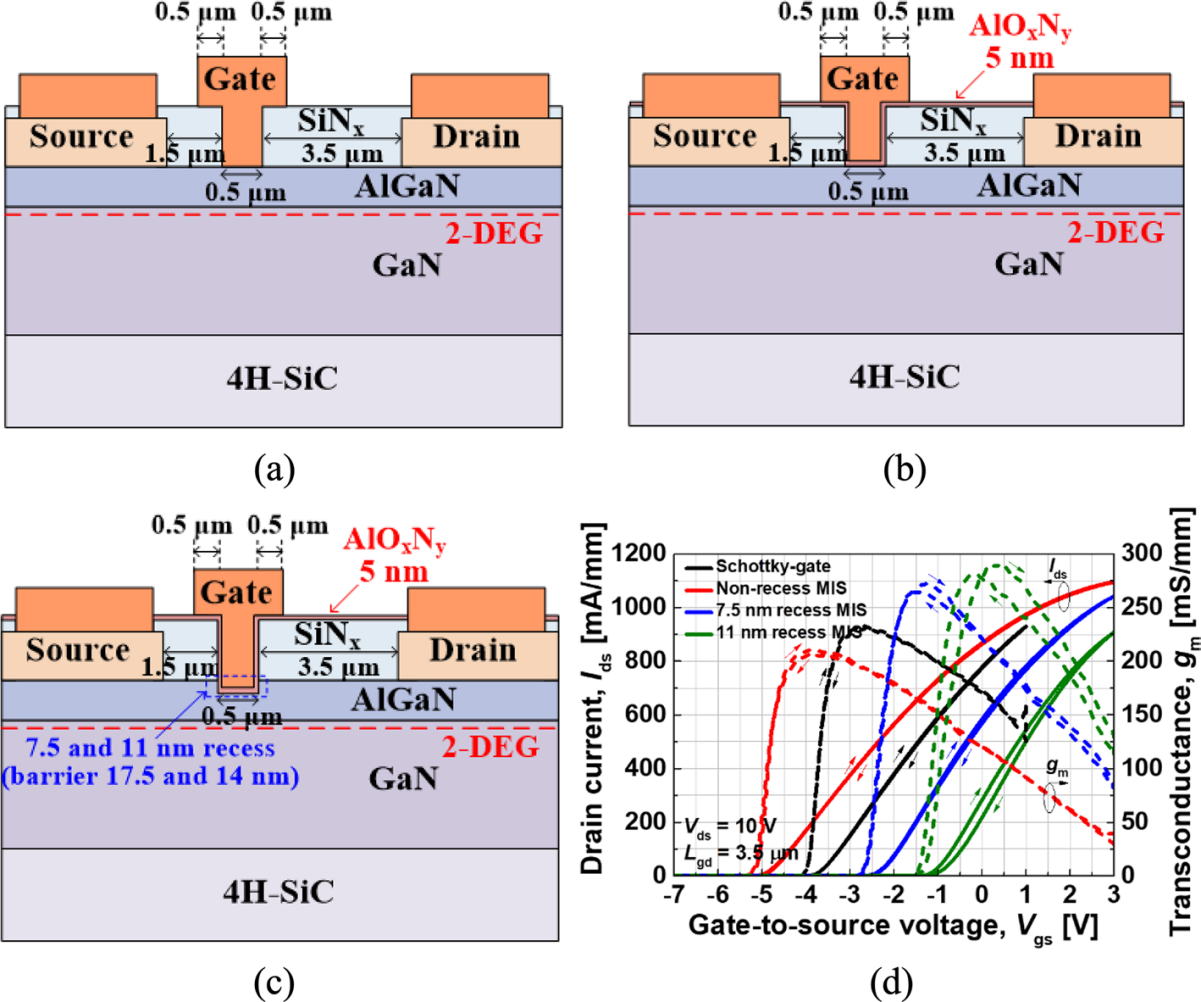
Schematic cross-section of AlGaN/GaN HEMTs: **a** Schottky gate, **b** non-recessed MIS-HEMT, and **c** recessed MIS-HEMT. **d** transfer and transconductance characteristics [85]. Copyright 2023 MDPI

Compared to p-GaN HEMTs, recessed-gate HEMTs achieve normally-off operation by etching the AlGaN barrier layer beneath the gate to a very thin thickness. Kim et al. investigated the impact of recessed gates on AlGaN/GaN MIS-HEMTs grown on SiC substrates [85]. As shown in Fig. 10, (a) depicts a Schottky-gate HEMT, (b) a MIS-HEMT without recesses using a 5-nm AlxOy insulator, and (c) a recessed MIS-HEMT, while (d) compares their ID–VG and transconductance characteristics. The threshold voltage of the MIS-HEMT without recesses is slightly lower than that of the Schottky-gate HEMT, shifting from about − 4 V to − 5 V. Introducing a recessed gate raises the threshold voltage, increasing from − 4 V (Schottky-gate HEMT) to − 2.2 V for a 7.5-nm recess and to − 0.8 V for an 11-nm recess. It is worth noting that the 7.5-nm recessed HEMT retains a thicker barrier (17.5 nm) compared to the 14-nm barrier in the 11-nm recessed HEMT, which partly limits the threshold voltage shift. Overall, recessed gates provide only a moderate increase in threshold voltage, but they also enhance maximum transconductance due to the thinner effective AlGaN barrier under the gate, offering improved device performance while moving closer to a normally-off state.

Metal–Insulator–Semiconductor (MIS) HEMTs and Metal–Oxide–Semiconductor (MOS) HEMTs differ primarily in the dielectric material inserted between the gate and the barrier layer. For MIS-HEMTs, common dielectrics include SiN and Al₂O₃, while MOS-HEMTs typically employ oxides such as SiO_2_, Al_2_O_3_, HfO_2_, or ZrO_2_. When an oxide is used, the distinction between MIS- and MOS-HEMTs becomes largely nominal, as their characteristics are nearly identical. Introducing a dielectric layer brings important fabrication trade-offs: it effectively suppresses gate leakage current, improves threshold voltage stability, and enhances device reliability under high electric fields, but at the same time adds complexity to the gate stack processing and may introduce additional interface states that degrade mobility if not carefully optimized. Despite these challenges, MIS- and MOS-HEMTs offer several advantages over conventional Schottky-gate HEMTs, including higher breakdown voltage, lower leakage, and improved thermal stability. For instance, Anant et al. investigated GaN-on-Si power MIS-HEMTs with SiN as the dielectric, demonstrating stable noise performance across a wide temperature range, highlighting their robustness for high-power and high-temperature applications [76]*.*

Cascade and cascode HEMTs are structural innovations designed to overcome the normally-on limitation of conventional D-mode GaN HEMTs while retaining their high-performance advantages. In a cascade HEMT, a GaN HEMT is monolithically integrated with a low-voltage Si MOSFET, where the MOSFET ensures normally-off behavior and the GaN HEMT provides high electron mobility and breakdown strength. In contrast, a cascode HEMT connects a normally-on GaN HEMT in series with a low-voltage Si MOSFET in a discrete configuration, achieving normally-off operation without heavily modifying the GaN structure and taking advantage of existing Si processing technologies. While both approaches reduce gate-driving complexity and expand applicability in power electronics, trade-offs remain: cascade devices face challenges in monolithic integration, whereas cascode devices suffer from increased parasitics at the Si–GaN interface. Beyond structural trade-offs, reliability concerns also arise. Under high-field stress, these devices experience structural damage near the gate and defect accumulation in the barrier layer, with long-term stress increasing intrinsic defect concentrations and negative gate bias altering transconductance. Irradiation effects further complicate reliability: total ionizing dose irradiation may reduce defect density, while heavy-ion incidence dramatically increases it, causing severe degradation. These issues highlight the sensitivity of cascade and cascode HEMTs to electrical and radiation-induced stress. To address these challenges, Su et al. conducted in-depth research on threshold voltage and transconductance degradation mechanisms under high-field off-state stress, providing a detailed physical explanation of co-gate degradation [167]. Their findings establish a theoretical foundation for improving the high-voltage reliability design of cascode GaN HEMT power devices.

Table 3 provides a comparative overview of representative GaN HEMT structures along with their typical performance parameters, including threshold voltage (${V}_{th}$), saturation drain current density (${I}_{sat}$), and on-resistance (${R}_{on}$). These values illustrate the trade-offs between different structural approaches. For example, p-GaN HEMTs generally achieve positive threshold voltages, while cascode and MOS-HEMT variants can support higher current densities at the cost of greater structural complexity. Such parameters are critical for assessing device suitability in high-power and high-frequency applications. Beyond performance benchmarking, different HEMT variants have been developed to address specific design challenges, each finding applications in distinct areas of power and RF electronics. p-GaN HEMTs, with their normally-off operation, are widely adopted in commercial power supplies and fast chargers, where circuit simplicity and safety are critical. Recessed-gate HEMTs offer improved threshold voltage control and are increasingly used in high-voltage switching applications such as electric vehicle inverters and industrial motor drives [107]. MIS-HEMTs and MOS-HEMTs, which incorporate dielectric layers under the gate, provide enhanced gate reliability and reduced leakage, making them suitable for harsh-environment applications, including automotive and aerospace systems. Cascode HEMTs, combining GaN with low-voltage Si MOSFETs, are commonly deployed in data centers and renewable energy converters, where efficient high-voltage switching is required, while cascade HEMTs have potential in monolithic integration for compact, high-efficiency power modules [181, 192]. These applications illustrate how structural innovations in GaN HEMTs not only determine performance trade-offs but also enable tailored solutions for both high-frequency RF domains and high-efficiency power conversion systems. While this section introduces the structures, their characteristic parameters, and representative applications, the specific role of these parameters and their impact on simulation accuracy will be discussed in detail in Sect. 4, where the physical models and parameter dependencies are analyzed.

**Table 3:** HEMT structures and typical parameters

Refs	HEMT structure	Mode	${V}_{th} (V)$	${I}_{sat}$ (A/mm)	${\mathrm{R}}_{{on}}$ $(\Omega \cdot {\mathrm{mm}})$
[46]	p-GaN/AlInN/AlN/GaN	E-mode (p-GaN)	0.2	1.99	1.507
[74]	AlN/p-GaN/AlGaN/GaN	E-mode (p-GaN)	0.82	0.048	67.5
[99]	p-GaN/AlGaN/GaN	E-mode (p-GaN)	1.4	0.12	-
[186]	Al_2_O_3_/AlGaN/GaN	MIS-HEMT (Recessed Gate)	1	1.5	-
[65]	ALD-SiN_x_/GaN/AlGaN/AlN/GaN	MIS-HEMT	-1.9	1.2	-
[98]	HfO_2_/GaN/AlGaN/GaN	MOS-HEMT	-3.92	0.3	22
[73]	SrTiO_3_/AlGaN/GaN	MOS-HEMT	-3.3	26	1
[167]	AlGaN/GaN	MOS-Gate Cascode HEMT	4.43	15	-
[64]	AlGaN/GaN	Cascode HEMT	2.5	0.6	-

## TCAD basic physical model simulation

Silvaco TCAD, particularly the Victory Device simulator, offers a robust platform for modeling semiconductor devices by solving coupled nonlinear partial differential equations (PDEs), including Poisson’s equation, electron and hole continuity equations, and heat flow and energy transport equations. These equations are derived from fundamental principles, such as Maxwell’s laws, and capture the interplay between electric potential, carrier densities, and underlying physical processes. The simulator discretizes these continuous models into nonlinear algebraic systems on a computational grid, which are solved iteratively using linearization and refinement techniques until convergence is achieved. By leveraging TCAD, researchers can accurately model the physical characteristics of semiconductor devices, enabling detailed analysis, optimization, and performance prediction for advanced devices like HEMTs. This demonstrates the indispensable role of TCAD in driving innovation across a wide range of semiconductor technologies.

### Poisson’s equation

Poisson’s equation establishes the relationship between the variation in electrostatic potential and the local charge density. The numerical solution of the nonlinear Poisson equation is traditionally achieved through the application of Newton’s method to the discretized equations. While effective, this method exhibits convergence only when the initial guess is sufficiently close to the true solution. Moreover, its implementation requires solving a system of linear algebraic equations at each iteration, imposing significant computational demands. The need for fine grid subdivision further exacerbates these requirements, necessitating the use of high-performance computing resources with substantial memory capacity, particularly in semiconductor device modeling applications [40].

**Fig. 11:**
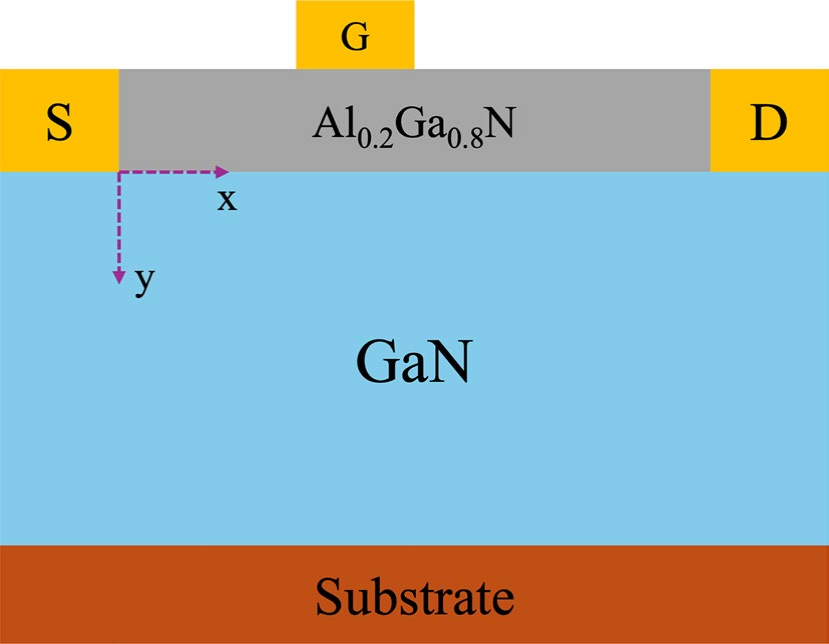
cross-sectional schematic of B-HEMT structure

Figure 11 and Table 4 present the cross-sectional schematic of the bare HEMT (B-HEMT) structure along with its corresponding dimensions and key parameters. The buffer layer is grown on a sapphire substrate using a two-step epitaxial growth process. Initially, a 1.2 μm thick GaN layer is deposited, followed by the growth of a 2.2 μm thick GaN layer. Subsequently, a 22 nm thick AlGaN layer with an aluminum composition of 20% is deposited on top of the GaN layer. The source-to-gate (${\mathrm{L}}_{{{\mathrm{sg}}}}$) and gate-to-drain (${\mathrm{L}}_{{{\mathrm{gd}}}}$) spacings are 2 μm and 5 μm, respectively. The gate length (${\mathrm{L}}_{{\mathrm{g}}}$), drain length (${\mathrm{L}}_{{\mathrm{d}}}$), and source length (${\mathrm{L}}_{{\mathrm{s}}}$) are 3 μm, 2 μm, and 2 μm, respectively.

**Table 4:** dimensions and parameters of B-HEMT

Parameter	Unit	Value	Description
${L}_{s}$	μm	2	Length of source electrode
${L}_{g}$	μm	3	Length of gate electrode
${L}_{d}$	μm	2	Length of drain electrode
${L}_{sg}$	μm	2	Gate-to-source distance
${L}_{gd}$	μm	5	Gate-to-drain distance
${T}_{bar}$	nm	22	Thickness of AlGaN layer
${T}_{buff}$	μm	3.4	Thickness of buffer layer

Singh et al. developed a physics-based model for analyzing surface potential, electric field, and drain current in the channel region, derived from Poisson’s equation [165]. The Poisson equation in the GaN channel is expressed as [114]:

28 $$ \frac{{\partial^{2} \phi \left( {x,y} \right)}}{{\partial x^{2} }} + \frac{{\partial^{2} \phi \left( {x,y} \right)}}{{\partial y^{2} }} = \frac{q}{{\varepsilon_{g} }}\left( {n - p - D} \right) $$

where $\varphi \left( {\mathrm{x,y}} \right)$ is electric potential, ${\upvarepsilon }_{{\mathrm{g}}}$ is the permittivity of GaN, n and p are the mobile electron and hole densities, and D is the concentration of ionized impurities. Next, the electric potential along the y-direction can be approximated using a parabolic expression as:

29 $$ \begin{array}{*{20}c} {\phi = \varphi_{s} \left( x \right) + A\left( x \right)y + B\left( x \right)y^{2} } \\ \end{array} $$

where $\phi_{{\mathrm{s}}} \left( {\mathrm{x}} \right)$ represents the surface potential. A(x) and B(x) are arbitrary constants that are determined based on the applied boundary conditions. Due to the differing characteristics of the region beneath the gate compared to other areas, the potentials in these regions from source to drain must be calculated separately. The regions are divided into ${0} \le {\mathrm{x}} \le {\mathrm{L}}_{{{\mathrm{sg}}}}$, ${\mathrm{L}}_{{{\mathrm{sg}}}} \le {\mathrm{x}} \le {\mathrm{L}}_{{{\mathrm{sg}}}} {\text{ + L}}_{{\mathrm{g}}}$ and ${\mathrm{L}}_{{{\mathrm{sg}}}} {\text{ + L}}_{{\mathrm{g}}} \le {\mathrm{x}} \le {\mathrm{L}}_{{{\mathrm{sd}}}}$ (${\mathrm{L}}_{{{\mathrm{sd}}}}$ is the distance from source to drain). The boundary condition expressions for the electric potential are:

30 $$ \left. {\frac{{d\phi \left( {x,y} \right)}}{dy}} \right|_{y = 0} = \frac{{\varepsilon_{al} }}{{\varepsilon_{g} }}\frac{{\varphi_{s} \left( x \right) - V_{GS}{\prime} }}{{d_{1} }} $$

31 $$ \left. {\frac{{d\phi \left( {x,y} \right)}}{dy}} \right|_{{y = - d_{1} }} = \frac{{ - qn_{s} }}{{\varepsilon_{1} }} = - E $$

32 $$ \left. {\varphi_{s} \left( x \right)} \right|_{x = 0} = V_{bi} $$

33 $$ \left. {\varphi_{s} \left( x \right)} \right|_{{x = L_{sd} }} = V_{DS} + V_{bi} $$

34 $$ V_{GS}{\prime} = V_{GS} - V_{FB} $$

35 $$ V_{FB} = \Phi_{m} - \Phi_{s} = \Phi_{m} - \left( {\chi + \frac{{E_{C} }}{2}} \right) $$

where ${\mathrm{d}}_{{1}}$ is the thickness of AlGaN layer, q is the unit electron charge, ${\mathrm{n}}_{{\mathrm{s}}}$ is the 2DEG density, ${\upvarepsilon }_{{{\mathrm{al}}}}$ and ${\upvarepsilon }_{{\mathrm{g}}}$ are the permittivity of AlGaN and GaN, ${\mathrm{V}}_{{{\mathrm{bi}}}}$ is built-in potential, ${\mathrm{V}}_{{{\mathrm{FB}}}}$ is flat band voltage, $\Phi_{{\mathrm{m}}}$ is gate metal workfunction, ${\upchi }$ is electron affinity, ${\mathrm{E}}_{{\mathrm{c}}}$ is conduction band. Equations 30 and 31 represent the boundary conditions for the electric potential, while Eqs. 32 and 33 describe the boundary conditions for the surface potential. All these equations are derived based on Laplace’s equation [125]. In Eq. 35, the AlGaN barrier layer is assumed to be undoped, meaning that ${\mathrm{V}}_{{{\mathrm{FB}}}}$ is determined solely by $\Phi_{{\mathrm{m}}}$, ${\upchi }$, and ${\mathrm{E}}_{{\mathrm{C}}}$ [18].

The charge density of 2DEG caused by polarization is calculated as shown in Eq. (17) in chapter 2. Among them, ${V}_{go}={V}_{GS}-{V}_{OFF}$. The ${V}_{OFF}$ is given as:

36 $$ V_{OFF} = \Phi_{B} - \Delta E_{C} - \frac{{P_{T} }}{{C_{G} }} $$

where $\Phi_{{\mathrm{B}}}$ represents the Schottky barrier height, $\Delta {\mathrm{E}}_{{\mathrm{C}}}$ denotes the conduction band offset at the AlGaN/GaN heteojunction, ${\mathrm{P}}_{{\mathrm{T}}}$ is the total polarization charge density, and ${\mathrm{C}}_{{\mathrm{G}}}$ refers to the gate capacitance. By solving the boundary conditions outlined in Eqs. (30), (31), (32), (33 ) and ( 34), the constants A and B in Eq. (29) can be determined. Using these constants, the channel potential is expressed as:

37 $$ \phi \left( {x,y} \right) = \varphi_{s} \left( x \right) + \frac{{\varepsilon_{al} }}{{\varepsilon_{g} }}\left( {\frac{{\varphi_{s} \left( x \right) - V_{GS}{\prime} }}{{2d_{1} }}} \right)y + \left[ {\frac{{ - E_{1} }}{{2d_{1} }} + \frac{{\varepsilon_{al} }}{{\varepsilon_{g} }}\left( {\frac{{\varphi_{s} \left( x \right) - V_{GS}{\prime} }}{{2d_{1} }}} \right)} \right]y^{2} $$

By using Eq. (37) and substituting $y={d}_{1}$, the regions in the channel are as follows:

38 $$ \phi \left( {x,d_{1} } \right) = \varphi_{s} \left( x \right) + \frac{{\varepsilon_{al} }}{{\varepsilon_{g} }}\left( {\varphi_{s} \left( x \right) - V_{GS}{\prime} } \right) + \frac{{ - E_{1} }}{2} $$

The lateral electric field expression of the 2DEG channel is:

39 $$ E_{x} = - \frac{d\varphi \left( x \right)}{{dx}} $$

The electric field from $x=0$ to ${\mathrm{L}}_{\mathrm{sd}}$ is expressed as:

40 $$ \int\limits_{0}^{{{\mathrm{L}}_{{{\mathrm{sd}}}} }} {{\mathrm{E}}_{{\mathrm{x}}} {\mathrm{dx}} = - \left( {\varphi \left( {{\mathrm{L}}_{{{\mathrm{sd}}}} } \right) - \varphi \left( 0 \right)} \right)} $$

The drain current (${I}_{DS}$) expression is as follows:

41 $$ \frac{{{\mathrm{I}}_{{{\mathrm{DS}}}} \left( {\mathrm{x}} \right)}}{{\mathrm{W}}} = {\mathrm{qn}}_{{\mathrm{s}}} \left( {\mathrm{x}} \right){\mathrm{v}}_{{\mathrm{e}}} \left( {\mathrm{x}} \right) $$

where W is the gate width, ${\mathrm{v}}_{\mathrm{e}}\left({\mathrm{x}}\right)$ represents the electron effective velocity, and is expressed as follows:

42 $$ v_{e} \left( x \right) = \frac{{\mu_{n} E_{x} v_{sat} }}{{\mu_{n} E\left( x \right) + v_{sat} }} $$

43 $$ \mu_{n} = \frac{{\mu_{n.gan} + \frac{{v_{sat} }}{{E_{x} }}\left( {\frac{{E_{x} }}{{E_{C} }}} \right)^{\gamma } }}{{1 + \left( {\frac{{E_{x} }}{{E_{C} }}} \right)^{\gamma } }} $$

where ${\upmu }_{{\mathrm{n}}}$ is the electron mobility and its equation is 43, ${\upmu }_{{{\mathrm{n}}{\mathrm{.gan}}}}$ is the electron mobility of GaN, ${\mathrm{E}}_{{\mathrm{C}}}$ is critical field, ${\upgamma }$ is the. Substituting Eq. (15) into Eq. (41), the final expression of ${\mathrm{I}}_{{{\mathrm{DS}}}}$ is

44 $$ {\mathrm{I}}_{{{\mathrm{DS}}}} \left( {\mathrm{x}} \right) = {\mathrm{WC}}_{{\mathrm{G}}} \left( {{\mathrm{V}}_{{{\mathrm{go}}}} - {\mathrm{E}}_{{\mathrm{f}}} } \right)\frac{{{\upmu }_{{\mathrm{n}}} {\mathrm{E}}_{{\mathrm{x}}} {\mathrm{v}}_{{{\mathrm{sat}}}} }}{{{\upmu }_{{\mathrm{n}}} {\mathrm{E}}\left( {\mathrm{x}} \right) + {\mathrm{v}}_{{{\mathrm{sat}}}} }} $$

The electron mobility of GaN material at room temperature is approximately ${\text{1200 cm}}^{{2}} /\left( {{\mathrm{V}} \cdot {\mathrm{s}}} \right)$.[77], the critical electric field is around ${3}{\text{.3 MV}}/{\mathrm{cm}}$ [87, 113], and the dielectric constant is 8.9.

### Carrier continuity equations

Most studies related to the electrical properties of GaN-based HEMTs are limited to the equilibrium case, i.e., when zero gate voltage is applied [100, 1, 10, 14, 106, 191]. One possible reason for the limited studies on electron mobility in the literature could be the limitations of traditional Schrödinger-Poisson solvers. These solvers generally assume a constant quasi-Fermi level throughout the vertical cross-section of the HEMT structure, an assumption that is strictly valid only at equilibrium. For accurately simulating the behavior of these devices under high gate voltage conditions, it is necessary to determine the position-dependent quasi-Fermi levels. This requires solving the current continuity equations together with the Schrödinger and Poisson’s Equations [15].

In order to obtain the quasi-Fermi levels for calculating the carrier concentration, the continuity equation for the electron and hole currents must be solved, which is given in one dimension and steady state conditions:

45 $$ \frac{\partial n}{{\partial t}} = \frac{1}{q}\nabla \cdot J_{n} + \left( {G_{n} - R_{n} } \right) $$

46 $$ \frac{\partial p}{{\partial t}} = - \frac{1}{q}\nabla \cdot J_{p} + \left( {G_{p} - R_{p} } \right) $$

In these equations, n and p represent the concentrations of electrons and holes, respectively, while ${J}_{n}$ and ${J}_{p}$ denote the current densities for electrons and holes. The generation rates for electrons and holes are given by ${G}_{n}$ and ${G}_{p}$, while ${R}_{n}$ and ${R}_{p}$ represent their corresponding recombination rates. Additionally, q refers to the magnitude of the electron charge. Equations (45) and (46) offer a clear description of how carriers accumulate or deplete within a localized area. A more detailed discussion of the electron and hole current densities, ${J}_{n}$ and ${J}_{p}$, will be provided in the context of the transport equations.

The generation and recombination of carriers describe the mechanisms through which a semiconductor material seeks to restore electrical equilibrium after a disturbance. When the carrier concentration deviates from its equilibrium state due to excitation, a net recombination process is initiated to bring the system back to equilibrium. This phenomenon is commonly observed in crystals with traps or defects [69]. The steady-state equilibrium equations for electron and hole concentrations, ${\mathrm{n}}_{0}$ and ${\mathrm{p}}_{0}$, as well as the intrinsic concentration ${\mathrm{n}}_{\mathrm{i}}$, are expressed as follows:

47 $$ n_{0} p_{0} = n_{i}^{2} $$

The generation and recombination processes can be categorized into seven main types: phonon transitions, photon transitions, Auger recombination, Langevin recombination, Surface recombination, impact ionization, and band-to-band tunneling. Shockley, Read, and Hall proposed that phonon transitions occur in the presence of traps or defects whose energy levels are located within the semiconductor bandgap, and explained the nature of this transition as a two-step process [55, 163]. The SRH model is widely employed in HEMT simulations to accurately capture the complex carrier dynamics and recombination mechanisms within the device, [46, 57, 60, 68, 72, 108, 182, 194]. The SRH recombination rate is as follow:

48 $$ R_{SRH} = \frac{{np - n_{ie}^{2} }}{{\tau_{p} \left[ {n + n_{ie} exp\left( {\frac{{E_{trap} }}{{kT_{L} }}} \right)} \right] + \tau_{n} \left[ {p + n_{ie} exp\left( { - \frac{{E_{trap} }}{{kT_{L} }}} \right)} \right]}} $$

where ${\tau }_{n}$ and ${\tau }_{p}$ are the electron and hole lifetimes, ${\mathrm{n}}_{\mathrm{ie}}$ is the effective intrinsic concentration and ${T}_{L}$ is the lattice temperature. ${E}_{trap}$ is the difference between the trap level and the intrinsic Fermi level. ${\tau }_{n}$ and ${\tau }_{p}$ are usually constant or temperature-dependent functions as follows:

49 $$ \tau_{n} = N_{T0} \cdot \left( {\frac{{T_{L} }}{300K}} \right)^{{T_{L.N} }} $$

50 $$ \tau_{p} = P_{T0} \cdot \left( {\frac{{T_{L} }}{300K}} \right)^{{T_{L.P} }} $$

where ${\mathrm{N}}_{\mathrm{T0}}$ and ${\mathrm{P}}_{\mathrm{T0}}$ denote the electron and hole lifetimes, respectively, at a standard temperature of 300 K. ${\mathrm{T}}_{\mathrm{L.N}}$ and ${\mathrm{T}}_{\mathrm{L.P}}$ represent the index of material change with temperature.

Auger recombination occurs through a three-particle transition in which a conduction band electron and a valence band hole recombine and the excess energy is transferred to a third free electron or hole [58]. The general model of Auger recombination is as follows [82]:

51 $$ R_{Auger} = C_{n} n\left( {np - n_{ie}^{2} } \right) + C_{p} p\left( {np - n_{ie}^{2} } \right) $$

where n and p are the electron and hole concentration, and ${\mathrm{n}}_{{{\mathrm{ie}}}}$ is the effective intrinsic concentration. ${\mathrm{C}}_{{\mathrm{n}}}$ and ${\mathrm{C}}_{{\mathrm{p}}}$ are constants that define parameters related to doping concentration in the standard Auger model, simplifying the simulation of the Auger process in narrow bandgap semiconductors. This concentration-dependent model is a simplified version of the Beattie and White model [13]. The coefficients are given as follows:

52 $$ C_{n} = \frac{{N_{Aug} }}{{1 + N_{Aug.k} \cdot n}} $$

53 $$ C_{p} = \frac{{P_{Aug} }}{{1 + P_{Aug.k} \cdot p}} $$

where ${\mathrm{N}}_{{{\mathrm{Aug}}{\mathrm{.k}}}}$ and ${\mathrm{P}}_{{{\mathrm{Aug}}{\mathrm{.k}}}}$ are both zero by default. Both ${\mathrm{C}}_{{\mathrm{n}}}$ and ${\mathrm{C}}_{{\mathrm{p}}}$. default to the constant values given by ${\mathrm{N}}_{{{\mathrm{Aug}}}}$ and ${\mathrm{P}}_{{{\mathrm{Aug}}}}$. The Auger recombination coefficient for AlGaN is reported to be $2.3 \times 10^{ - 30} cm^{6} s^{ - 1}$ [133].

Impact ionization occurs in thspace-charge region under a high reverse bias. When free carriers are accelerated to energies sufficient to generate additional free electrons through collisions with crystal atoms, the resulting electron-hole pair generation near the gate significantly increases the drain current, ultimately leading to device avalanche breakdown [119]. The generation rate of electron/hole pair generated by the impact ionization process is expressed as follows:

54 $$ G_{I} = \alpha_{n} \left( {\left| {E_{nI} } \right|} \right)\frac{{\left| {J_{n} } \right|}}{q} + \alpha_{p} \left( {\left| {E_{pI} } \right|} \right)\frac{{\left| {J_{p} } \right|}}{q} $$

where ${\upalpha }_{{\mathrm{n}}} \left( {\left| {{\mathrm{E}}_{{{\mathrm{nI}}}} } \right|} \right)$ and ${\upalpha }_{{\mathrm{p}}} \left( {\left| {{\mathrm{E}}_{{{\mathrm{pI}}}} } \right|} \right)$ are the ionization coefficient for electrons and holes. ${\mathrm{J}}_{{\mathrm{n}}}$ and ${\mathrm{J}}_{{\mathrm{p}}}$ are the electron and hole current density. The ionization coefficient, which depends on the electric field, represents the number of carrier pairs generated per unit distance traveled by the carriers. The impact ionization model is employed to simulate effects like substrate current and device breakdown caused by impact ionization.

Based on the classical Chynoweth model, Selberherr introduced an impact ionization formula that has become a standard method for simulating semiconductor devices (except for applications in the deep submicron range) [30, 159]. This model is very suitable for use with the drift-diffusion transport framework. The mathematical expression describing the ionization rate in the model is as follows:

55 $$ a_{n} = A_{n} exp\left[ { - \left( {\frac{{B_{n} }}{{\left| {E_{nI} } \right|}}} \right)^{{N_{\beta } }} } \right] $$

56 $$ a_{p} = A_{p} exp\left[ { - \left( {\frac{{B_{p} }}{{\left| {E_{pI} } \right|}}} \right)^{{P_{\beta } }} } \right] $$

where ${\mathrm{E}}_{{{\mathrm{nI}}}}$ and ${\mathrm{E}}_{{{\mathrm{pI}}}}$ are the components of the electric field in the direction of electron and hole current flow. ${\mathrm{A}}_{{\mathrm{n}}}$ and ${\mathrm{A}}_{{\mathrm{p}}}$, ${\mathrm{B}}_{{\mathrm{n}}}$ and ${\mathrm{B}}_{{\mathrm{p}}}$ are the temperature and electric field dependence coefficients [111]. Their values depend on the relationship between $\left| {{\mathrm{E}}_{{{\mathrm{nI}}}} } \right|$, $\left| {{\mathrm{E}}_{{{\mathrm{pI}}}} } \right|$, and the threshold electric field. When $\left| {{\mathrm{E}}_{{{\mathrm{nI}}}} } \right|$ and $\left| {{\mathrm{E}}_{{{\mathrm{pI}}}} } \right|$ exceed the threshold electric field, ${\mathrm{A}}_{{\mathrm{n}}}$, ${\mathrm{A}}_{{\mathrm{p}}}$, ${\mathrm{B}}_{{\mathrm{n}}}$, and ${\mathrm{B}}_{{\mathrm{p}}}$ correspond to AN1, AP1, BN1, and BP1. Otherwise, they correspond to AN2, AP2, BN2, and BP2. For nitrides, these parameter pairs are typically equal. For the structure in Fig. 11, the default values for AN1 and AN2 are $2.52 \times 10^{8}$cm^−1^, AP1 and AP2 are $5.37 \times 10^{6}$ cm^−1^, BN1 and BN2 are $3.41 \times 10^{7}$ V/cm, BP1 and BP2 are $1.96 \times 10^{7}$ V/cm. ${\mathrm{N}}_{{\upbeta }}$ and ${\mathrm{P}}_{{\upbeta }}$ are usually 1. For other materials such as silicon, the default values of AP1 and AP2, BP1 and BP2 are different.

### Energy balance transport equations

The current density equation and charge transport model are derived from approximations and simplifications of the Boltzmann transport equation. Originally introduced by Ludwig Boltzmann in 1872 to describe dilute gases at the kinetic level, the equation has since been widely applied to various fields involving dilute carrier-mediated transport [24]. As the most basic method of charge transfer, the drift diffusion model depends only on φ, n and p without introducing any other independent variables. While the drift-diffusion model is applicable to most devices, it becomes less suitable for devices with smaller feature sizes. The carrier continuity equation is derived from the Boltzmann transport equation as a balance equation for carrier density [96, 159]. Under simplified assumptions, this balance equation can be transformed into the drift-diffusion model for current densities ${J}_{n}$ and ${J}_{p}$. The equations are expressed as follows:

57 $$ J_{n} = - qn\mu_{n} \nabla \phi_{fn} $$

58 $$ J_{p} = - qp\mu_{p} \nabla \phi_{fp} $$

where $\varphi_{{{\mathrm{fn}}}}$ and $\varphi_{{{\mathrm{fp}}}}$. are the quasi-Fermi potentials, ${\upmu }_{{\mathrm{n}}}$ and ${\upmu }_{{\mathrm{p}}}$ are the electron and hole mobilities. For non-degenerate semiconductors, Maxwell-Boltzmann statistics are used to approximate the relationship between carrier concentration and the quasi-Fermi potential [52, 193]. By reversing this relationship and referencing Eqs. (57) and (58), ${\mathrm{J}}_{{\mathrm{n}}}$ and ${\mathrm{J}}_{{\mathrm{p}}}$ can be expressed as:

59 $$ J_{n} = - qn\mu_{n} \nabla \left[ {\psi + \frac{{kT_{L} }}{q}\ln \left( {n_{ie} } \right)} \right] + kT_{L} \mu_{n} \nabla n $$

60 $$ J_{p} = - qp\mu_{p} \nabla \left[ {\psi - \frac{{kT_{L} }}{q}\ln \left( {n_{ie} } \right)} \right] - kT_{L} \mu_{p} \nabla p $$

where ${\mathrm{n}}_{{{\mathrm{ie}}}}$ is the effective intrinsic concentration, and $\psi$ is the electrostatic potential. Equations (59) and (60) are simplified using the Einstein relations to derive expressions involving carrier mobility and to incorporate the effective electric field. This results in the conventional drift-diffusion form of the current density equation, given as follows:

61 $$ J_{n} = qn\mu_{n} E_{n} + qD_{n} \nabla n $$

62 $$ J_{p} = qp\mu_{p} E_{p} - qD_{p} \nabla p $$

63 $$ D_{n} = \frac{{kT_{L} }}{q}\mu_{n} $$

64 $$ D_{p} = \frac{{kT_{L} }}{q}\mu_{p} $$

65 $$ E_{n} = - \nabla \left[ {\psi + \frac{{kT_{L} }}{q}\ln \left( {n_{ie} } \right)} \right] $$

66 $$ E_{p} = - \nabla \left[ {\psi - \frac{{kT_{L} }}{q}\ln \left( {n_{ie} } \right)} \right] $$

where ${\mathrm{E}}_{\mathrm{n}}$ and ${\mathrm{E}}_{\mathrm{p}}$ are the effective electric field. ${\mathrm{D}}_{\mathrm{n}}$ and ${\mathrm{D}}_{\mathrm{p}}$ are in the form after the Einstein relationship. The term with ${\mathrm{E}}_{\mathrm{n}}$ and ${\mathrm{E}}_{\mathrm{p}}$ on the right side of Eqs. (61) and (62) account for the drift of carriers under the influence of the electric field, while the other term represents carrier diffusion due to the carrier density gradient.

In transport equations, the cumulative effect of microscopic phenomena significantly influences the macroscopic mobility coefficients, ${\mu }_{n}$ and ${\mu }_{p}$. Examples of these phenomena include carrier interactions with lattice vibrations and scattering caused by impurity ions and defects. Mobility is typically a function of several factors, such as the local electric field, crystal temperature, and doping concentration. Mobility modeling generally distinguishes between low-field and high-field behaviors. Under low electric fields, carriers are nearly in equilibrium with the lattice, and mobility reflects a characteristic low-field value. In contrast, at high electric fields, the average drift velocity deviates from linear proportionality with the electric field strength. Instead, its growth rate diminishes and eventually saturates at a constant value. At high energy levels, impurity scattering has minimal impact on carriers, rendering mobility predominantly dependent on lattice temperature. The default low-field mobility model in Victory Device is a temperature-dependent low-field mobility model that conforms to the power law, and the expression is as follows:

67 $$ \mu_{n0} = \mu_{n} \cdot \left( {\frac{{T_{L} }}{300K}} \right)^{{ - T_{\mu .n} }} $$

68 $$ \mu_{p0} = \mu_{p} \cdot \left( {\frac{{T_{L} }}{300K}} \right)^{{ - T_{\mu .p} }} $$

where ${\upmu }_{{\mathrm{n}}}$ and ${\upmu }_{{\mathrm{p}}}$ represent the low-field electron and hole mobilities, respectively, at a reference temperature, typically 300 K. ${\mathrm{T}}_{{{\upmu }{\mathrm{.n}}}}$ and ${\mathrm{T}}_{{{\upmu }{\mathrm{.p}}}}$ are the temperature coefficients that determine how the electron and hole mobilities vary with lattice temperature. For III-V compound semiconductors with compositional variations, such as AlGaN, the mobility values must be recalculated to account for the specific material composition. This involves incorporating the effects of alloy scattering, strain, and other compositional-dependent factors into the mobility model.

### Lattice heat flow equations

From the analysis of the carrier continuity equation and the drift-diffusion model, lattice temperature is influenced by temperature-dependent parameters, as well as generation, recombination, and resistance effects. To simulate these effects, the lattice heat flow equation must be solved alongside the Poisson equation, continuity equation, and transport equation. The lattice heat flow equation representing the local area is as follows [49]:

69 $$ C\frac{{\partial T_{L} }}{\partial t} = \nabla \cdot \left( {\kappa \nabla T_{L} } \right) + H $$

where ${\mathrm{C}}$ is the heat capacity per unit volume, ${\upkappa }$ is the thermal conductivity, ${\mathrm{H}}$ is the heat generation rate, and ${\mathrm{T}}_{{\mathrm{L}}}$ is the local lattice temperature. The volumetric heat capacity model expresses the heat capacity as a function of the lattice temperature. The volume heat capacity model represents heat capacity as a function of lattice temperature. When the barrier layer material is AlGaN, a composition-dependent model should be used [138], expressed as follows:

70 $$ C = \rho \left\{ {c_{300} + c_{1} \left[ {\frac{{\left( {\frac{{T_{L} }}{300K}} \right)^{\beta } - 1}}{{\left( {\frac{{T_{L} }}{300K}} \right)^{\beta } + \left( {\frac{{c_{1} }}{{c_{300} }}} \right)}}} \right]} \right\} $$

where ${\mathrm{c}}_{{{300}}}$. is the value for the specific heat at 300 K [173]. $\rho$ is the mass density of the material, ${\mathrm{c}}_{{1}}$ is the difference between the specific heat, and $\beta$ is an additional material-dependent parameter. Similarly, for the thermal conductivity of GaN materials, the EU-funded ALMA project (Full-Scale Predictive Design of Material Architectures for Thermal Management) calculated thermal conductivities for various materials across discrete temperatures ranging from 100 K to their melting points [3]. This project utilized the phonon Boltzmann transport equation (BTE) to derive the thermal conductivity [34, 111, 190]. The phonon BTE typically employs the relaxation time approximation, enabling the simulation of non-Fourier heat conduction problems and accurately capturing the thermal response caused by phonon collisions. The scattering mechanisms can be effectively modeled using the phonon BTE. Its governing equation is expressed as [111, 148]:

71 $$ \frac{\partial f}{{\partial t}} + v \cdot \nabla f = - \frac{{f - f^{eq} }}{\tau } $$

From left to right, it includes non-equibrium terms, transport terms and collision terms. Where f represents the distribution function of the rope, which is related to space, time, frequency and polarization. ${\mathrm{v}}$. represents the group velocity of phonons, ${\uptau }$ is the relaxation time, and ${\mathrm{f}}^{{{\mathrm{eq}}}}$ is the equilibrium distribution function of phonons, which follows the Bose-Einstein distribution [195].

The heat generation rate influences the lattice temperature ${T}_{L}$, which in turn affects the current densities ${J}_{n}$ and ${J}_{p}$ in t drift-diffusion equation. Assuming steady-state conditions, where the divergence of current is replaced by net recombination, the heat generation rate $H$ is as follows:

72 $$ \begin{aligned} H = & \,\left( {\frac{{J_{n} \cdot J_{n} }}{{qn\mu_{n} }} + \frac{{J_{p} \cdot J_{p} }}{{qp\mu_{p} }}} \right) - T_{L} \left( {J_{n} \cdot \nabla P_{n} + J_{p} \cdot \nabla P_{p} } \right) \\ & \, + q\left( {R - G} \right)\left[ {\left( {\phi_{fp} - \phi_{fn} } \right) + T_{L} \left( {P_{p} - P_{n} } \right)} \right] \\ \end{aligned} $$

73 $$ H_{J} = \left( {\frac{{J_{n} \cdot J_{n} }}{{qn\mu_{n} }} + \frac{{J_{p} \cdot J_{p} }}{{qp\mu_{p} }}} \right) $$

74 $$ H_{PT} = - T_{L} \left( {J_{n} \cdot \nabla P_{n} + J_{p} \cdot \nabla P_{p} } \right) $$

75 $$ H_{GR} = q\left( {R - G} \right)\left[ {\left( {\phi_{fp} - \phi_{fn} } \right) + T_{L} \left( {P_{p} - P_{n} } \right)} \right] $$

where R and G are the recombination and generation rates, ${\mathrm{P}}_{\mathrm{n}}$ and ${\mathrm{P}}_{\mathrm{p}}$ are the electron and hole thermoelectric power. ${\mathrm{P}}_{\mathrm{n}}$ and ${\mathrm{P}}_{\mathrm{p}}$ can be derived from the relaxation time approximation of the Boltzmann transport equation. Equations (73), (74), and (75) represent Joule heating, the combined Peltier and Joule-Thomson effects, and heating and cooling from recombination and generation, respectively [152, 20, 36].

When the bias voltage is raised, the internal power density of GaN HEMT increases, and the concentration of 2DEG at the GaN/AlGaN heterojunction interface rises. This causes more high-energy electrons to collide with the lattice, which raises the local temperature, decreases carrier mobility, and increases electron phonon scattering—all of which are referred to as self-heating effects [23, 27, 28, 35]. These problems raise the probability of thermal degradation and impair overall device performance by introducing thermal stress in addition to deteriorating the transistor’s static I-V characteristics [22, 59, 86]. Previously, the majority of research on non-Fourier heat transfer processes employed macroscopic models, such as the single-phase lag (SPL) model [131], the double-phase lag (DPL) model [132, 151], and the ballistic diffusion equation (BDE) [168]. While these approaches offer computational efficiency and are useful in certain contexts, they are often based on phenomenological assumptions and fail to capture critical microscopic details of heat transfer. This limitation can significantly compromise the accuracy of their predictions, particularly for systems requiring a more rigorous physical foundation. In contrast, Fourier-based heat transfer models remain a reliable and robust choice for capturing the essential physics of bulk materials and accurately describing heat conduction processes under diffusive conditions [149].

## Advanced physical model analysis

In Sect. 3, the joint solution of Poisson’s equation, the carrier continuity equation, the transport equation, and the lattice heat flow equation were used to model the fundamental HEMT structure, providing numerical results for key characteristics such as output and transfer characteristics, as well as breakdown voltage. However, achieving accurate simulations of HEMT power devices requires selecting appropriate physical models to account for critical transport and breakdown mechanisms. This chapter explores advanced models implemented in TCAD simulations to enhance accuracy across different material systems and device structures, including specialized mobility models that incorporate velocity saturation, impurity scattering, and field-dependent effects, as well as recombination models that consider temperature and carrier concentration dependence. Additionally, various impact ionization models are analyzed to assess their effectiveness in predicting breakdown voltage and avalanche effects. By examining these advanced models, this chapter provides insights into optimizing TCAD simulations for more realistic and reliable predictions of HEMT performance.

### Mobility models

#### Field-dependent mobility model (FLDMOB)

**Fig. 12:**
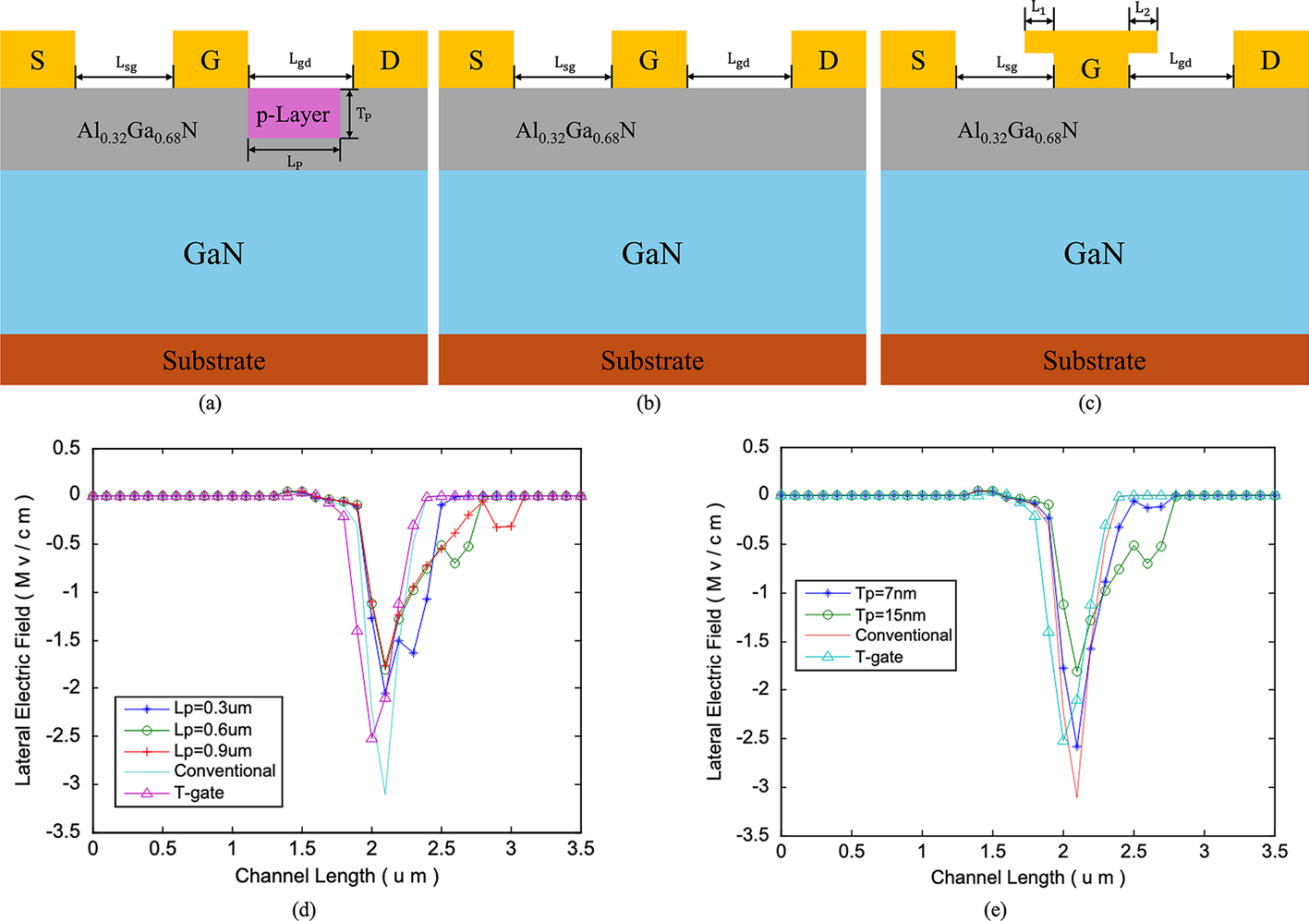
Cross-section of **a** p-layer HEMT, **b** conventional HEMT and **c** T-gate HEMT structures. And lateral electric field distribution diagrams of **d** different ${L}_{P}$ at fixed ${T}_{P}= 15 nm$ and **e** different ${T}_{P}$ at fixed ${L}_{P}=0.6 \mu m$. Reprinted with permission from ref. [150]. Copyright 2013 Elsevier

Razavi et al. proposed an AlGaN/GaN HEMT (PL-HEMT) incorporating a p-layer in the barrier [150]. This design demonstrated significant improvements in breakdown voltage, suppression of short-channel effects, reduction in gate-drain capacitance, lower subthreshold slope, and decreased output conductance. Figure 12 (a), (b) and (c) compares three HEMT structures: p-Layer HEMT, conventional HEMT, and T-gate HEMT. In this design, the Al composition of the barrier layer is 0.32. The p-layer in the barrier consists of heavily doped p-type AlGaN, with a doping concentration equal to that of the barrier layer. The simulation incorporates the SRH model, the CONMOB model for mobility, the FLDMOB model for parallel electric field dependence, and the Fermi-Dirac model for carrier distribution.

In the lateral electric field analysis of different HEMT structures, the gate-source voltage ${V}_{gs}$ and drain-source voltage ${V}_{ds}$ are set to − 4 V and 80 V, respectively, as shown in Fig. 12 (d) and (e). Among the analyzed structures, the conventional HEMT exhibits an electric field strength of 3 MV/cm at the gate edge, which is very close to the theoretical critical electric field of GaN. This suggests that the device is on the verge of breakdown. For the other HEMT structures, the minimum electric field at the gate edge also reaches approximately 1.6 MV/cm, indicating that the electric field strength in these devices is significant, leading to carrier acceleration toward velocity saturation. This phenomenon can be attributed to the reduction in effective carrier mobility, as the drift velocity is determined by the product of carrier mobility and the electric field component along the current flow direction. To characterize this behavior, Caughey and Thomas proposed a field-dependent mobility model (FLDMOB), which provides a smooth transition between the low-field and high-field regions [19]. The default models are expressed as follows:

76 $$ \mu_{n} = \mu_{n0} \left[ {1 + \left( {\frac{{\mu_{n0} \left| {E_{\parallel } } \right|}}{{V_{satn} }}} \right)^{2} } \right]^{ - 1/2} $$

77 $$ \mu_{p} = \mu_{p0} \left[ {1 + \left( {\frac{{\mu_{p0} \left| {E_{\parallel } } \right|}}{{V_{satp} }}} \right)^{1} } \right]^{ - 1/1} $$

where $\left|{E}_{\parallel }\right|$ is the magnitude of the parallel electric field, ${\mu }_{n0}$ and ${\mu }_{p0}$ are still the low-field electron and hole mobilities. Additionally, the saturation velocities are, by default, governed by the temperature-dependent model proposed by Schwarz and Russe [158], which is described as follows:

78 $$ V_{satn} = \frac{{\alpha_{n.fld} }}{{1 + \theta_{n.fld} \cdot exp\left( {\frac{{T_{L} }}{{T_{n.fld} }}} \right)}} $$

79 $$ V_{satp} = \frac{{\alpha_{p.fld} }}{{1 + \theta_{p.fld} \cdot exp\left( {\frac{{T_{L} }}{{T_{p.fld} }}} \right)}} $$

where ${\mathrm{T}}_{{{\mathrm{n}}{\mathrm{.fld}}}}$ and ${\mathrm{T}}_{{{\mathrm{p}}{\mathrm{.fld}}}}$ are the temperature parameters with default value of 600 K for electrons and holes. ${\upalpha }_{{{\mathrm{n}}{\mathrm{.fld}}}}$ and ${\upalpha }_{{{\mathrm{p}}{\mathrm{.fld}}}}$ represent the theoretical saturation velocity of electron and hole in the GaN material under high electric field conditions. The predicted saturation velocity in the GaN channel ranges from $1.9 \times 10^{7}$ to $3 \times 10^{7}$ cm/s [79]. In simulations, the default value is set to $2.4 \times 10^{7}$ cm/s. Compared to the default temperature-dependent model, the FLDMOB model incorporates the effects of parallel electric fields and velocity saturation, providing a more accurate transition between low-field and high-field regions. This makes it well-suited for simulating carrier transport. Consequently, the FLDMOB model is appropriate for analyzing output characteristics, transfer characteristics, and breakdown voltage under high-voltage conditions. In such simulations, the corresponding electric field strength in the 2DEG channel should approximately reach the theoretical critical electric field.

**Fig. 13:**
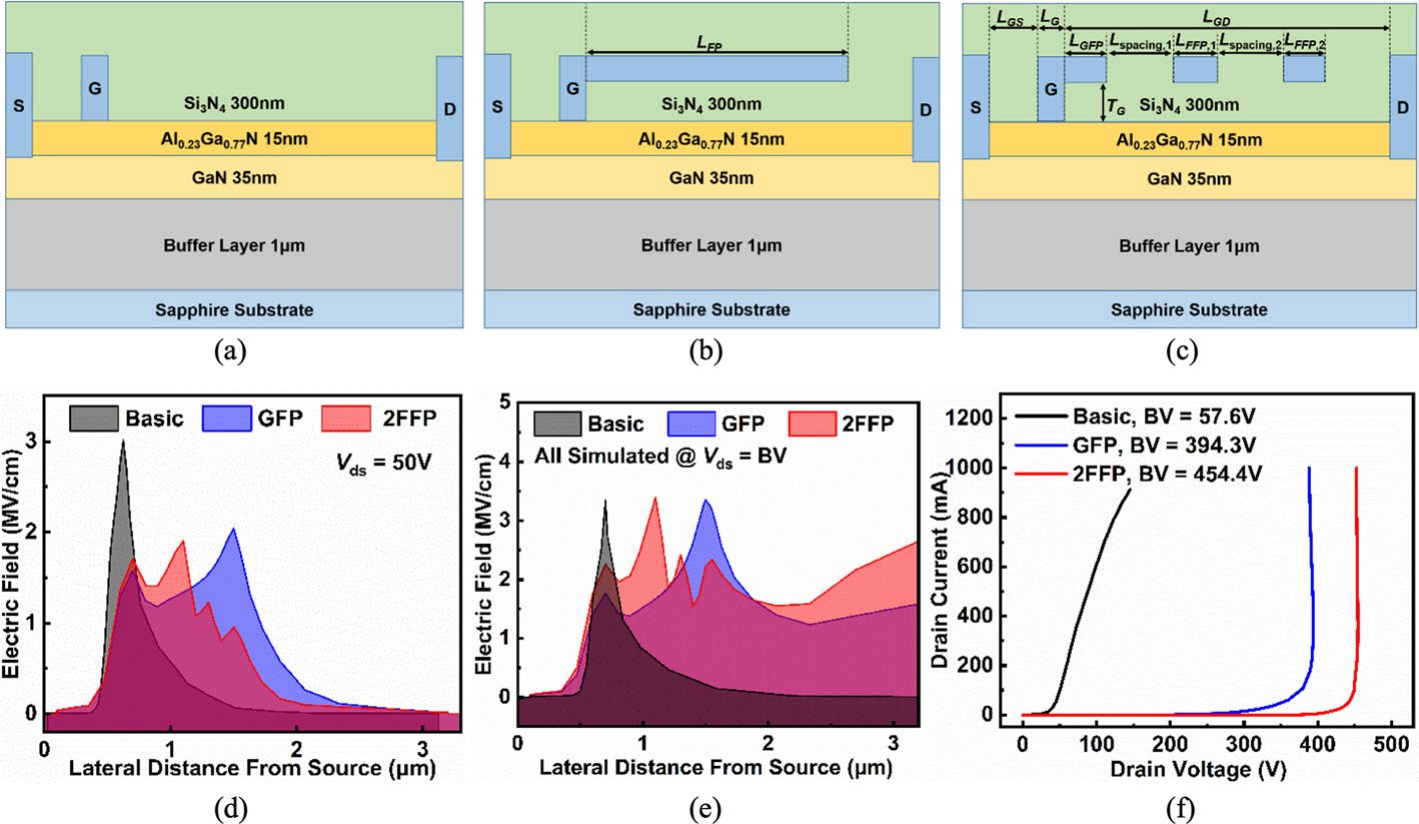
**a** Basic HEMT structure, **b** HEMT with a gate field plate, and **c** cross-section of a double floating field plate HEMT. **d** Electric field distribution in the 2DEG channel at ${V}_{d}=50V$, **e** electric field distribution in the 2DEG channel at breakdown voltage, and **f** comparison of breakdown voltages for the three HEMT structures [178]. Copyright 2023 MDPI

Wang et al. employed the FLDMOB model to enhance high-field mobility in breakdown voltage simulations of double floating gate field plate AlGaN/GaN HEMTs [178]. Figure 13(a), (b), and (c) illustrate the basic HEMT, gate field plate HEMT, and double floating field plate HEMT structures, respectively. Figure 13(d) presents the electric field distribution in the 2DEG channel of the three HEMTs at ${V}_{d}=50V$, where the electric field remains low, demonstrating the field plate’s effectiveness in improving field distribution. Figure 13(e) compares the electric field strength at breakdown voltage, showing that all three HEMTs reach approximately 3.3 MV/cm, aligning with GaN’s theoretical critical electric field. Additionally, the simulation accurately captures high-field effects at the gate edge and double floating field plate under extreme conditions. Figure 13(f) displays the simulated breakdown voltage of the three HEMTs, further validating the accuracy of the simulation results*.*

**Fig. 14:**
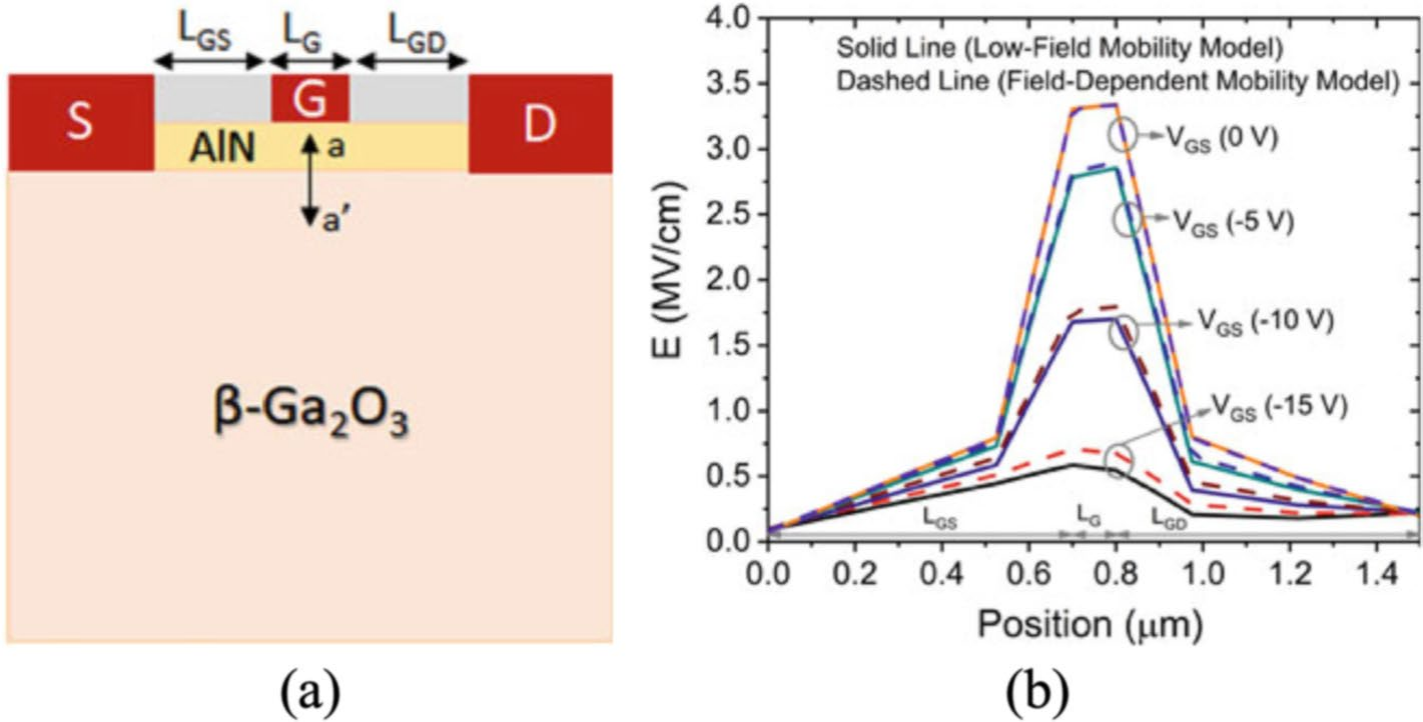
**a** Cross-sectional structure of AlN/B-Ga_2_O_3_ HEMT, and **b** electric field distribution under the gate simulated. using the low-field mobility model and the field-dependent mobility model at different ${\mathrm{V}}_{\mathrm{g}}$ with ${\mathrm{V}}_{\mathrm{d}}= \mathrm{3V}$. Reprinted under terms of the CC-BY license [164]. Copyright 2022, Singh et al., published by Springer Nature

Rajan et al. investigated the impact of buried gate size on electron mobility in AlN/β-Ga_2_O_3_ HEMTs using TCAD simulations [164]. The cross-section of the HEMT structure is illustrated in Fig. 14(a). Figure 14(b) presents the electric field distribution in the channel for different ${\mathrm{V}}_{{\mathrm{g}}}$, where the low-field mobility model and the FLDMOB model are represented by solid and dotted lines, respectively. The results show that at ${\mathrm{V}}_{{\mathrm{g}}} {\text{ = 0V}}$, the electric field distributions of both models align closely. However, as ${\mathrm{V}}_{{\mathrm{g}}}$ decreases to -15V, the disparity between the two models increases, with the FLDMOB model consistently exhibiting higher values in high-field regions. This indicates that the FLDMOB model provides a more accurate representation of carrier mobility in high electric field conditions.

#### Albrecht low-field mobility model (ALBRCT)

As for other suitable mobility models, the Albrecht low-field mobility model proposed by Albrecht et al. is designed for group III nitrides [4]. This model consists of three different components, which are combined using the Matthiessen rule and expressed by Eqs. (80) and (81). Where ${\mu }_{n0}$ and ${\mu }_{p0}$ are the total low field mobilities for electrons and holes, and N is the local ionized impurity concentration. Compared to the temperature-dependent mobility model described in Eqs. (67) and (68), the Albrecht model incorporates additional parameters, such as ${N}_{n0}$, ${T}_{n0}$, ${T}_{n1}$, AN, BN, and CN. Among these, the parameters AN, BN, and CN are coefficients derived using Matthiessen’s rule. These parameters are determined by fitting results from Monte Carlo simulations. The default values for these parameters are listed in Table 5.

80 $$ \frac{1}{{\mu_{n0} }} = \frac{{A_{N} \cdot N}}{{N_{n0} }}\left( {\frac{{T_{L} }}{{T_{n0} }}} \right)^{ - 3/2} \ln \left( {1 + 3\left( {\frac{{T_{L} }}{{T_{n0} }}} \right)^{2} \left( {\frac{N}{{N_{n0} }}} \right)^{ - 3/2} } \right) + B_{N} \left( {\frac{{T_{L} }}{{T_{n0} }}} \right)^{3/2} + \frac{{C_{N} }}{{exp\left( {T_{n1} /T_{L} } \right) - 1}} $$

81 $$ \frac{1}{{\mu_{p0} }} = \frac{{A_{P} \cdot P}}{{N_{p0} }}\left( {\frac{{T_{L} }}{{T_{p0} }}} \right)^{ - 3/2} \ln \left( {1 + 3\left( {\frac{{T_{L} }}{{T_{p0} }}} \right)^{2} \left( {\frac{P}{{N_{p0} }}} \right)^{ - 3/2} } \right) + B_{P} \left( {\frac{{T_{L} }}{{T_{n0} }}} \right)^{3/2} + \frac{{C_{P} }}{{exp\left( {T_{p1} /T_{L} } \right) - 1}} $$

**Table 5:** Default parameter values for the ALBRECHT model

Parameter	Value	Parameter	Value	Units
${A}_{N}$	$2.61\times {10}^{-4}$	${A}_{P}$	$2.61\times {10}^{-4}$	$\left( {V \cdot s} \right)/cm^{2}$
${B}_{N}$	$2.9\times {10}^{-4}$	${B}_{P}$	$2.9\times {10}^{-4}$	$\left( {V \cdot s} \right)/cm^{2}$
${C}_{N}$	$170\times {10}^{-4}$	${C}_{P}$	$170\times {10}^{-4}$	$\left( {V \cdot s} \right)/cm^{2}$
${N}_{n0}$	$1\times {10}^{17}$	${N}_{p0}$	$1\times {10}^{17}$	${cm}^{-3}$
${T}_{n0}$	300	${T}_{p0}$	300	K
${T}_{n1}$	1065	${T}_{p1}$	1065	K

**Fig. 15:**
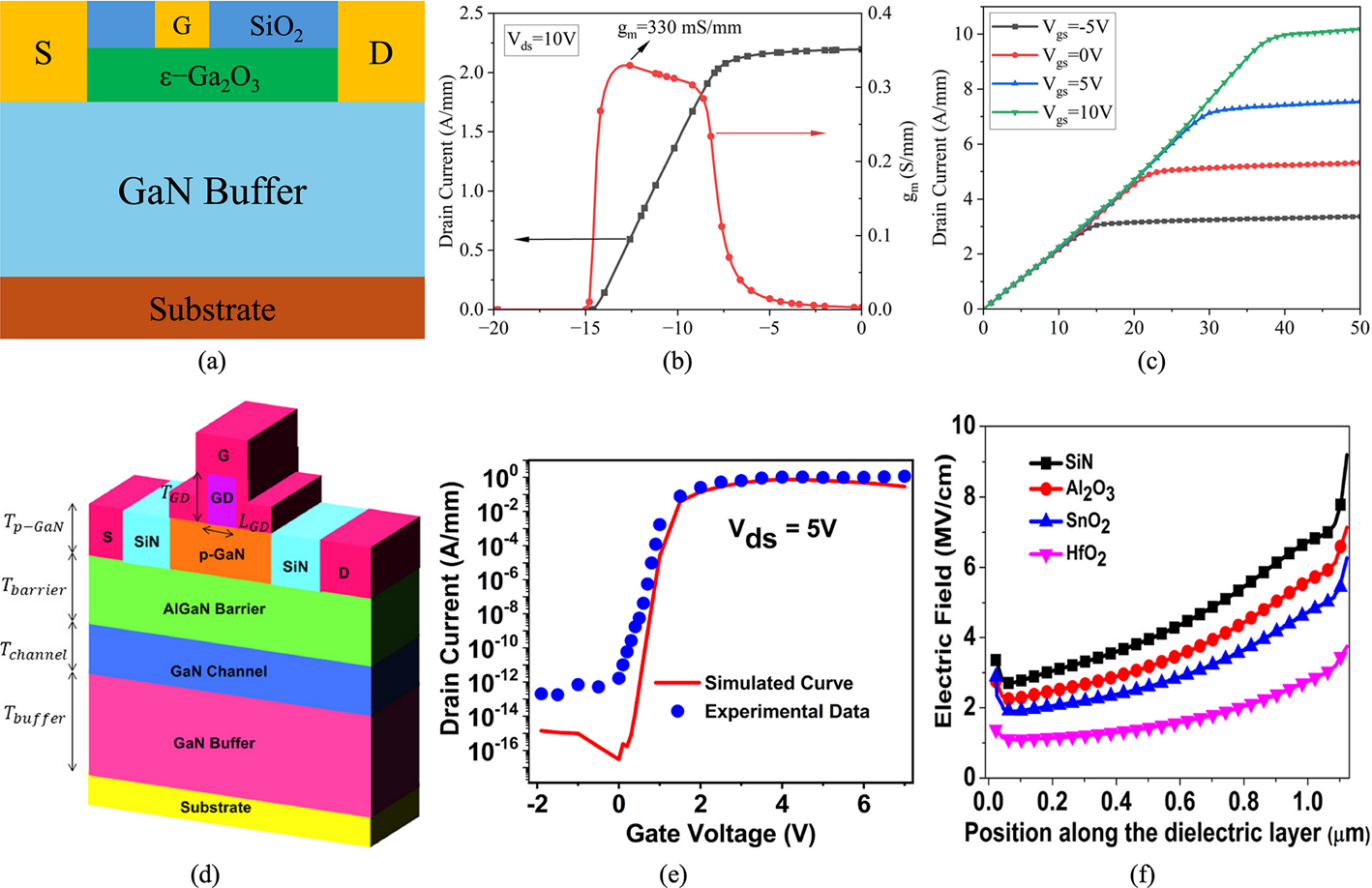
**a** Cross-sectional structure of the $\varepsilon -Ga2O3$/GaN HEMT, **b** transmission and **c** output characteristics. Reprinted under terms of the CC-BY license [144]. Copyright 2025, Qu et al., published by Springer Nature. **d** Cross-sectional structure of an omega-gate p-GaN MIS-HEMT, **e** transfer characteristics comparing experimental and simulated results, and **f** impact of different gate dielectric materials on the electric field distribution at breakdown voltage. Reprinted with permission from ref. [47]. Copyright 2024 Elsevier

Qu et al. investigated the effect of acceptor traps in the GaN buffer layer on current collapse in $\varepsilon -Ga2O3$/GaN HEMTs [144]. The HEMT structure is illustrated in Fig. 15(a). Using numerical simulation methods, they employed the Albrecht mobility model to accurately capture the relationship between carrier mobility, doping concentration, and lattice temperature. Fig. 15(b) and (c) depict the transfer and output characteristics of the $\varepsilon -Ga2O3$/GaN HEMT, respectively. The Id-Vg, transconductance, and Id-Vd characteristics confirm that the simulation effectively replicates the performance of the HEMT device. These results demonstrate that the Albrecht model accurately accounts for impurity scattering and electric field effects, ensuring precise carrier transport simulation under various operating conditions.

#### Gansat high-field mobility model (GANSAT)

The high-field mobility model is derived based on the low-field mobility model. Specifically, the Gansat high-field mobility model is a Monte Carlo data fit for electron mobility in bulk GaN materials. The mobility in this model is expressed as Eqs. (82) and (83) [38]:

82 $$ \mu_{n} = \frac{{\mu_{n0} + \left( {\frac{{V_{sat.n} }}{\left| E \right|}} \right)\left( {\frac{\left| E \right|}{{E_{CN} }}} \right)^{{N_{1n} }} }}{{1 + \left( {\frac{\left| E \right|}{{E_{CN} }}} \right)^{{N_{1n} }} + A_{N} \cdot \left( {\frac{\left| E \right|}{{E_{CN} }}} \right)^{{N_{2n} }} }} $$

83 $$ \mu_{p} = \frac{{\mu_{p0} + \left( {\frac{{V_{sat.p} }}{\left| E \right|}} \right)\left( {\frac{\left| E \right|}{{E_{CP} }}} \right)^{{N_{1p} }} }}{{1 + \left( {\frac{\left| E \right|}{{E_{CP} }}} \right)^{{N_{1p} }} + A_{P} \cdot \left( {\frac{\left| E \right|}{{E_{CP} }}} \right)^{{N_{2p} }} }} $$

where $\left|{\mathrm{E}}\right|$ is the magnitude of the electric field. The default electron mobility values for the GaN material parameters in Eqs. (82) and (83) are listed in Table 6. These parameters are extracted for both ternary compounds for the two bracketing cases of alloy scattering. The Albrecht and Gansat models are commonly used together in simulations of III-nitride-based HEMTs, with Albrecht modeling carrier mobility in the low-field region and Gansat accounting for mobility in the high-field region.

**Table 6:** Default values for the GANSAT model

Parameter	Value	Units
${A}_{N}$	6.1973	–
${V}_{sat.n}$	$1.9064\times {10}^{7}$	$\left( {{\mathrm{cm/s}}} \right)$
${N}_{n1}$	7.2044	–
${N}_{n2}$	0.7857	–
${E}_{CN}$	$220.8936\times {10}^{3}$	(v/cm)

**Fig. 16:**
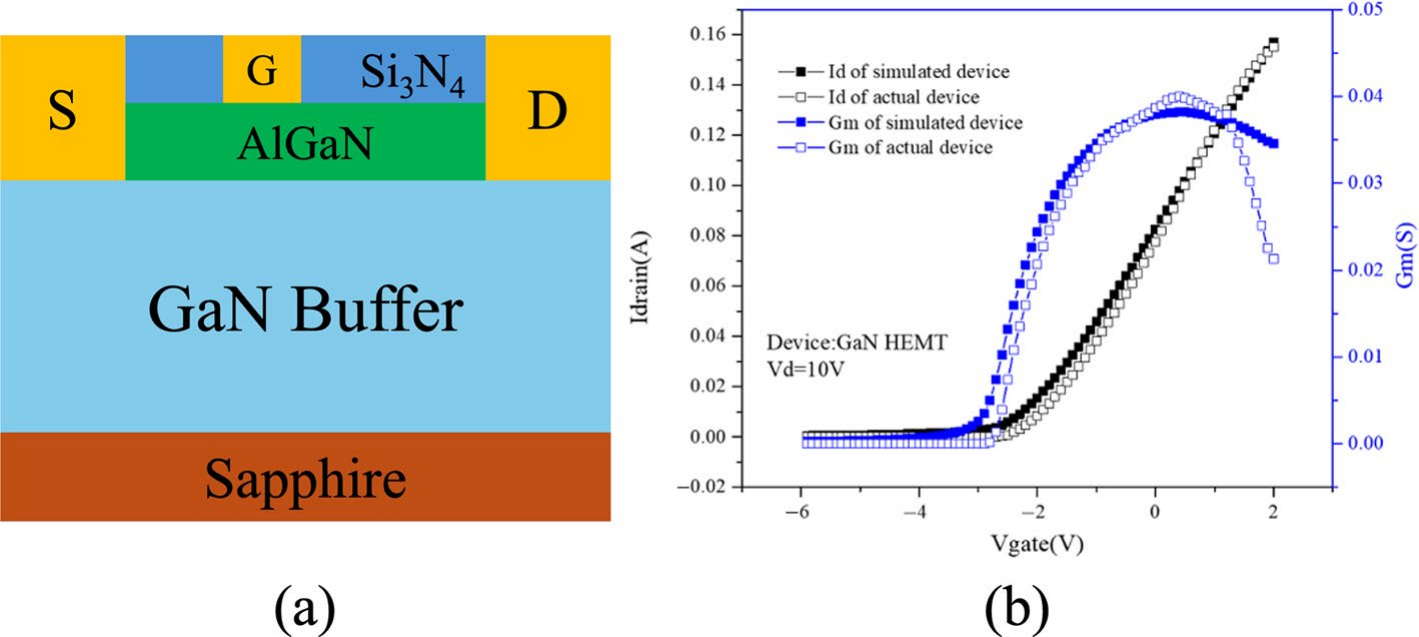
**a**Cross-sectional structure of GaN HEMT, and **b** transfer characteristics and transconductance results comparing simulation and experimental tests. Reprinted with permission from ref. [179]. Copyright 2023 MDPI

Garg et al. proposed an Omega-gate p-GaN MIS-HEMT structure and investigated its performance enhancement using TCAD simulations [47]. The HEMT structure is illustrated in Fig. 15(d), and the study employed the Gansat mobility model for nitrides to simulate carrier transport. Figure 15(e) compares the experimental and simulated transfer characteristics of the p-GaN HEMT, showing strong agreement and confirming the reliability of the simulation model [110]. Additionally, Fig. 15(f) presents the electric field distribution in HEMTs with different gate dielectric materials at breakdown voltage, reaching approximately 8 MV/cm. The ability of the simulation to reproduce such high field strength further validates the Gansat model’s accuracy in capturing high-field effects in HEMTs. Similarly, Wang et al. used TCAD simulations to analyze single-particle transient effects in GaN HEMTs [179]. The cross-sectional structure of the simulated GaN HEMT is shown in Fig. 16(a), where the study also employed the Gansat high-field mobility model. Figure 16(b) presents the transfer characteristics and transconductance curves of both simulated and experimental devices, demonstrating strong correlation and further validating the accuracy of the model*.*

In addition, Chen and Wang developed optimized parameters based on the GaNsat mobility model and the hole mobility model for GaN [25]. Their model refined the GaNsat mobility model parameters through Monte Carlo simulations conducted by Chen and Wang. Furthermore, they investigated the high-field carrier transport characteristics of bulk wurtzite GaN using the ensemble Monte Carlo (EMC) model, incorporating non-parabolic bands and various scattering mechanisms. The impact ionization process was carefully analyzed, demonstrating the potential for enhanced gain and reduced noise behavior in wurtzite GaN. Consequently, adopting this model instead of GaNsat for GaN materials provides more precise material parameter extraction.

#### Yamaguchi mobility model (YAMAGUCHI)

The Yamaguchi model is a comprehensive mobility model that accounts for doping concentration, inversion layer effects, and high-field effects [187]. However, it does not include temperature-dependent parameters and is only applicable when the lattice temperature is 300K. Initially developed for MOSFETs, this model has been successfully adapted to capture the mobility of the 2DEG in HEMTs. The Yamaguchi model considers the effects of doping, as well as both lateral and vertical electric fields on the current modeling mobility of HEMTs. By integrating dopant scattering, lateral, and transverse electric field scattering into a single model, the Yamaguchi model provides a comprehensive approach. Adjusting the transverse field and transverse field parameters, along with saturation velocity values, can enhance the model’s accuracy in fitting the measured parameters [127]. The Yamaguchi model is utilized to determine the low-field mobility influenced by doping concentration. For GaN materials, the low-field mobility is computed using the following equation:

84 $$ \mu_{n0} = 1400\left[ {1 + \frac{{N_{i} }}{{\frac{{N_{i} }}{350} + 3 \times 10^{16} }}} \right]^{{ - \frac{1}{2}}} $$

85 $$ \mu_{p0} = 480\left[ {1 + \frac{{N_{i} }}{{\frac{{N_{i} }}{81} + 4 \times 10^{16} }}} \right]^{{ - \frac{1}{2}}} $$

where ${\mathrm{N}}_{\mathrm{i}}$ is the net impurity concentration. The transverse electric field dependence for GaN is as follows:

86 $$ \mu_{s,n} = \mu_{n0} \left( {1 + 1.54 \times 10^{ - 5} E_{ \bot } } \right)^{{ - \frac{1}{2}}} $$

87 $$ \mu_{s,p} = \mu_{p0} \left( {1 + 5.35 \times 10^{ - 5} E_{ \bot } } \right)^{{ - \frac{1}{2}}} $$

where ${\mathrm{E}}_{ \bot }$ represents the transverse electric field. Thus, the final calculation of mobility concerning lateral electric fields is given by Eqs. (88) and (89). Where E is the lateral electric field. The zero-bias mobility data for electrons and holes were obtained through simulations of the two devices. The zero-bias electron mobility values for the AlGaN/GaN and AlGaN/AlN/GaN structures are ${\text{1100 cm}}^{{2}} {\mathrm{/V}}\cdot{\mathrm{s}}$ and ${\text{1240 cm}}^{{2}} {\mathrm{/V}}\cdot{\mathrm{s}}$, respectively. Similarly, the zero-bias hole mobility values for the AlGaN/GaN and AlGaN/AlN/GaN structures are $370 {\mathrm{cm}}^{2} /{\mathrm{V}}\cdot{\mathrm{s}}$ and $420\;{\mathrm{cm}}^{2} /{\mathrm{V}}\cdot{\mathrm{s}}$, respectively.

88 $$ \mu_{n} = \mu_{s,n} \left[ {1 + \left( {\frac{{\mu_{s,n} E}}{{4.9 \times 10^{6} }}} \right)^{2} \left( {8.8 + \frac{{\mu_{s,n} E}}{{4.9 \times 10^{6} }}} \right)^{ - 1} + \left( {\frac{{\mu_{s,n} E}}{{1.036 \times 10^{7} }}} \right)^{2} } \right]^{{ - \frac{1}{2}}} $$

89 $$ \mu_{p} = \mu_{s,p} \left[ {1 + \left( {\frac{{\mu_{s,p} E}}{{2.928 \times 10^{6} }}} \right)^{2} \left( {1.6 + \frac{{\mu_{s,p} E}}{{2.928 \times 10^{6} }}} \right)^{ - 1} + \left( {\frac{{\mu_{s,p} E}}{{1.2 \times 10^{7} }}} \right)^{2} } \right]^{{ - \frac{1}{2}}} $$

**Fig. 17:**
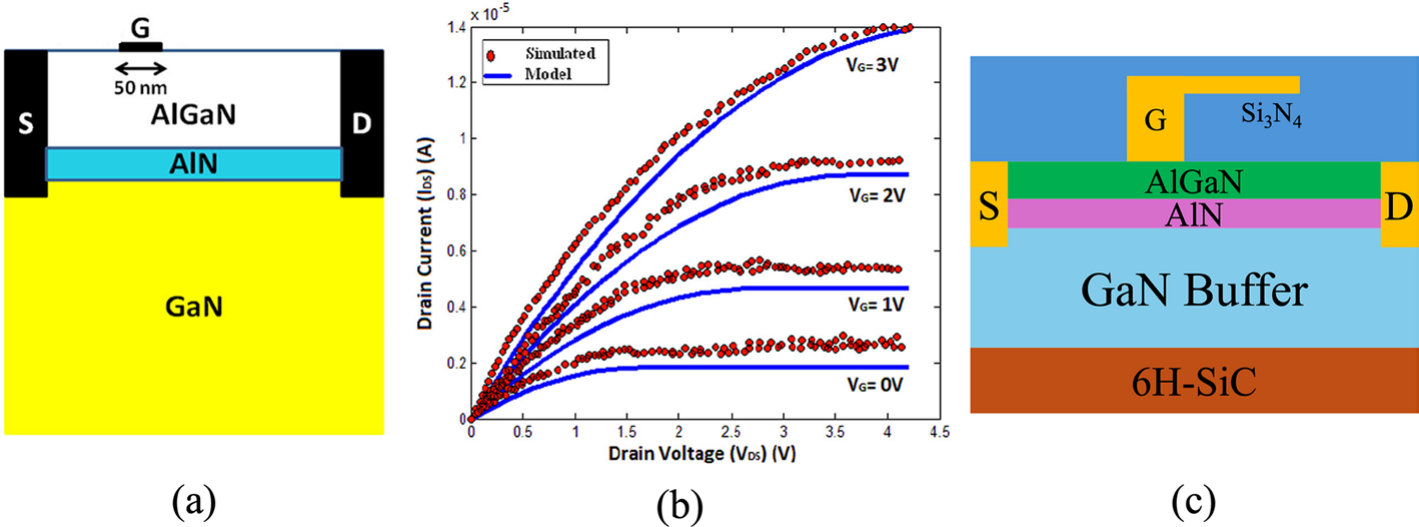
**a** Cross-sectional structure of an AlGaN/AlN/GaN HEMT, **b** output characteristic curves, and **c** cross-sectional structure of an AlGaN/AlN/GaN HEMT with gate field plate. Reprinted with permission from ref. [142]. Copyright 2016 Springer Nature

Prasad et al. conducted a comparative simulation study on AlGaN/GaN HEMTs without AlN spacers and AlGaN/AlN/GaN HEMTs with 5 nm AlN spacers [142]. Figure 17(a) illustrates the structure of the AlGaN/AlN/GaN HEMT. The Yamaguchi model was used in the simulation to calculate the low-field doping-dependent mobility, which was then combined with the effects of lateral and vertical electric fields to determine carrier mobility. Figure 17(b) compares the simulation results with those of the numerical model, showing small error between the two curves, confirming the accuracy of the model. In addition, Prasad et al. applied field plate technology to AlGaN/AlN/GaN HEMT simulations to improve the breakdown voltage [143]. The comparison of output characteristics between simulation results and experimental data is shown in Fig. 17(c). The strong agreement between simulation and experimental results confirms that the Yamaguchi model provides reliable predictions of mobility behavior in both the channel and ohmic/contact regions [187]. Furthermore, the model calculates a channel charge mobility of ${\text{1407 cm}}^{{2}} {\mathrm{/V}}\cdot{\mathrm{s}}$ at 300 K under zero bias, which decreases to ${\text{227 cm}}^{{2}} {\mathrm{/V}}\cdot{\mathrm{s}}$ when the temperature increases to 600 K*.*

#### Mobility models with carrier-carrier scattering effect

The Brooks-Herring (BH) model applies the Matthiessen inverse summation rule to incorporate carrier-carrier scattering (CCS) effects into low-field mobility. The model evaluates the contribution of CCS to mobility using the following expression:

90 $$ \mu = \frac{{1.56 \times 10^{21} \cdot \left( {\frac{{T_{L} }}{300K}} \right)^{3/2} }}{{\sqrt {np} \cdot \phi \left( \eta \right)}} $$

91 $$ \phi \left( \eta \right) = \ln \left( {1 + \eta } \right) - \frac{\eta }{1 + \eta } $$

92 $$ \eta = \frac{{7.63 \times 10^{21} \cdot \left( {\frac{{T_{L} }}{300K}} \right)^{2} }}{{N_{C} \cdot F_{ - 1/2} \left( {\frac{n}{{N_{C} }}} \right) + N_{V} \cdot F_{ - 1/2} \left( {\frac{p}{{N_{V} }}} \right)}} $$

where ${\mathrm{N}}_{{\mathrm{C}}}$ and ${\mathrm{N}}_{{\mathrm{V}}}$ represent the effective density of states for the conduction band and valence band, respectively. ${\mathrm{F}}_{{{ - 1}/{2}}}$ is the Fermi-Dirac integral of order ${ - 1}/{2}$. The model then combines $\mu$ and the concentration-dependent mobility to determine the low-field mobility:

93 $$ \frac{1}{{\mu_{n,p} }} = \frac{1}{{\mu_{n0,p0} }} + \frac{1}{\mu } $$

Compared with the Brooks-Herring model, the Conwell-Weisskopf (CW) model covers a larger compensation ratio range of ionized impurity concentrations [32]. The carrier mobility expression of the CW model is as follows:

94 $$ \mu = \frac{{1.04 \times 10^{21} \cdot \left( {\frac{{T_{L} }}{300K}} \right)^{3/2} }}{{\sqrt {np} \cdot \ln \left[ {1 + \frac{{7.452 \times 10^{13} }}{{\left( {np} \right)^{1/3} }}\left( {\frac{{T_{L} }}{300K}} \right)^{2} } \right]}} $$

**Fig. 18:**
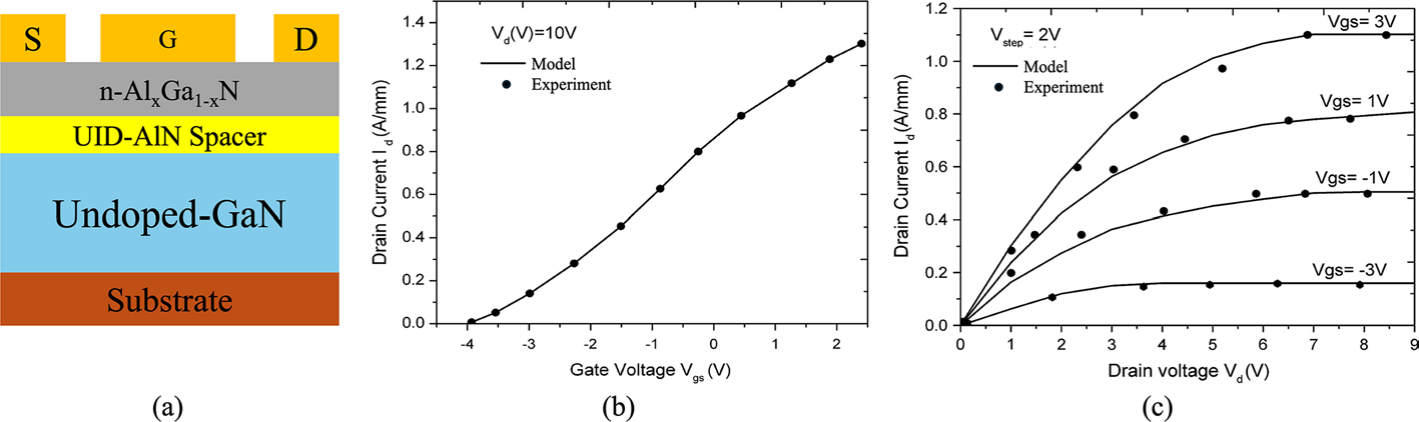
**a** Cross-sectional structure of AlGaN/AlN/GaN HEMT, **b** comparison of transfer characteristics and **c** output characteristics between simulation results and experimental data [126]. Copyright 2014, Mohanbabu et al., published by Elsevier

To investigate the impact of the CCS effect on device performance, Xia et al. employed a carbon nanotube TFET as an experimental test platform [185]. Their findings confirmed that electron-electron scattering introduces additional current pathways, leading to an increase in both the off-state current and the subthreshold swing. Mohanbabu et al. simulated AlGaN/AlN/GaN HEMT devices to analyze surface carrier density, DC characteristics, and microwave frequency performance in HEMT power devices with nano-spacer layers [126]. The HEMT structure is illustrated in Fig. 18(a), while the simulations employed the Conwell-Weisskopf (CW) mobility model. Figure 18(b) compares the simulated and experimental Id-Vd characteristics of the AlGaN/AlN/GaN HEMT with spacer layers, and Fig. 18(c) presents the comparison of Id-Vg characteristics. The strong agreement between simulation and experimental results confirms that the CW model effectively captures carrier mobility under the influence of the CCS effect.

Selecting an appropriate mobility model is crucial for accurately simulating HEMTs, as different models capture distinct transport mechanisms. Table 7 summarizes the key mechanisms, key parameters, advantages, and limitations of various mobility models. High-field models, such as FLDMOB and GANSAT, effectively describe velocity saturation but may oversimplify low-field carrier dynamics. Scattering-based models, including Albrecht, Yamaguchi, Brooks-Herring, and Conwell-Weisskopf, offer a more physically detailed representation by incorporating multiple scattering mechanisms, making them suitable for advanced simulations. The selection of a mobility model depends on the specific device characteristics and operating conditions, requiring a balance between computational efficiency and simulation accuracy.

**Table 7:** The conclusion of mobility models

Model	Key Mechanism	Key parameter	Advantage	Limitation
FLDMOB [19]	Field-dependent model	${V}_{sat}$, $\left|{E}_{\parallel }\right|$	Accounts for velocity	Not accurate for low-field transport effects.
ALBRCT [4]	Scattering-limited mobility	N,${T}_{L}$	Includes energy relaxation effects	Parameters from the experiment.
GANSAT [38]	Saturation velocity model	${V}_{sat}$, $\left|E\right|$	Suitable for high-field conditions	Computationally complex.
YAMAGUCHI [187]	Advanced mobility model with scattering effects	${N}_{i}$, ${E}_{\perp }$, E	Includes more scattering mechanisms	Limited applicability outside its calibrated material systems.
BROOKS	Ionized impurity scattering	N, ${F}_{-1/2}$,${T}_{L}$	Theoretical foundation	Ignores high-field velocity saturation.
CONWELL [32]	Ionized impurity scattering (screening effect included)	n, p,${T}_{L}$	Improved accuracy over Brooks-Herring	May require calibration for specific materials

### Transition models

#### Concentration-dependent SRH model (CONSRH)

**Fig. 19:**
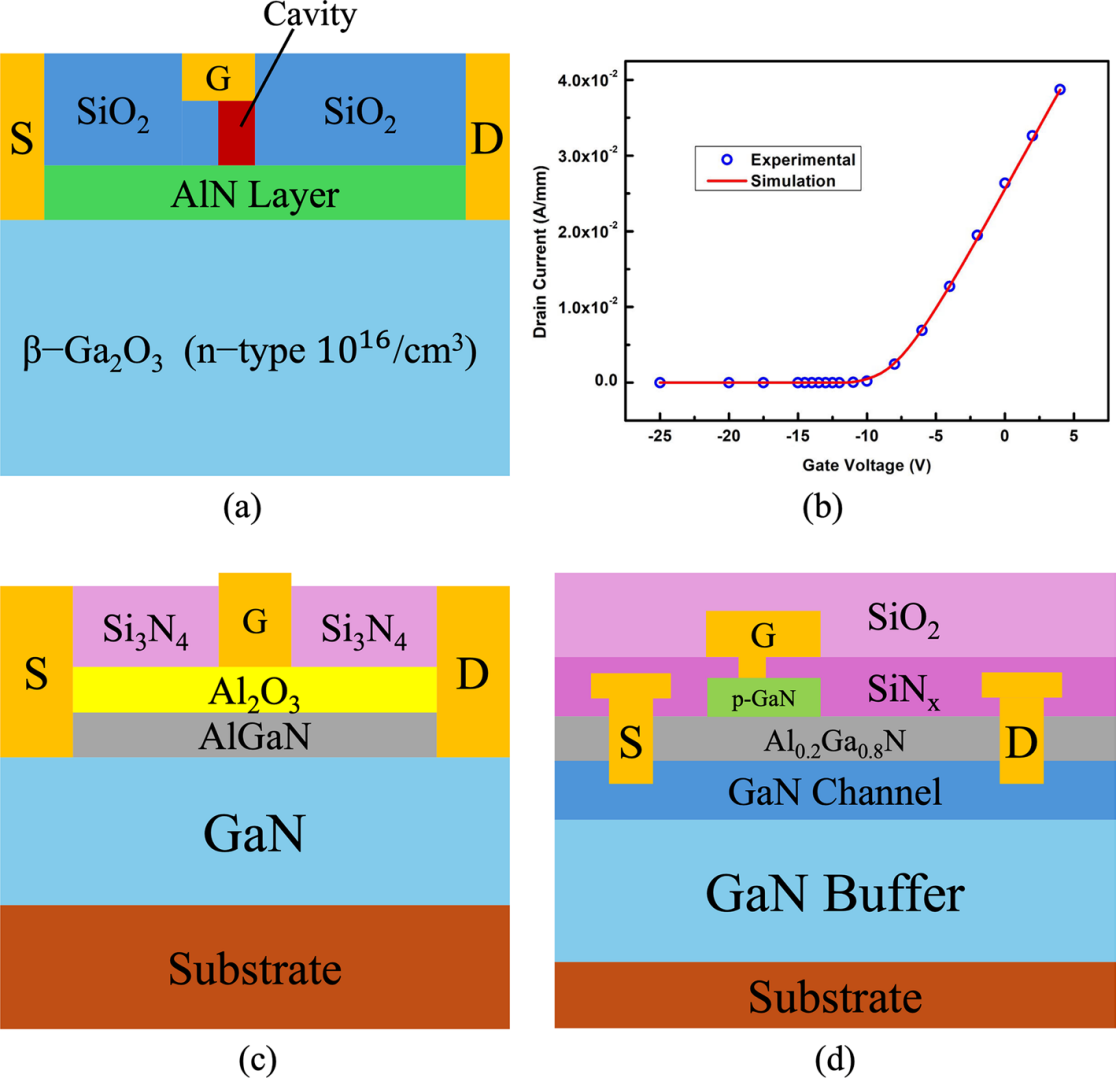
**a** Cross-sectional structure of the AlN/$\beta - {\mathrm{Ga}}_{{2}} {\mathrm{O}}_{{3}}$.HEMT, **b** comparison of simulation and experimental results for AlN/$\beta - {\mathrm{Ga}}_{{2}} {\mathrm{O}}_{{3}}$ HEMT. Reprinted with permission [174]. Under the terms of the Creative Commons Attribution 4.0 license. **c** cross-sectional structure of the AlGaN/GaN HEMT [78], and **d** cross-sectional structure of the ${\mathrm{HV}}_{{\mathrm{T}}}$-HEMT [75]

Tomar et al. analyzed the characteristics of cavity-based AlN/$\beta - {\mathrm{Ga}}_{{2}} {\mathrm{O}}_{{3}}$ MOS-HEMTs through TCAD simulations [174]. The MOS-HEMT device structure is illustrated in Fig. 19(a). Figure 19(b) presents the comparison of transfer characteristics between the simulation results and experimental data, showing strong consistency [62]. Given that the $\beta - {\mathrm{Ga}}_{{2}} {\mathrm{O}}_{{3}}$ buffer layer is n-type doped at a concentration of ${10}^{{{16}}} {\mathrm{/cm}}^{{3}}$, it is reasonable to use the concentration-dependent SRH (CONSRH) model to explain the effect of doping concentration on the recombination process. The CONSRH model extends the standard SRH model described in Eq. (48) by incorporating the carrier lifetime dependence on doping concentration, expressed as follows [44, 97, 153]:

95 $$ \tau_{n - dop} = \left[ {\frac{{N_{\tau 0} }}{{1 + \frac{{N_{T} }}{{5 \times 10^{16} }}}}} \right]\left( {\frac{{T_{L} }}{300K}} \right)^{{L_{T.\tau } }} $$

96 $$ \tau_{p - dop} = \left[ {\frac{{P_{\tau 0} }}{{1 + \frac{{N_{T} }}{{5 \times 10^{16} }}}}} \right]\left( {\frac{{T_{L} }}{300K}} \right)^{{L_{T.\tau } }} $$

where ${\mathrm{N}}_{\mathrm{T}}$ is the total dopant concentration. For carrier statistics, simple Maxwell-Boltzmann statistics are usually used, such as Eqs. (59) and (60). This approach is rooted in Fermi-Dirac statistics.

Kanrar et al. employed the concentration-dependent Shockley-Read-Hall (CONSRH) model in their TCAD simulation of AlGaN/GaN HEMTs with Al_2_O_3_ and Si_3_N_4_ passivation layers, as shown in Fig. 19(c) [78]. This model was specifically utilized to refine the carrier recombination processes within the device. In their study, the authors considered n-type doping concentrations of ${10}^{16}$, ${10}^{17}$, and $10^{{18}} {\mathrm{cm}}^{{ - 3}}$, which were introduced through Si impurity doping. The simulation results were compared with experimental data, demonstrating that the incorporation of the CONSRH model allows for the adjustment of carrier lifetime in the conventional SRH model, thereby providing a more accurate representation of carrier recombination effects in the HEMT structure [63]. Similarly, Jin et al. performed TCAD simulations on a p-GaN/AlGaN/GaN ${HV}_{T}$-HEMT structure with SiO_2_ and SiN_x_ passivation layers, as illustrated in Fig. 19(d) [75]. Their study focused on the impact of p-type doping in the p-GaN layer on device performance. The CONSRH model was integrated into their simulations to adjust the carrier lifetime, ensuring a more accurate representation of carrier trapping and recombination effects. This adjustment was particularly important in modeling the behavior of holes within the p-GaN layer and its influence on overall device performance.

#### Klaassen’s temperature-dependent auger recombination model (KLAAUG)

Compared to the standard Auger recombination model described in Eq. (51), Klaassen’s temperature-dependent Auger recombination model introduces a refinement by redefining the coefficient functions for electrons and holes [88]. In this model, the Auger recombination coefficients are expressed as temperature-dependent parameters, providing a more accurate representation of carrier recombination behavior across a range of temperatures. The modified coefficient functions are given by Eqs. (97) and (98).

97 $$ C_{n} = 1.83 \times 10^{ - 31} \cdot \left( {\frac{{T_{L} }}{300K}} \right)^{1.18} $$

98 $$ C_{p} = 2.78 \times 10^{ - 31} \cdot \left( {\frac{{T_{L} }}{300K}} \right)^{0.72} $$

**Fig. 20:**
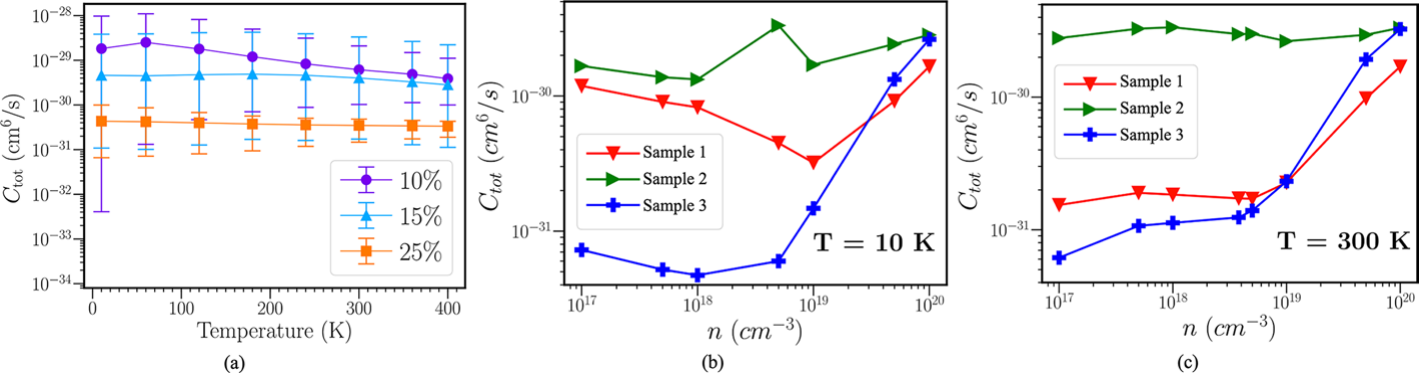
**a** total Auger coefficient of (In,Ga)N/GaN quantum trapped planes as the functions of temperature (10%, 15%, and 25%). Reproduced with permission from ref. [117]. Copyright 2022 American Physical Society. Dependence of the total Auger recombination coefficient on carrier density at **b** T = 10 K and **c** T = 300 K for 3 samples. Reproduce from IOP science under the terms of the Creative Commons Attribution 4.0 license [118]

McMahon et al. studied the temperature dependence of Auger recombination in (In, Ga)N/GaN quantum trap planes, as shown in Fig. 20(a) [117]. The figure illustrates the functional relationship between the total Auger coefficient and temperature for (In, Ga)N/GaN quantum trap planes with 10%, 15%, and 25% In content. When the In content is low, the total Auger coefficient exhibits significant temperature dependence, which strongly affects the recombination efficiency of (In, Ga)N. Therefore, the Klaassen temperature-dependent Auger recombination model is applicable to HEMTs with low-In-content III-nitride barrier layers and GaN channels.

#### Temperature and concentration dependent auger recombination model (HNSAUG)

The coefficients in the standard Auger recombination model are also influenced by carrier density [66]. A higher carrier density enhances carrier interactions, which can lead to a decrease in the Auger recombination rate [53]. McMahon et al. also found that the previously mentioned study on the relationship between the Auger recombination coefficient and temperature was conducted only at moderate carrier densities [118]. To address this limitation, the model was extended to account for variations in the Auger recombination rate in (In, Ga)N/GaN quantum trap planes as a function of carrier density. Figure 20(b) and (c) illustrates the influence of carrier concentration on the total Auger recombination coefficient at 10K and 300K for three samples with different alloy configurations. At 10K, the impact of carrier concentration on the coefficient varies across samples, with all exhibiting a decrease in the Auger coefficient at low doping concentrations, followed by a significant increase beyond a certain threshold. In contrast, at 300K, the total Auger coefficient consistently increases with rising carrier concentration across all samples, highlighting a temperature-dependent variation in the recombination process. Specifically, the coefficient increases from ${10}^{-31}$ to $10^{{ - 30}} {\mathrm{cm}}^{{\mathrm{6}}} {\mathrm{/s}}$ as the carrier concentration rises. Such variations are generally significant enough to impact the efficiency of (In, Ga)N-based devices. The auger recombination model with temperature and concentration dependent coefficients are given by Eqs. (99) and (100) [66].

99 $$ C_{n} = \left[ {6.7 \times 10^{ - 32} + 2.45 \times 10^{ - 31} \left( {\frac{{T_{L} }}{300K}} \right) + \left( { - 2.2 \times 10^{ - 32} } \right)\left( {\frac{{T_{L} }}{300K}} \right)^{2} } \right]\left[ {1 + 3.4667exp\left( { - \frac{n}{{10^{18} }}} \right)} \right] $$

100 $$ C_{p} = \left[ {7.2 \times 10^{ - 32} + 4.5 \times 10^{ - 33} \left( {\frac{{T_{L} }}{300K}} \right) + 2.63 \times 10^{ - 32} \left( {\frac{{T_{L} }}{300K}} \right)^{2} } \right]\left[ {1 + 8.25688exp\left( { - \frac{p}{{10^{18} }}} \right)} \right] $$

Table 8 summarizes the characteristics of various transition models used in semiconductor simulations. The Concentration-Dependent SRH (CONSRH) Model refines recombination modeling by incorporating doping concentration effects, making it more accurate for heavily doped materials such as β-Ga_2_O_3_ and AlGaN/GaN HEMTs. The Klaassen’s Temperature-Dependent Auger Recombination (KLAAUG) Model improves upon standard Auger recombination by introducing temperature dependence, making it particularly useful for III-nitride devices with low-In-content barriers and GaN channels. The Temperature and Concentration-Dependent Auger Recombination (HNSAUG) Model extends KLAAUG by including carrier density dependence, which enhances accuracy in high-concentration regions.

**Table 8:** The summary of transitions model

Model name	Key mechanism	Key parameters	Advantages	Limitations
CONSRH [44, 97, 153]	Doping concentration dependent model	${N}_{T}$	More accurate doping concentration-dependent recombination model	Does not include temperature variations
KLAAUG [88]	Temperature dependent model	${T}_{L}$	Temperature-dependent recombination effects for	Ignores carrier concentration effects
HNSAUG [66]	Temperature and doping concentration dependent model	${E}_{nI}$, ${E}_{pI}$,${T}_{L}$	Based on the KLAAUG model, additional consideration is given to the change in carrier concentration	More complex; requires additional fitting parameters

### Impact ionization models

#### Tabulated impact ionization model based on Selberherr’s model (SELB)

The Selberherr impact ionization model exhibits different behaviors in different crystal structures of the same material. Oguzman et al. theoretically investigated hole-induced impact ionization in the zincblende and wurtzite phases of GaN using Monte Carlo simulations [135]. Their results showed that the hole ionization coefficient in zincblende GaN is significantly higher than in wurtzite GaN, especially at low electric fields, due to the lower average hole energy in the wurtzite phase. Under high electric fields, the hole ionization coefficient in zincblende GaN gradually surpasses that of electrons, remaining around ${10}^{4}$, whereas in wurtzite GaN, the electron ionization coefficient is approximately 10, which is significantly lower than the hole ionization coefficient of around ${10}^{3}$. Therefore, when applying the Selberherr model, parameter selection should consider differences in material structure.

McClintock et al. investigated hole-initiated multiplication in back-illuminated GaN avalanche photodiodes (APDs) to determine ionization coefficients and noise characteristics [116]. The models are given by Eqs. (101) and (102).

101 $$ \alpha_{n} = \frac{V}{E}\left( {\frac{{M_{n} \left( V \right) - 1}}{{M_{n} \left( V \right) - M_{p} \left( V \right)}}} \right)\ln \left( {\frac{{M_{p} \left( V \right)}}{{M_{n} \left( V \right)}}} \right) $$

102 $$ \alpha_{p} = \frac{V}{E}\left( {\frac{{M_{p} \left( V \right) - 1}}{{M_{p} \left( V \right) - M_{n} \left( V \right)}}} \right)\ln \left( {\frac{{M_{n} \left( V \right)}}{{M_{p} \left( V \right)}}} \right) $$

where ${\mathrm{M}}_{\mathrm{n}}$ and ${\mathrm{M}}_{\mathrm{p}}$ are the multiplication factors of electrons and holes. The study demonstrated that hole ionization dominates over electron ionization in GaN, contrary to most III-V semiconductors. By fabricating and characterizing APDs with varying intrinsic layer thicknesses, they extracted ionization coefficients and observed a critical electric field of 2.73 MV/cm. Their results showed that back illumination leads to higher multiplication factors and lower excess noise compared to front illumination, confirming the advantage of hole-initiated processes in GaN APDs. These findings provide valuable insights into impact ionization mechanisms in GaN, which are crucial for the development of low-noise, high-gain ultraviolet photodetectors.

#### Van-Overstraeten-de-man impact ionization model (VANOVERS)

**Fig. 21:**
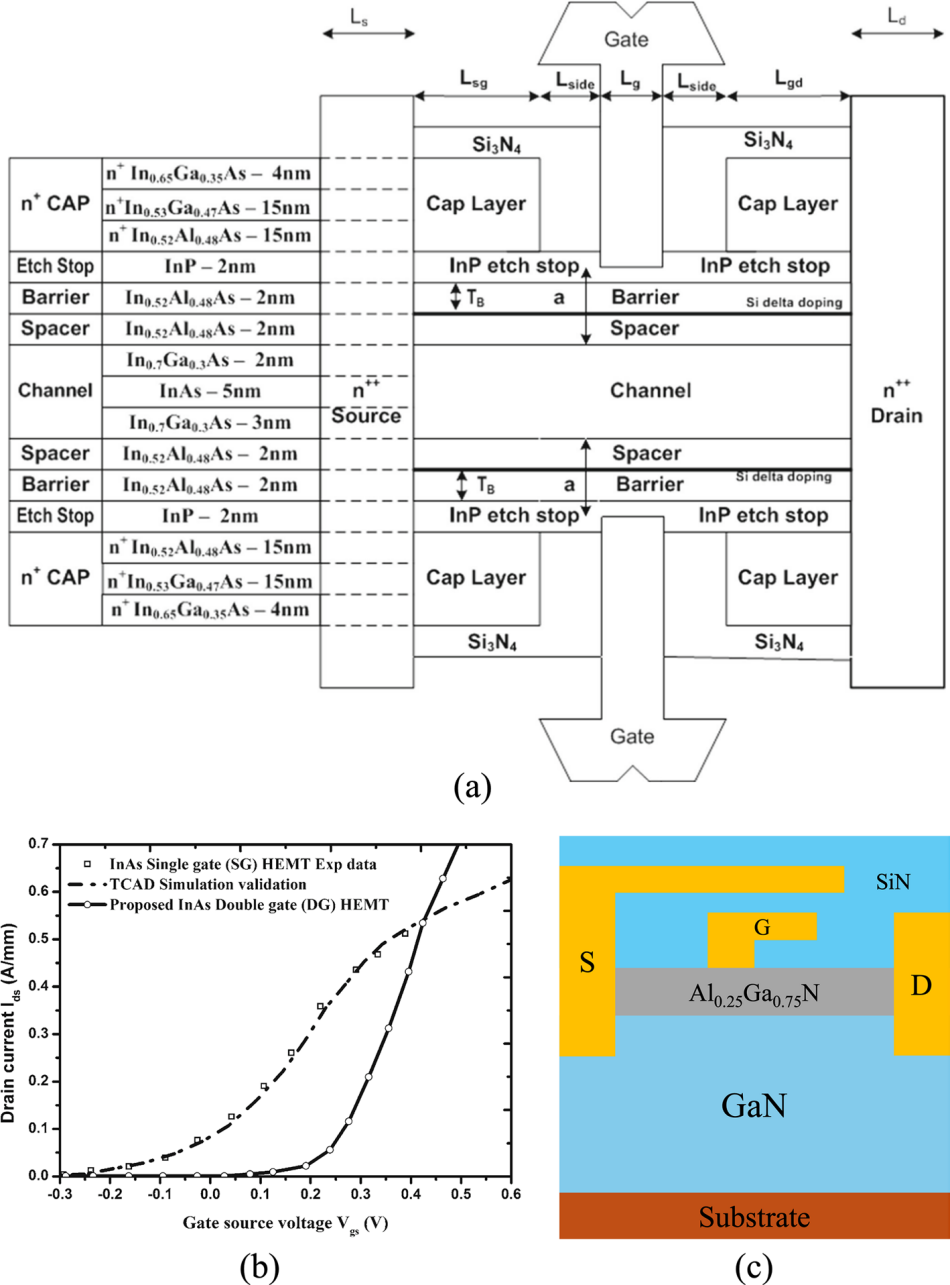
**a** Cross-sectional structure of the InGaAs/InAs/InGaAs composite channel dual-gate HEMT, and **b** transfer characteristics comparing experimental results, simulation verification, and the proposed dual-gate HEMT. Reprinted under terms of the CC-BY license [157]. Copyright 2017, Saravana et al., published by Springer Nature. **c** Cross-sectional structure of a double field plate AlGaN/GaN HEMT [109].

The van Overstraeten and de Man model and the Selberherr model are both variants of the Chynoweth model [30, 176]. However, the temperature dependence parameters in this model differ from those in the Selberherr model. Notably, the exponent on the field dependence is set to 1, and the ionization rates are given by Eqs. (103) and (104).

103 $$ \alpha_{n} = \gamma_{n} A_{n} exp\left[ { - \left( {\frac{{\gamma_{n} B_{n} }}{{\left| {E_{nI} } \right|}}} \right)} \right] $$

104 $$ \alpha_{p} = \gamma_{p} A_{p} exp\left[ { - \left( {\frac{{\gamma_{p} B_{p} }}{{\left| {E_{pI} } \right|}}} \right)} \right] $$

105 $$ \gamma_{n} = \frac{{tanh\left( {\frac{0.063}{{2k \cdot 300k}}} \right)}}{{tanh\left( {\frac{0.063}{{2k \cdot T_{L} }}} \right)}} $$

106 $$ \gamma_{p} = \frac{{tanh\left( {\frac{0.063}{{2k \cdot 300k}}} \right)}}{{tanh\left( {\frac{0.063}{{2k \cdot T_{L} }}} \right)}} $$

where the value of 0.063 eV corresponds to the default photophonon energy of Si [115]. The coefficients ${\mathrm{A}}_{\mathrm{n}}$, ${\mathrm{A}}_{\mathrm{p}}$, ${\mathrm{B}}_{\mathrm{n}}$ and ${\mathrm{B}}_{\mathrm{p}}$ are the same as those in the Selberherr model, using the same parameters. Kumar et al. conducted TCAD simulations of an InGaAs/InAs/InGaAs composite channel dual-gate HEMT power device, utilizing the van Overstraeten-de Man model as the impact ionization model to analyze the dependence of the impact ionization rate on the carrier energy distribution [157]. Figure 21(a) illustrates the structure of the dual-gate HEMT, while Fig. 21(b) presents the simulated and experimental transfer characteristics. The TCAD simulation results closely align with the experimental data, demonstrating the model’s accuracy [21]. Additionally, the dual-gate HEMT exhibits a higher threshold voltage and saturation current compared to conventional designs, further validating the effectiveness of the simulation approach.

Similarly, Liu et al. employed an artificial neural network model to establish the relationship between the structural parameters and breakdown voltage of a double field plate AlGaN/GaN HEMT, enabling rapid prediction of breakdown performance [107, 109]. The breakdown voltage was obtained through TCAD simulations using the van Overstraeten-de Man impact ionization model. Figure 21(c) illustrates the structure of the double field plate AlGaN/GaN HEMT. The simulated breakdown voltage results closely match the experimental data, showing a breakdown voltage of approximately 100 V in the absence of a field plate and increasing to approximately 460 V when the field plate length reaches 5. Furthermore, the observed trend—where the breakdown voltage saturates beyond a certain ${L}_{SFP}$ length—is consistent with simulation results from other field plate HEMTs, further validating the accuracy of the model [104].

#### Valdinoci’s impact ionization model (VALDINOCI)

Valdinoci et al. conducted theoretical and experimental studies on electron impact ionization in silicon over a temperature range of 25 to 400 °C and proposed a new compact model for the impact ionization coefficient, calibrated specifically for silicon within this temperature range [175]. The model is given by Eqs. (107) and (108).

107 $$ \alpha_{n} = \frac{{\left| {E_{nI} } \right|}}{{\left[ {4.3383 + \left( { - 2.42 \times 10^{ - 12} } \right)\left( {\frac{{T_{L} }}{1K}} \right)^{4.1233} } \right] + exp\left( {\frac{{1.233735 \times 10^{6} + 1.2039 \times 10^{3} \left( {\frac{{T_{L} }}{1K}} \right) + 0.56703\left( {\frac{{T_{L} }}{1K}} \right)^{2} }}{{\left| {E_{nI} } \right| + \left[ {1.6831 \times 10^{4} + 4.3796\left( {\frac{{T_{L} }}{1K}} \right) + 0.13005\left( {\frac{{T_{L} }}{1K}} \right)^{2} } \right]}}} \right)}} $$

108 $$ \alpha_{p} = \frac{{\left| {E_{nI} } \right|}}{{\left[ {2.376 + \left( {0.01033} \right)\left( {\frac{{T_{L} }}{1K}} \right)} \right] + 0.17714exp\left[ { - 0.002178\left( {\frac{{T_{L} }}{1K}} \right)} \right]exp\left( {\frac{{1.4043 \times 10^{6} + 2.9744 \times 10^{3} \left( {\frac{{T_{L} }}{1K}} \right) + 1.4829\left( {\frac{{T_{L} }}{1K}} \right)^{2} }}{{\left| {E_{nI} } \right| + \left[ {0.00947\left( {\frac{{T_{L} }}{1K}} \right)^{2.4924} } \right]}}} \right)}} $$

**Fig. 22:**
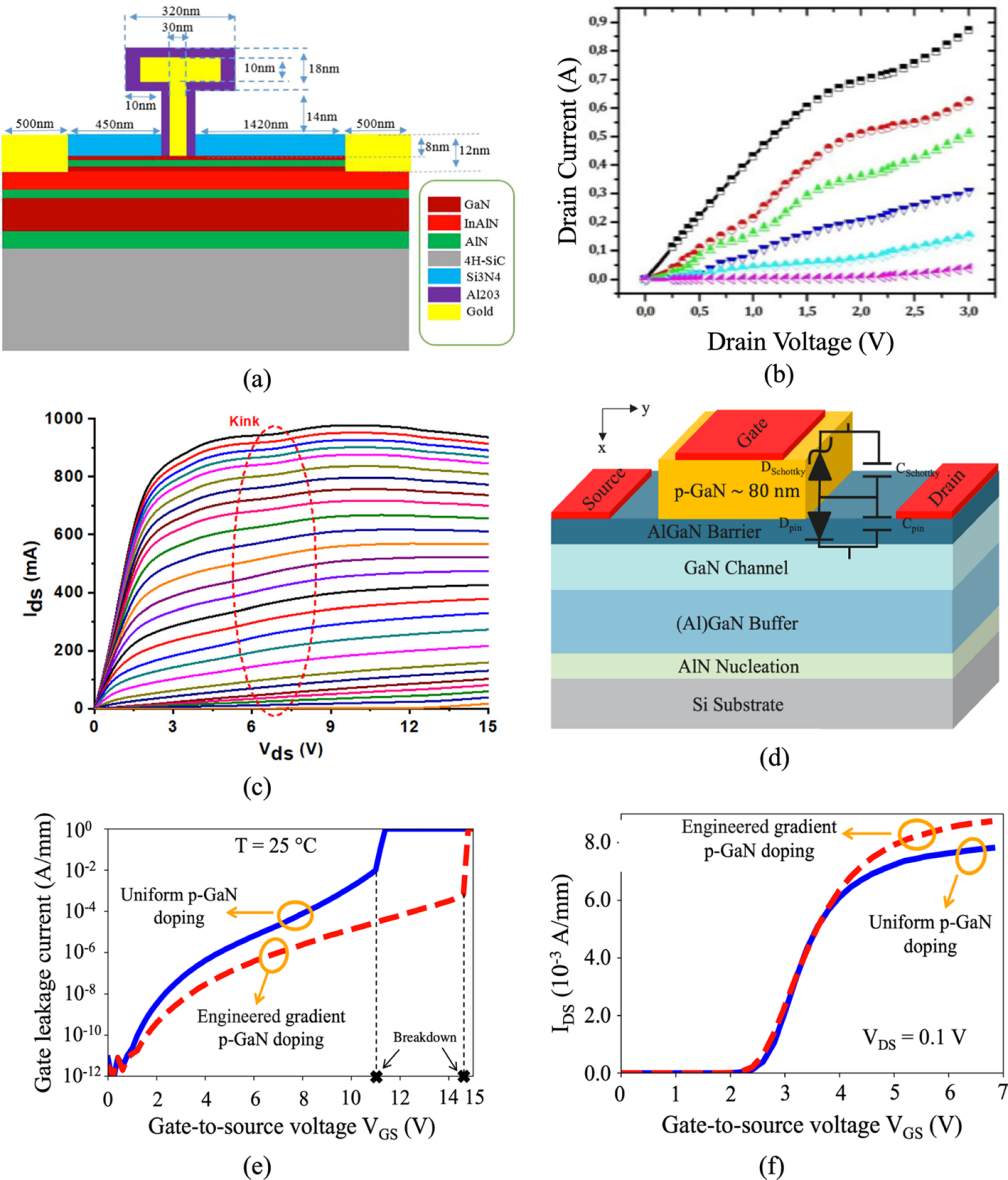
**a** Cross-sectional structure of the InAlN/GaN HEMT. **b** Simulated output characteristics showing the kink effect. Reprinted with permission from ref. [91]. Copyright 2015 Elsevier. **c** Experimentally observed kink effect in AlGaN/GaN/SiC HEMT. Reprinted with permission from ref. [5]. Copyright 2020 Elsevier. **d** Schottky gate HEMT structure with uniform p-GaN doping. **e** Gate leakage current comparison between uniform p-GaN doped HEMT and engineered gradient p-GaN doped HEMT. **f** Drain current characteristics of uniform and engineered gradient p-GaN doped HEMTs. Reprinted with permission [2]. Under the terms of the Creative Commons Attribution 4.0 license

Kourdi et al. used Silvaco TCAD to study the side effects of InAlN/GaN HEMT power devices, with the structure shown in Fig. 22(a) [91]. When using the Selberherr impact ionization model to analyze the Kink effect, Silvaco TCAD struggled to accurately simulate the phenomenon [50]. The Valdinoci model effectively resolved this issue, as it supports simulations up to 400 °C. Figure 22(c) illustrates the impact of the Kink effect on output characteristics, where a slight current reduction occurs in the saturation region due to increased temperature [5]. Figure 22(b) presents the simulated output characteristics of the InAlN/GaN HEMT, successfully capturing the Kink effect, confirming that the Valdinoci model accurately simulates temperature-dependent behaviors.

#### Okuto-crowell impact ionization model (OKUTO)

The Okuto-Crowell model is a temperature-dependent impact ionization model that incorporates the effect of threshold energy on the ionization process in semiconductors [136]. Okuto et al. predicted the influence of band bending and temperature on avalanche breakdown in semiconductor junctions and experimentally validated these predictions. The ionization coefficients for electron and hole are given by Eqs. (109) and (110).

109 $$ \alpha_{n} = 0.246\left( {1 + 3.05 \times 10^{ - 4} \left( {T_{L} - 300} \right)} \right)\left| {E_{nI} } \right|exp\left( { - \left( {\frac{{4.81 \times 10^{5} \left( {1 + 6.86 \times 10^{ - 4} \left( {T_{L} - 300} \right)} \right)}}{{\left| {E_{nI} } \right|}}} \right)^{2} } \right) $$

110 $$ \alpha_{p} = 0.243\left( {1 + 5.35 \times 10^{ - 4} \left( {T_{L} - 300} \right)} \right)\left| {E_{pI} } \right|exp\left( { - \left( {\frac{{6.53 \times 10^{5} \left( {1 + 5.67 \times 10^{ - 4} \left( {T_{L} - 300} \right)} \right)}}{{\left| {E_{pI} } \right|}}} \right)^{2} } \right) $$

Alaei et al. developed an analytical model for p-GaN gate HEMTs based on rigorous solutions of the Poisson and Schrödinger equations, as shown in Fig. 22(d) [2]. To analyze breakdown characteristics, they applied the Okuto-Crowell model for the ionization coefficient. Figure 22(e) and (f) present the gate leakage current and drain current as functions of forward gate-source voltage ${V}_{gs}$ for both uniform p-GaN HEMT and gradient-engineered HEMT. The results indicate a notable decrease in gate leakage current under forward bias, along with an enhancement in breakdown voltage, while the threshold voltage remains consistent. The alignment between simulation results and experimental measurements validates the accuracy and reliability of the proposed model.

Table 9 summarizes the key characteristics of the four impact ionization models, highlighting their strengths and limitations. The Selberherr model, originally designed for silicon, is widely used due to its simplicity but may require parameter adjustments for wide-bandgap materials like GaN and SiC. The Van Overstraeten-de Man model improves on Selberherr’s approach by incorporating temperature dependence and refined field dependence, making it a preferred choice for silicon-based avalanche breakdown simulations. The Valdinoci model extends the applicability to high-temperature conditions (25–400 °C), offering better accuracy for thermal simulations. The Okuto-Crowell model further refines impact ionization modeling by including threshold energy and band bending effects, making it particularly suitable for breakdown voltage predictions in various semiconductor materials.

**Table 9:** The conclusion of impact ionization models

Model name	Key mechanism	Key parameters	Advantages	Limitations
SELB	Empirical model based on electric field dependence	${E}_{nI}$,${E}_{pI}$	Simple and widely used in TCAD simulations	Require parameter adjustments for wide-bandgap materials like GaN and SiC
VANOVERS [176]	Empirical model with temperature dependence and high-field accuracy	${E}_{nI}$, ${E}_{pI}$,${T}_{L}$	Well-calibrated for Si-based devices, widely used for avalanche breakdown simulations	Not specifically optimized for III-V materials
VALDINOCI [175]	Includes explicit temperature dependence for a wide range (25-400 °C)	${E}_{nI}$, ${E}_{pI}$,${T}_{L}$	Suitable for high-temperature simulations improves accuracy in TCAD	More complex, requires additional parameters
OKUTO [136]	Incorporates threshold energy and band bending effects	${E}_{nI}$, ${E}_{pI}$,${T}_{L}$	Band bending effects	Requires detailed material-specific fitting

Experimental validation is essential for ensuring the reliability of GaN HEMT simulations. In most studies, TCAD models are first benchmarked against fabricated device measurements before being applied to more complex structures. This highlights the importance of model calibration, where tuning parameters such as mobility constants, impact ionization coefficients, and recombination rates allows simulations to closely match experimental results. Importantly, it is often not necessary to alter the fundamental form of the physical models; rather, careful parameter refinement within these established frameworks enables accurate reproduction of real device behavior. The consistency reported across the literature confirms that, when properly parameterized, these models provide a robust foundation for GaN HEMT simulation.

**Fig. 23:**
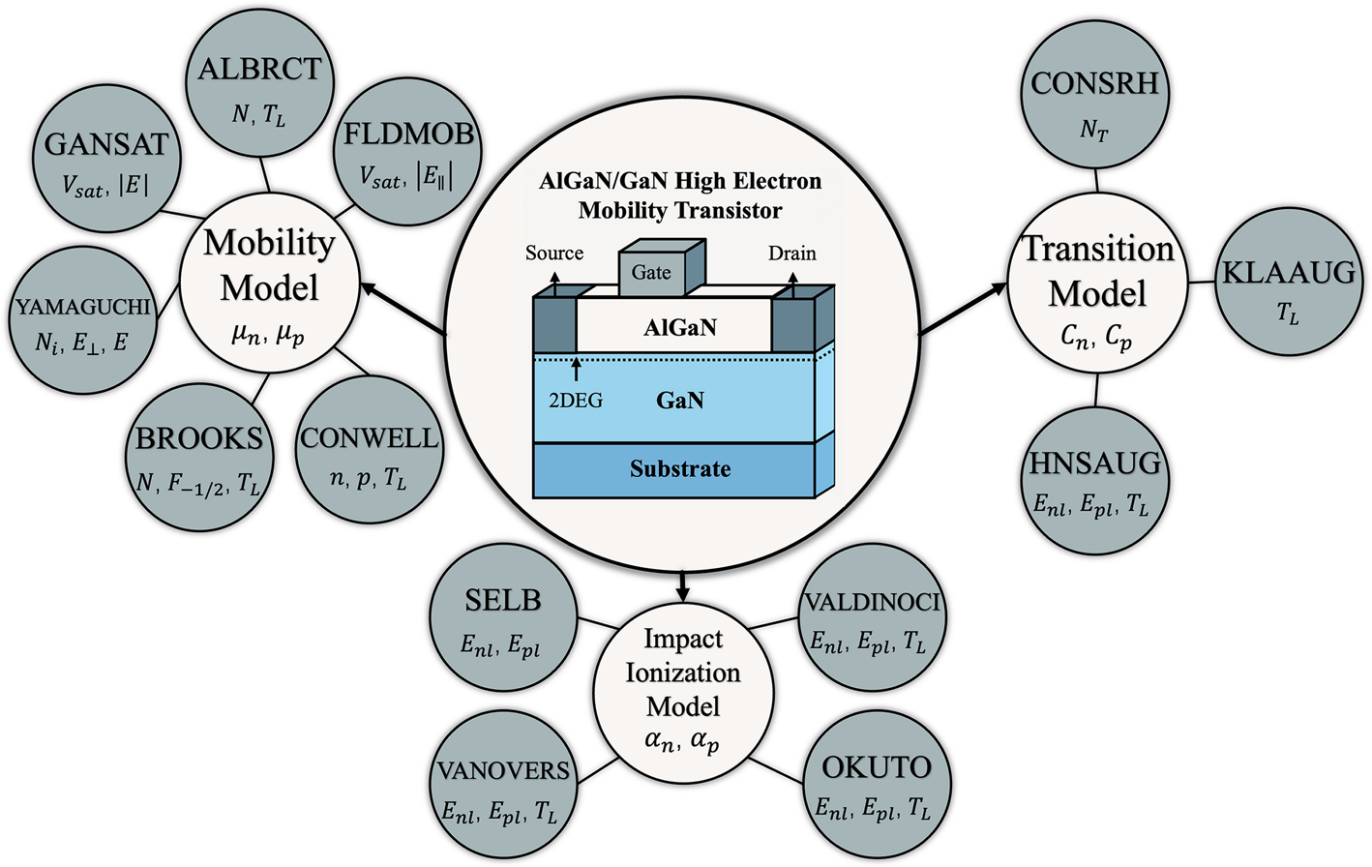
Classification of physical models for GaN HEMT TCAD simulations

To further clarify the scope and applicability of these models, Table 10 summarizes the physical models reviewed in this section, grouped into three categories: mobility models, transition models, and impact ionization models. The table also indicates the semiconductor materials for which each model is most suitable, serving as a practical reference for simulation studies. In addition, Fig. 23 presents a visual classification of these models, showing their relation to the GaN HEMT device structure. Ultimately, this comparative overview highlights that aligning model selection and parameter calibration with the specific operating regime is essential for accurate and efficient HEMT simulations. While appropriate choices ensure consistency with experimental validation and optimize computation time, activating unnecessary physical mechanisms forces the solver to calculate irrelevant parameters, needlessly prolonging the simulation without yielding any additional physical insight.

**Table 10:** Summary of suitable materials corresponding to physical models

Category	Models name	Suitable materials
Mobility models	FLDMOB	General semiconductors
	ALBRCT	GaN, AlGaN (III-V materials)
	GANSAT	GaN, AlGaN (III-V materials)
	YAMAGUCHI	GaN, AlGaN (III-V materials)
	BROOKS	Doped semiconductors, GaN/AlGaN
	CONWELL	Doped semiconductors, GaN/AlGaN
Transition models	CONSRH	General semiconductors including GaN
	KLAAUG	GaN, AlGaN
	HNSAUG	GaN, AlGaN
Impact ionization models	SELB	General semiconductor (including GaN/AlGaN)
	VANOVERS	General semiconductor (Si, GaAs, GaN, AlGaN)
	VALDINOCI	General semiconductor (Si, GaAs, GaN, AlGaN)
	OKUTO	GaN, AlGaN

## Conclusion

This paper provides a comprehensive analysis of the physical models used in TCAD simulations for HEMT power devices, focusing on mobility, transition, and impact ionization models. By evaluating their applicability and accuracy in different HEMT materials and structures, this study aims to identify the most appropriate physical models to improve simulation accuracy.

Section 2 introduces the structure of HEMTs and the mechanism of 2DEG formation driven by spontaneous polarization. Additionally, it systematically discusses critical device parameters and their calculation methods, including bandgap energy, metal work function, electron affinity, dielectric constant, 2DEG concentration, and carrier lifetime. These parameters are essential for accurately modeling device performance and optimizing HEMT design.

Section 3 focuses on the role of TCAD in semiconductor device simulation, particularly in modeling AlGaN/GaN HEMTs. The Silvaco Victory Device simulator serves as a robust platform for solving coupled nonlinear partial differential equations (PDEs), including Poisson’s equation, carrier continuity equations, and heat flow and energy transport equations. These equations, derived from fundamental physical principles such as Maxwell’s laws, govern the interaction between electrostatic potential, carrier density, and transport mechanisms. By discretizing these equations into nonlinear algebraic systems and solving them iteratively using numerical techniques, TCAD enables precise device simulation. This facilitates in-depth analysis, optimization, and performance prediction of advanced semiconductor devices, underscoring the critical role of TCAD in driving technological advancements across various semiconductor applications.

To address the need for enhanced accuracy, Sect. 4 explores advanced physical models implemented in TCAD across different material systems and device architectures. These include specialized mobility models that account for velocity saturation, impurity scattering, and field-dependent effects, as well as composite models incorporating temperature and carrier concentration dependencies. Additionally, various impact ionization models are examined to assess their effectiveness in predicting breakdown voltage and avalanche effects. By integrating these advanced models, this study provides essential insights for optimizing TCAD simulations to ensure more realistic and reliable predictions of HEMT behavior.

Carrier transport models are fundamental to semiconductor device simulations, as they dictate how charge carriers move under external fields and interact with scattering mechanisms. Several advanced mobility models have been analyzed, including FLDMOB, Albrecht, Gansat, Yamaguchi, Brooks-Herring, and Conwell-Weisskopf models. Each model captures distinct physical effects such as field-dependent velocity variations, impurity scattering, and temperature dependence, leading to a more accurate representation of carrier dynamics. The FLDMOB model effectively accounts for velocity overshoot in high-field regions, while the Albrecht and Yamaguchi models incorporate nonlocal transport effects, making them essential for simulating short-channel devices. Additionally, the Brooks-Herring and Conwell-Weisskopf models focus on impurity scattering, making them particularly suitable for heavily doped semiconductor regions.

Transition models also play a critical role in determining carrier lifetime and trapping effects, which significantly impact overall device performance. This study highlights the concentration-dependent SRH (CONSRH) model, which refines the standard SRH model by incorporating the influence of doping concentration on carrier recombination rates. Simulations of AlGaN/GaN and β-Ga_2_O_3_ MOSHEMTs demonstrate that the CONSRH model provides a more accurate description of carrier dynamics in heavily doped buffer and passivation layers. Furthermore, temperature-dependent recombination models, such as Klaassen’s Auger recombination model (KLAAUG) and the temperature- and concentration-dependent Auger recombination model (HNSAUG), are examined. These models introduce temperature scaling factors into the Auger recombination coefficient, enhancing simulation accuracy for III-nitride devices with varying compositions. The HNSAUG model further refines this approach by incorporating carrier density dependence, ensuring a dynamic adaptation of the recombination rate to variations in temperature and carrier concentration.

Impact ionization models are essential for predicting breakdown voltage and avalanche multiplication effects in power devices. This review compares the Van-Overstraeten-De-Man, Selberherr, Crowell-Sze, and Okuto-Crowell models, each offering different levels of accuracy based on empirical fitting parameters and physical formulations. The Van-Overstraeten-De-Man model is widely recommended for breakdown voltage simulations due to its precise calibration for silicon devices, whereas the Selberherr model extends its applicability to a broader range of materials but is generally less accurate for non-silicon semiconductors. The Crowell-Sze and Okuto-Crowell models incorporate quantum mechanical tunneling effects, making them more suitable for devices operating under extremely high electric fields. These distinctions highlight the necessity of selecting the appropriate impact ionization model based on the semiconductor material and electric field conditions.

An analysis of these physical models reveals that no single model offers universal advantages in TCAD simulations. Instead, selecting an appropriate model requires balancing computational efficiency with physical accuracy while tailoring it to the specific characteristics of the device under study. While empirical models offer convenient parameterization, physics-based models provide deeper insights into carrier transport, recombination mechanisms, and impact ionization. These models are particularly valuable for enhancing predictive accuracy in device simulations, making them essential for optimizing semiconductor design and performance.

With a comprehensive understanding of advanced TCAD models and the physical mechanisms governing HEMT operation, researchers are now well-equipped to explore integration strategies between HEMTs and other semiconductor devices. Accurate modeling of transport, interface effects, and polarization allows for the prediction of device interactions in hybrid systems, such as HEMT-MOSFET cascodes, HEMT-based power ICs, and multi-functional modules. This capability not only facilitates co-design and optimization across different device types but also accelerates the development of complex circuits that leverage the high electron mobility, high breakdown voltage, and fast switching characteristics of HEMTs. By leveraging detailed simulations, designers can anticipate potential challenges in device coupling, thermal management, and parasitic effects, enabling more seamless integration and higher overall system performance.

Building on this foundation, advancing TCAD models remains equally critical for further improving predictive accuracy and optimizing device performance. Future research should focus on developing physics-based models that incorporate quantum mechanical effects, multiscale transport phenomena, and machine learning–assisted parameter extraction and calibration. Equally important is the integration of experimental validation with simulation, which not only verifies model accuracy but also ensures that simulated results align more closely with fabricated device performance. This alignment reduces discrepancies between theoretical predictions and experimental outcomes, enabling TCAD to serve as a more reliable tool for device design and process optimization. By continuously refining these models, TCAD simulations will play an increasingly vital role in predicting device behavior, accelerating development cycles, and driving breakthroughs in high-power, high-frequency, and energy-efficient semiconductor applications.

## Supplementary Information

Additional file1 (PDF 3865 kb)

## Data Availability

No datasets were generated or analysed during the current study.
