# Supplementary material for: Advanced physical modeling approaches for high-precision TCAD simulation of GaN HEMT power devices: a review
Source: Discov Nano. 2026 Apr 30;21(1):152. doi: 10.1186/s11671-026-04571-0 (PMC13133327; doi:10.1186/s11671-026-04571-0)
Supplement: Supplementary file 1 — Additional file1 (PDF 3865 kb) [file 11671_2026_4571_MOESM1_ESM.pdf]

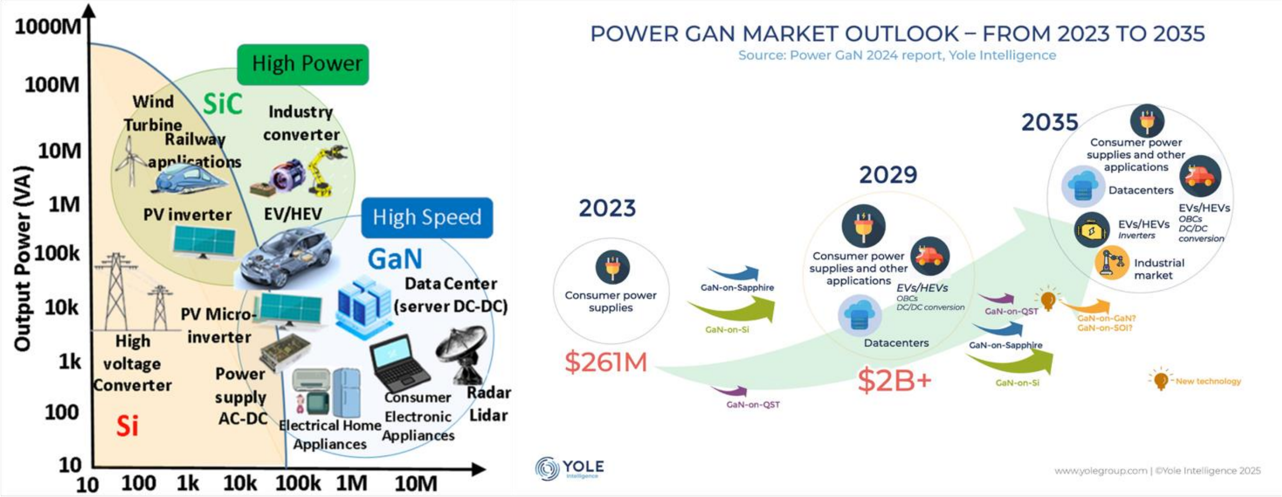

(a) (b)

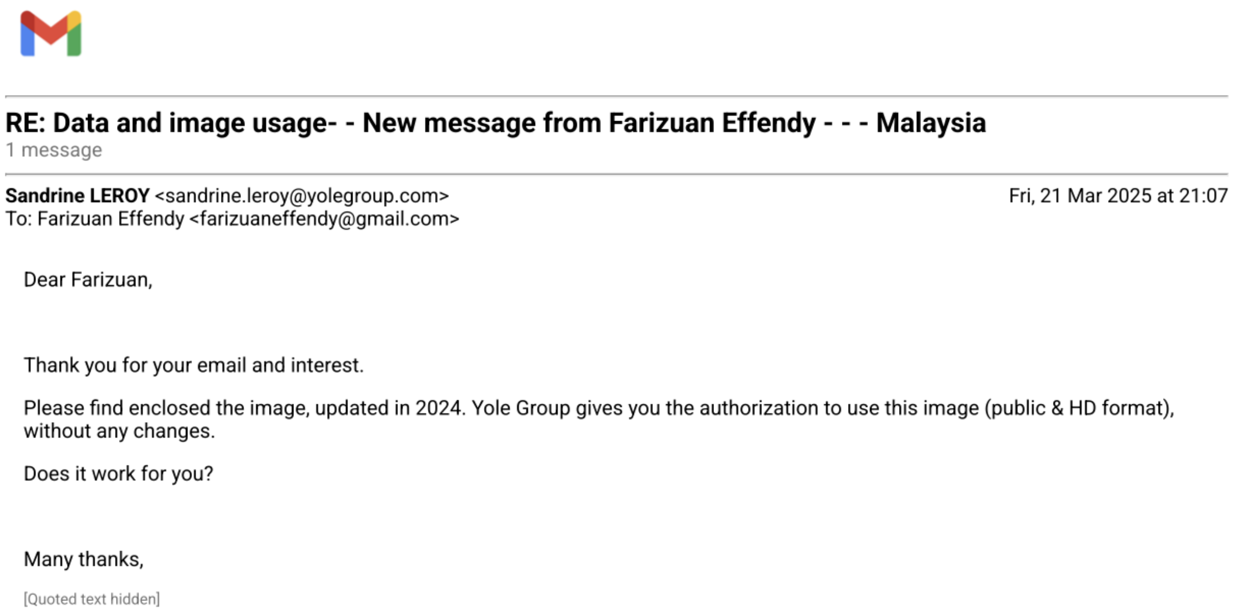
RE: Data and image usage- - New message from Farizuan Effendy - - - Malaysia

1 message

Sandrine LEROY <sandrine.leroy@yolegroup.com> Fri, 21 Mar 2025 at 21:07

To: Farizuan Effendy <farizuaneffendy@gmail.com>

Dear Farizuan,

Thank you for your email and interest.

Please find enclosed the image, updated in 2024. Yole Group gives you the authorization to use this image (public & HD format), without any changes.

Does it work for you?

Many thanks,

[Quoted text hidden]

Figure 1 (a) Application scope based on WBG materials. Reprinted with permission from ref. (Kusters, 2025). Copyright 2023 MDPI. (b) GaN power electronic devices in long-term evolution for the Chinese market (LEROY, 2023). Source Power GaN report, Yole intelligence 2023.

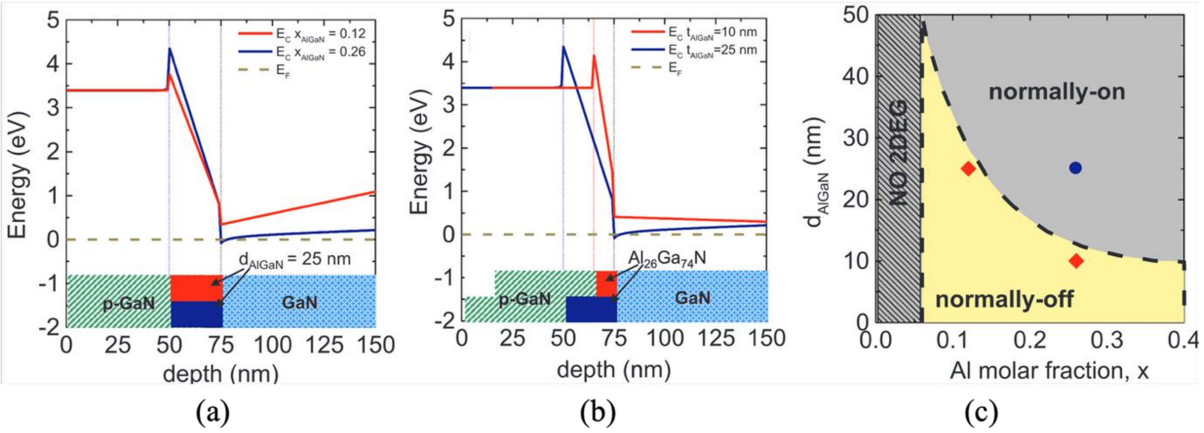

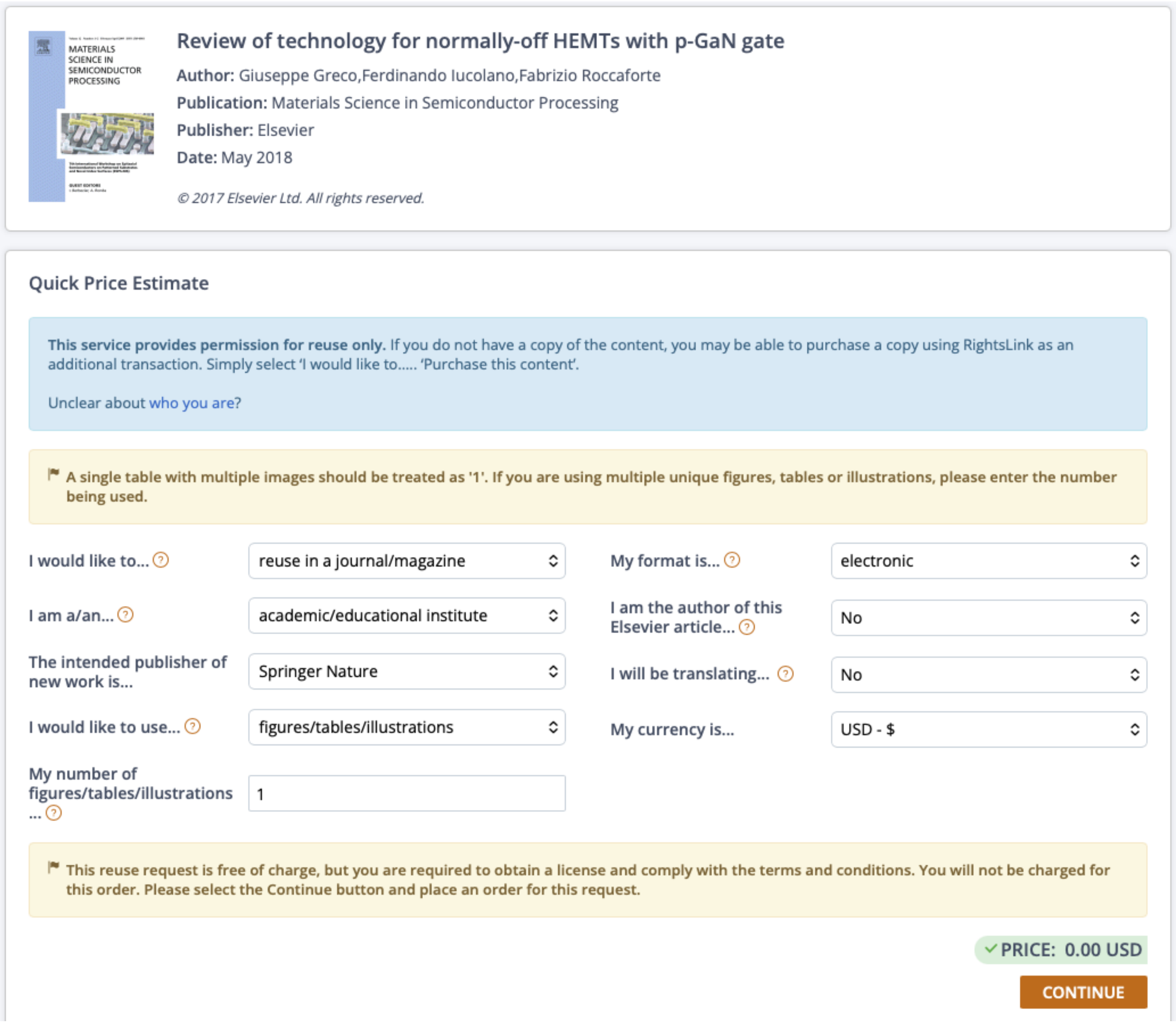

Review of technology for normally-off HEMTs with p-GaN gate

Author: Giuseppe Greco,Ferdinando Iucolano,Fabrizio Roccaforte
Publication: Materials Science in Semiconductor Processing
Publisher: Elsevier
Date: May 2018

© 2017 Elsevier Ltd. All rights reserved.

Quick Price Estimate

This service provides permission for reuse only. If you do not have a copy of the content, you may be able to purchase a copy using RightsLink as an additional transaction. Simply select 'I would like to..... 'Purchase this content'.

Unclear about who you are?

A single table with multiple images should be treated as '1'. If you are using multiple unique figures, tables or illustrations, please enter the number being used.

I would like to...	reuse in a journal/magazine	My format is...	electronic
I am a/an...	academic/educational institute	I am the author of this Elsevier article...	No
The intended publisher of new work is...	Springer Nature	I will be translating...	No
I would like to use...	figures/tables/illustrations	My currency is...	USD - $
My number of figures/tables/illustrations ...	1		

This reuse request is free of charge, but you are required to obtain a license and comply with the terms and conditions. You will not be charged for this order. Please select the Continue button and place an order for this request.

PRICE: 0.00 USD

CONTINUE

Figure 8 Conduction band energy diagrams in p-GaN/AlGaN/GaN HEMTs: (a) varying Al Composition, (b) varying barrier layer thickness (Greco et al., 2018), and (c) Al composition vs. AlGaN thickness for normally-On/Off boundary (Fujii et al., 2007). Copyright 2017 Elsevier.

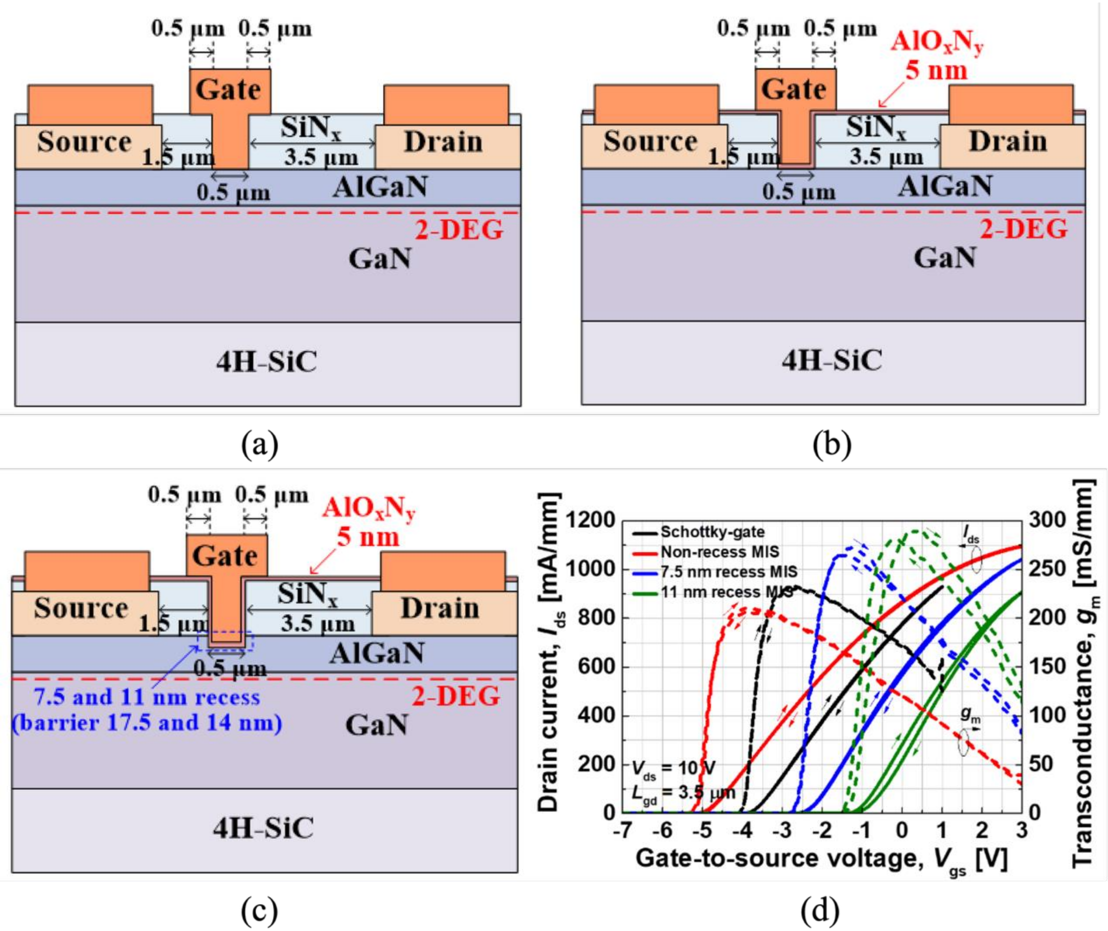

MDPI Open Access Information and Policy

All articles published by MDPI are made immediately available worldwide under an open access license. This means the following:

- Everyone has free and unlimited access to the full texts of *all* articles published in MDPI journals;
- Everyone is free to reuse the published material if the original article is properly accredited and cited;
- Open access publication is supported by the authors' institutes or research funding agencies via the payment of a comparatively low **Article Processing Charge (APC)** for accepted articles.

Permissions

No special permission is required to reuse all or part of articles published by MDPI, including figures and tables. For articles published under an open access Creative Common CC BY license, any part of the article may be reused without permission provided that it is clearly cited. The reuse of an article does not imply endorsement from the authors or MDPI. Furthermore, no special permission is required for authors to submit their research to external repositories. This policy extends to all versions of a paper, including its submitted, accepted, and published forms.

Figure 10 Schematic cross-section of AlGaN/GaN HEMTs: (a) Schottky gate, (b) non-recessed MIS-HEMT, and (c) recessed MIS-HEMT. (d) transfer and transconductance characteristics (H.-S. Kim et al., 2020). Copyright 2023 MDPI.

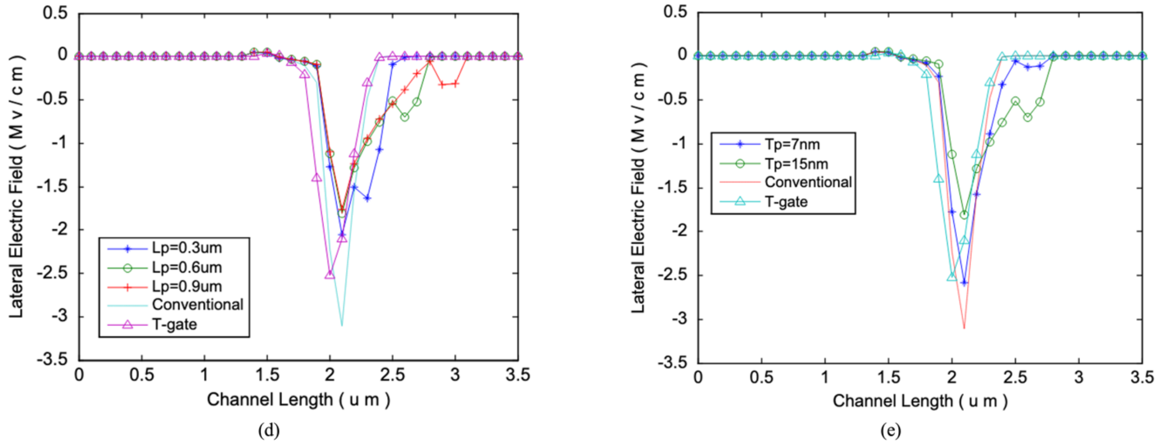

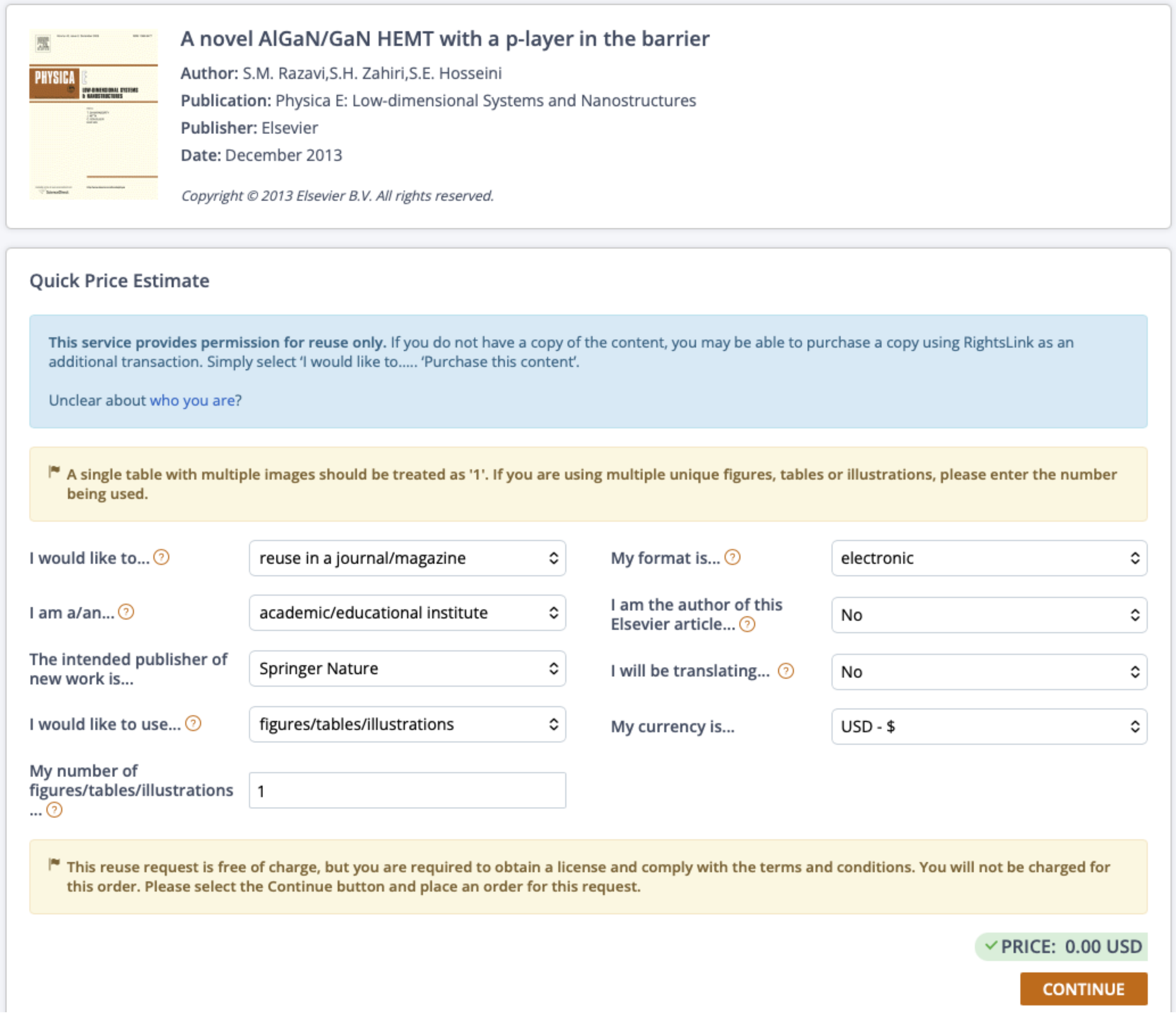

A novel AlGaN/GaN HEMT with a p-layer in the barrier

Author: S.M. Razavi,S.H. Zahiri,S.E. Hosseini

Publication: Physica E: Low-dimensional Systems and Nanostructures

Publisher: Elsevier

Date: December 2013

Copyright © 2013 Elsevier B.V. All rights reserved.

Quick Price Estimate

This service provides permission for reuse only. If you do not have a copy of the content, you may be able to purchase a copy using RightsLink as an additional transaction. Simply select 'I would like to..... 'Purchase this content'.

Unclear about who you are?

A single table with multiple images should be treated as '1'. If you are using multiple unique figures, tables or illustrations, please enter the number being used.

I would like to...	reuse in a journal/magazine
I am a/an...	academic/educational institute
The intended publisher of new work is...	Springer Nature
I would like to use...	figures/tables/illustrations
My number of figures/tables/illustrations ...	1
My format is...	electronic
I am the author of this Elsevier article...	No
I will be translating...	No
My currency is...	USD - $

This reuse request is free of charge, but you are required to obtain a license and comply with the terms and conditions. You will not be charged for this order. Please select the Continue button and place an order for this request.

PRICE: 0.00 USD

CONTINUE

Figure 12 And lateral electric field distribution diagrams of (d) different L_P at fixed T_P= 15 nm and (e) different T_P at fixed L_P=0.6 μm. Reprinted with permission from ref. (Razavi et al., 2013). Copyright 2013 Elsevier.

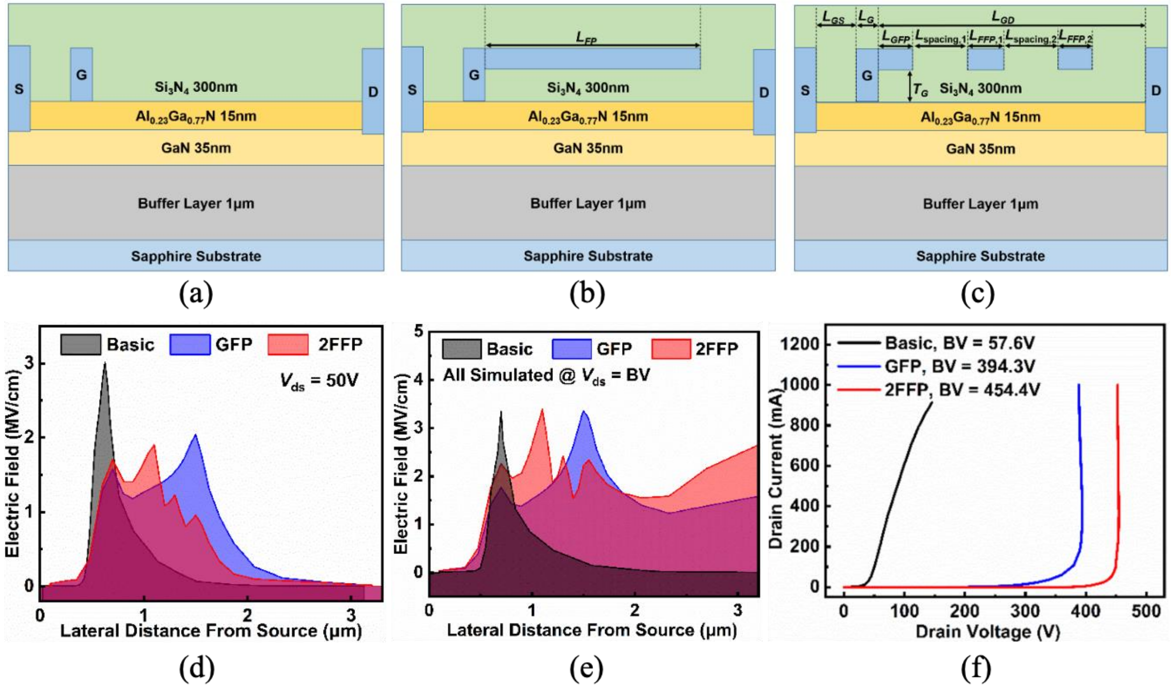

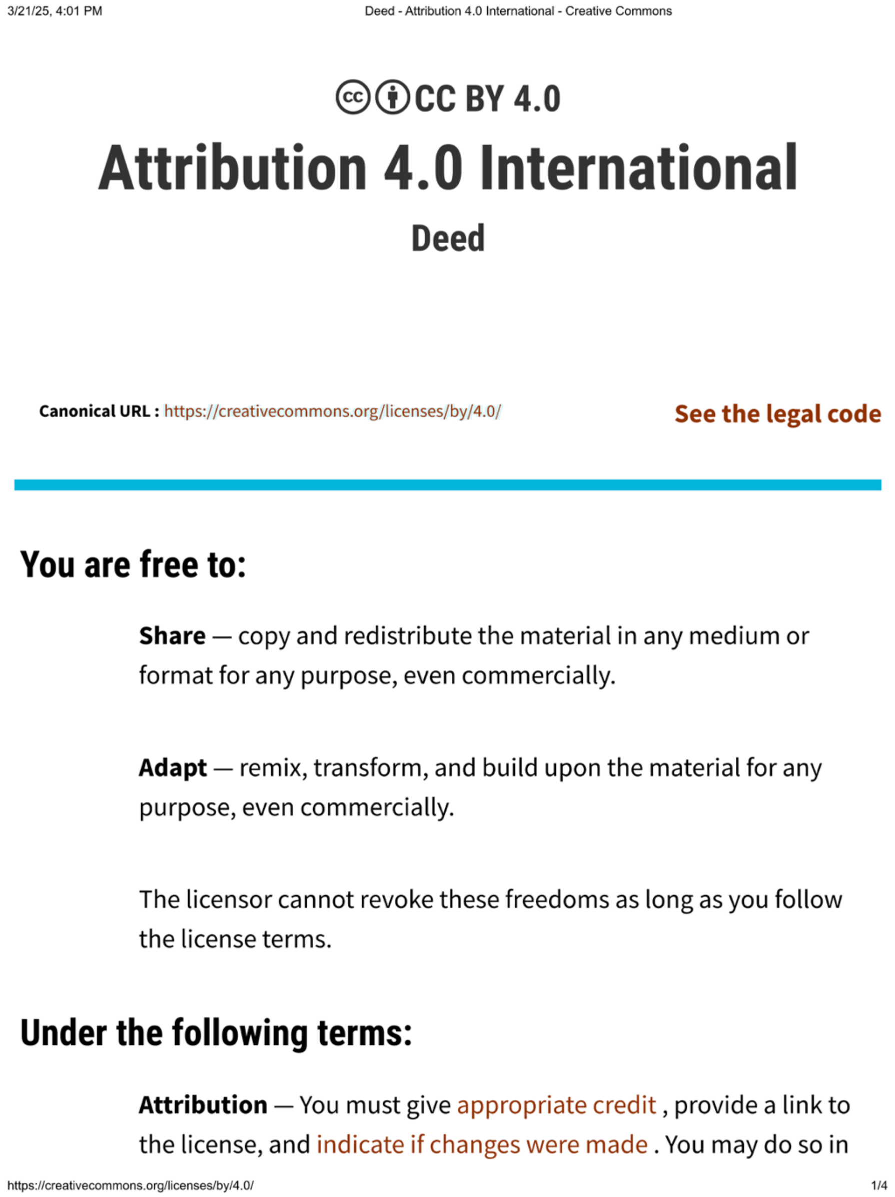

3/21/25, 4:01 PM Deed - Attribution 4.0 International - Creative Commons

CC BY 4.0

Attribution 4.0 International

Deed

Canonical URL : https://creativecommons.org/licenses/by/4.0/

See the legal code

You are free to:

Share — copy and redistribute the material in any medium or format for any purpose, even commercially.

Adapt — remix, transform, and build upon the material for any purpose, even commercially.

The licensor cannot revoke these freedoms as long as you follow the license terms.

Under the following terms:

Attribution — You must give appropriate credit , provide a link to the license, and indicate if changes were made . You may do so in

https://creativecommons.org/licenses/by/4.0/ 1/4

Figure 13 (a) Basic HEMT structure, (b) HEMT with a gate field plate, and (c) cross-section of a double floating field plate HEMT. (d) Electric field distribution in the 2DEG channel at V_d=50V, (e) electric field distribution in the 2DEG channel at breakdown voltage, and (f) comparison of breakdown voltages for the three HEMT structures (P. Wang et al., 2023). Copyright 2023 MDPI.

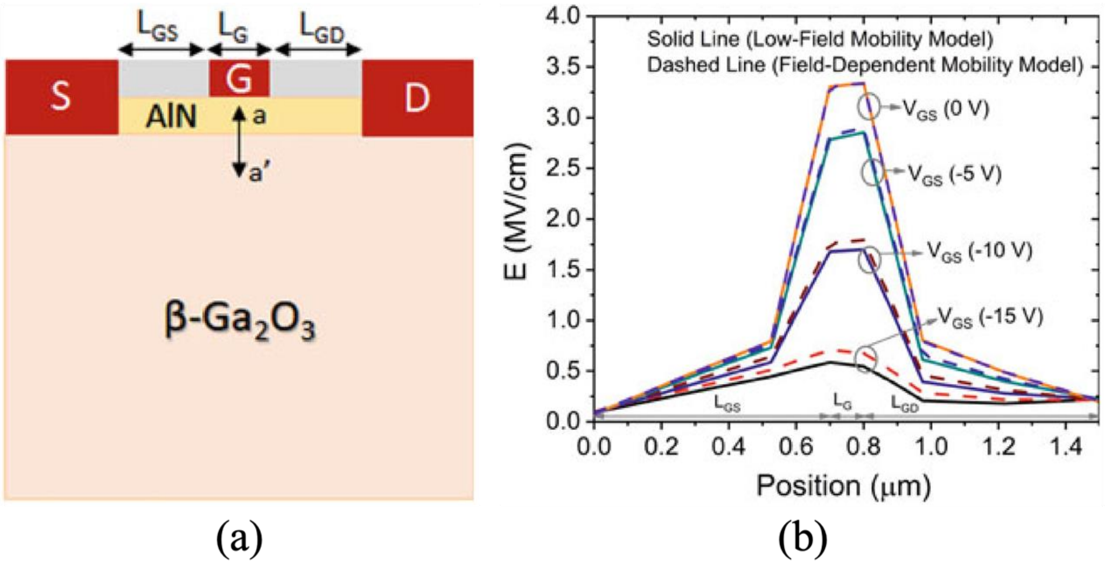

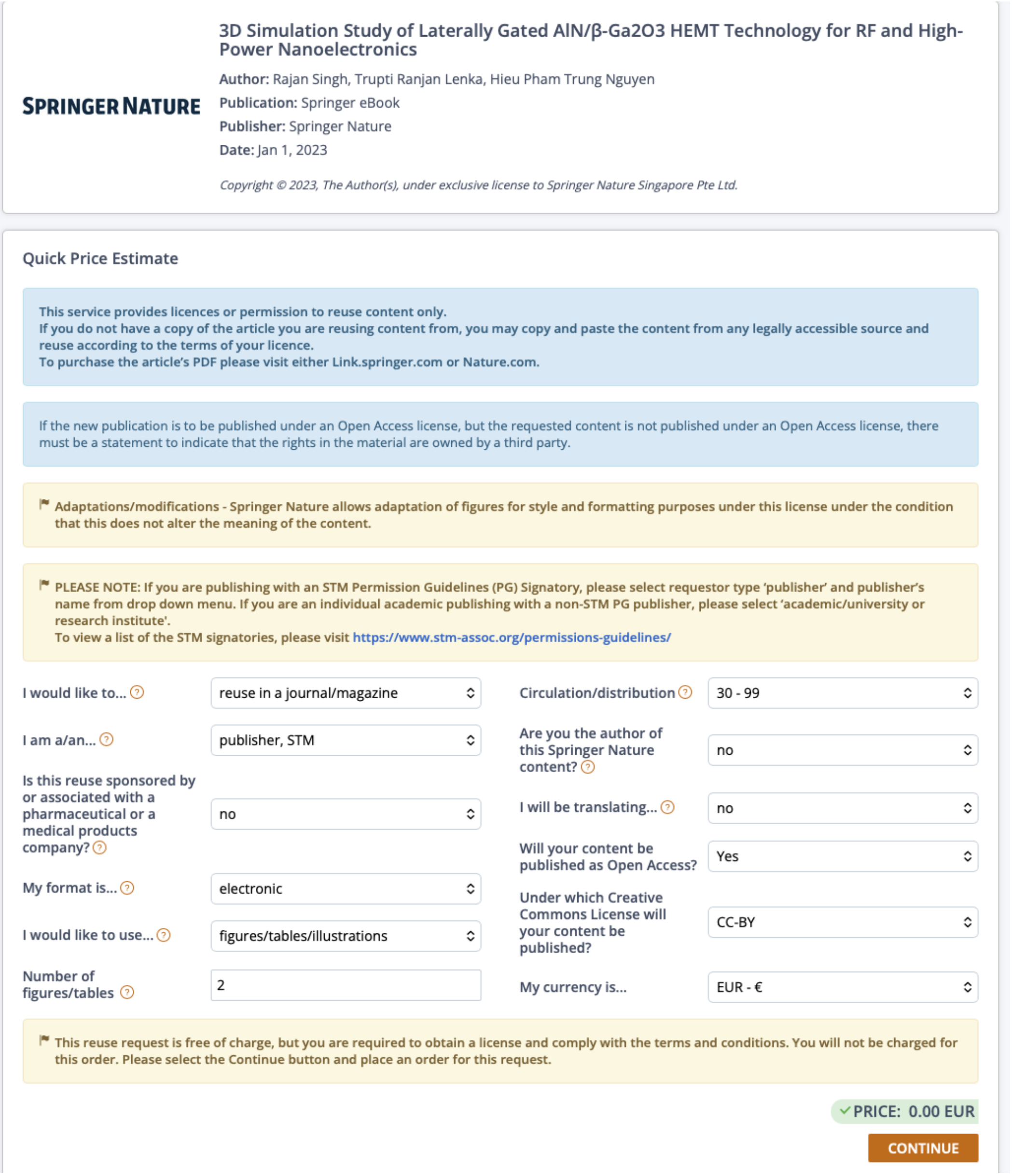
3D Simulation Study of Laterally Gated AlN/β-Ga2O3 HEMT Technology for RF and High-Power Nanoelectronics

Author: Rajan Singh, Trupti Ranjan Lenka, Hieu Pham Trung Nguyen
Publication: Springer eBook
Publisher: Springer Nature
Date: Jan 1, 2023

Copyright © 2023, The Author(s), under exclusive license to Springer Nature Singapore Pte Ltd.

Quick Price Estimate

This service provides licences or permission to reuse content only.
If you do not have a copy of the article you are reusing content from, you may copy and paste the content from any legally accessible source and reuse according to the terms of your licence.
To purchase the article's PDF please visit either Link.springer.com or Nature.com.

If the new publication is to be published under an Open Access license, but the requested content is not published under an Open Access license, there must be a statement to indicate that the rights in the material are owned by a third party.

Adaptations/modifications - Springer Nature allows adaptation of figures for style and formatting purposes under this license under the condition that this does not alter the meaning of the content.

PLEASE NOTE: If you are publishing with an STM Permission Guidelines (PG) Signatory, please select requestor type 'publisher' and publisher's name from drop down menu. If you are an individual academic publishing with a non-STM PG publisher, please select 'academic/university or research institute'.
To view a list of the STM signatories, please visit https://www.stm-assoc.org/permissions-guidelines/

I would like to...	reuse in a journal/magazine	Circulation/distribution	30 - 99
I am a/an...	publisher, STM	Are you the author of this Springer Nature content?	no
Is this reuse sponsored by or associated with a pharmaceutical or a medical products company?	no	I will be translating...	no
		Will your content be published as Open Access?	Yes
My format is...	electronic	Under which Creative Commons License will your content be published?	CC-BY
I would like to use...	figures/tables/illustrations		
Number of figures/tables	2	My currency is...	EUR - €

This reuse request is free of charge, but you are required to obtain a license and comply with the terms and conditions. You will not be charged for this order. Please select the Continue button and place an order for this request.

PRICE: 0.00 EUR

CONTINUE

Figure 14 (a) Cross-sectional structure of AlN/B-Ga_2O_3 HEMT, and (b) electric field distribution under the gate simulated. using the low-field mobility model and the field-dependent mobility model at different V_g with V_d=3V. Reprinted under terms of the CC-BY license (Singh et al., 2023). Copyright 2022, Singh et al., published by Springer Nature.

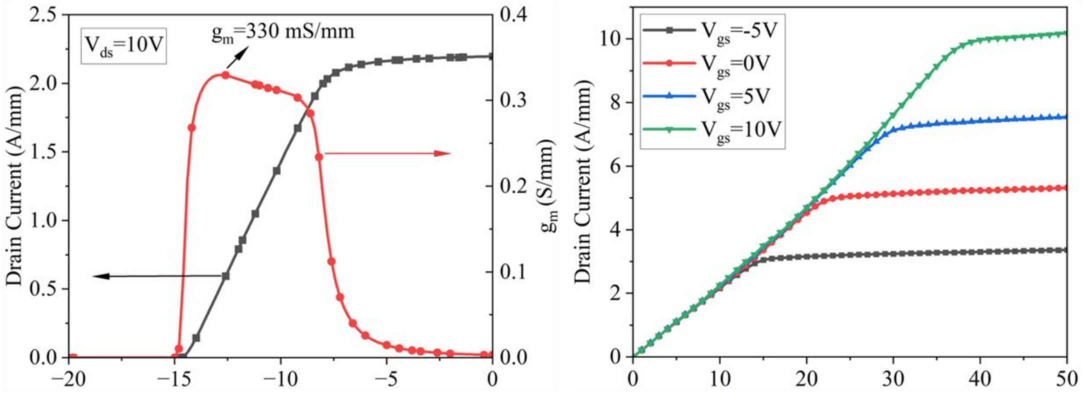

(b) (c)

Rights and permissions

Open Access This article is licensed under a Creative Commons Attribution 4.0 International License, which permits use, sharing, adaptation, distribution and reproduction in any medium or format, as long as you give appropriate credit to the original author(s) and the source, provide a link to the Creative Commons licence, and indicate if changes were made. The images or other third party material in this article are included in the article's Creative Commons licence, unless indicated otherwise in a credit line to the material. If material is not included in the article's Creative Commons licence and your intended use is not permitted by statutory regulation or exceeds the permitted use, you will need to obtain permission directly from the copyright holder. To view a copy of this licence, visit http://creativecommons.org/licenses/by/4.0/.

Figure 15 (a) Cross-sectional structure of the ε−Ga_2O_3/GaN HEMT, (b) transmission and (c) output characteristics. Reprinted under terms of the CC-BY license (Qu et al., 2025). Copyright 2025, Qu et al., published by Springer Nature.

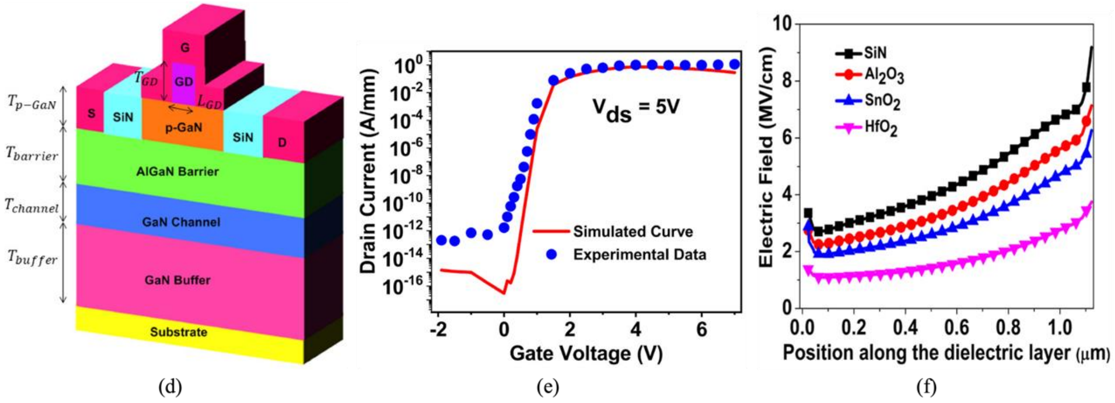

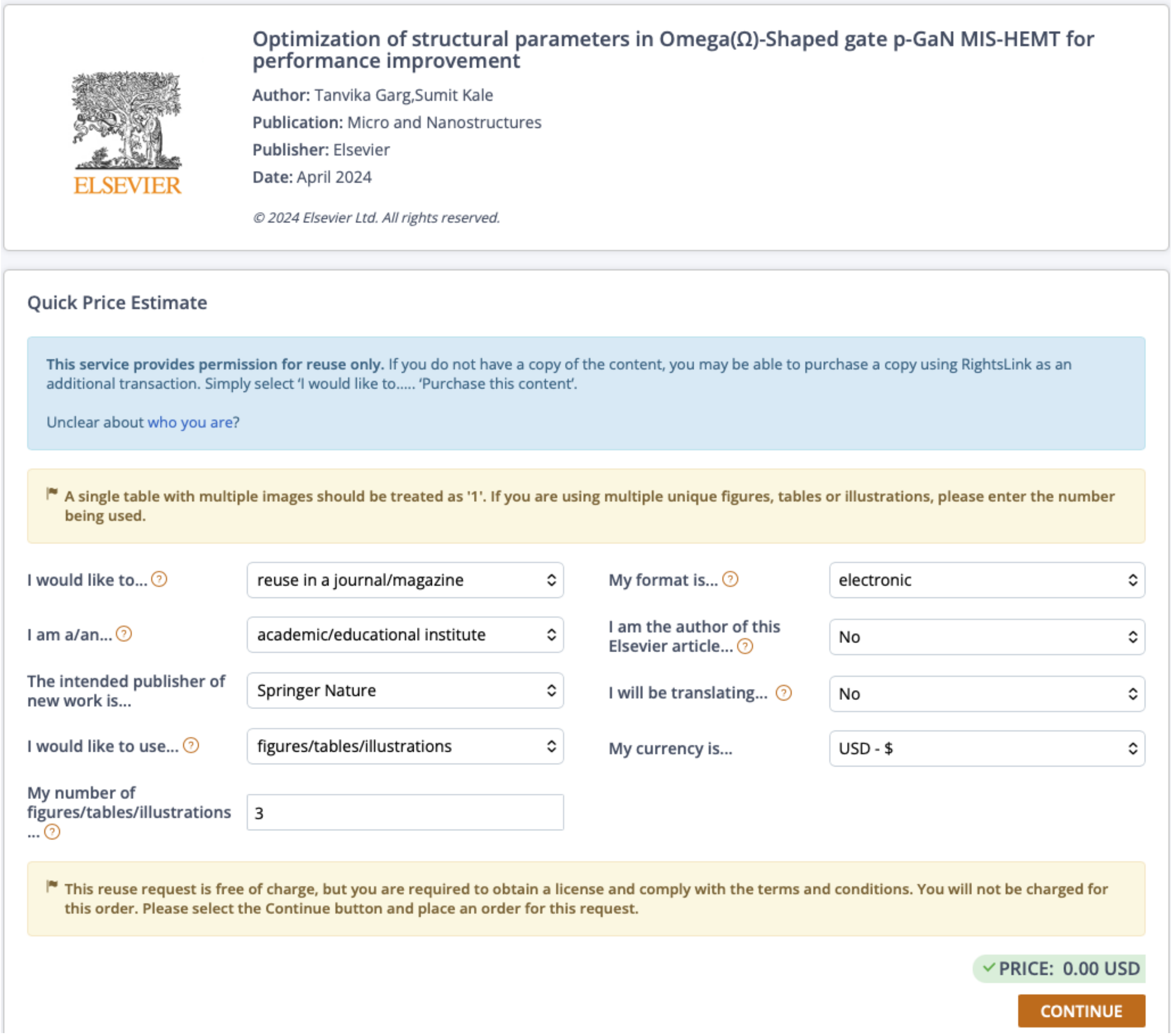

Optimization of structural parameters in Omega(Ω)-Shaped gate p-GaN MIS-HEMT for performance improvement

Author: Tanvika Garg,Sumit Kale

Publication: Micro and Nanostructures

Publisher: Elsevier

Date: April 2024

© 2024 Elsevier Ltd. All rights reserved.

Quick Price Estimate

This service provides permission for reuse only. If you do not have a copy of the content, you may be able to purchase a copy using RightsLink as an additional transaction. Simply select 'I would like to..... 'Purchase this content'.

Unclear about who you are?

A single table with multiple images should be treated as '1'. If you are using multiple unique figures, tables or illustrations, please enter the number being used.

I would like to...	reuse in a journal/magazine
I am a/an...	academic/educational institute
The intended publisher of new work is...	Springer Nature
I would like to use...	figures/tables/illustrations
My number of figures/tables/illustrations ...	3
My format is...	electronic
I am the author of this Elsevier article...	No
I will be translating...	No
My currency is...	USD - $

This reuse request is free of charge, but you are required to obtain a license and comply with the terms and conditions. You will not be charged for this order. Please select the Continue button and place an order for this request.

PRICE: 0.00 USD

CONTINUE

Figure 15 (d) Cross-sectional structure of an omega-gate p-GaN MIS-HEMT, (e) transfer characteristics comparing experimental and simulated results, and (f) impact of different gate dielectric materials on the electric field distribution at breakdown voltage. Reprinted with permission from ref. (Garg & Kale, 2024). Copyright 2024 Elsevier.

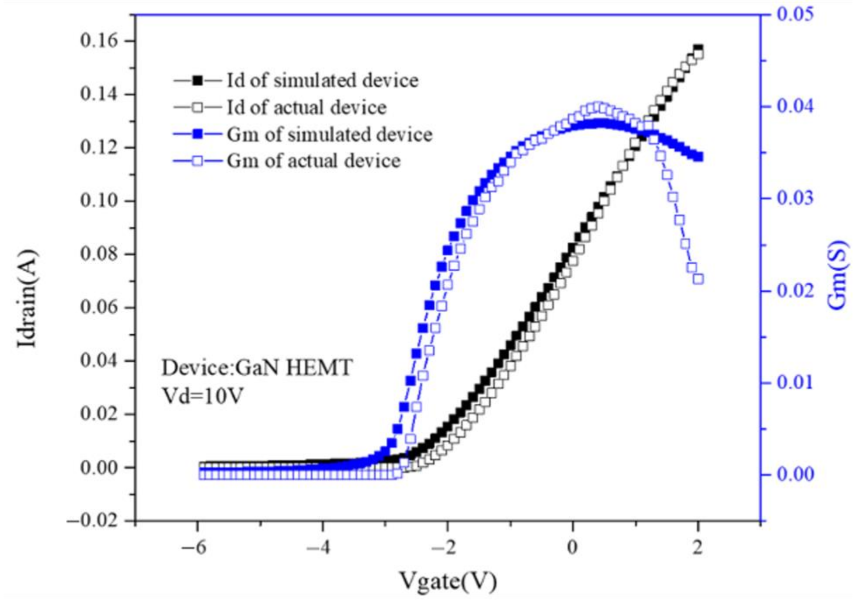

(b)

MDPI Open Access Information and Policy

All articles published by MDPI are made immediately available worldwide under an open access license. This means the following:

- Everyone has free and unlimited access to the full texts of *all* articles published in MDPI journals;
- Everyone is free to reuse the published material if the original article is properly accredited and cited;
- Open access publication is supported by the authors' institutes or research funding agencies via the payment of a comparatively low **Article Processing Charge (APC)** for accepted articles.

Permissions

No special permission is required to reuse all or part of articles published by MDPI, including figures and tables. For articles published under an open access Creative Common CC BY license, any part of the article may be reused without permission provided that it is clearly cited. The reuse of an article does not imply endorsement from the authors or MDPI. Furthermore, no special permission is required for authors to submit their research to external repositories. This policy extends to all versions of a paper, including its submitted, accepted, and published forms.

Figure 16 (b) transfer characteristics and transconductance results comparing simulation and experimental tests. Reprinted with permission from ref. (Z. Wang et al., 2023). Copyright 2023 MDPI.

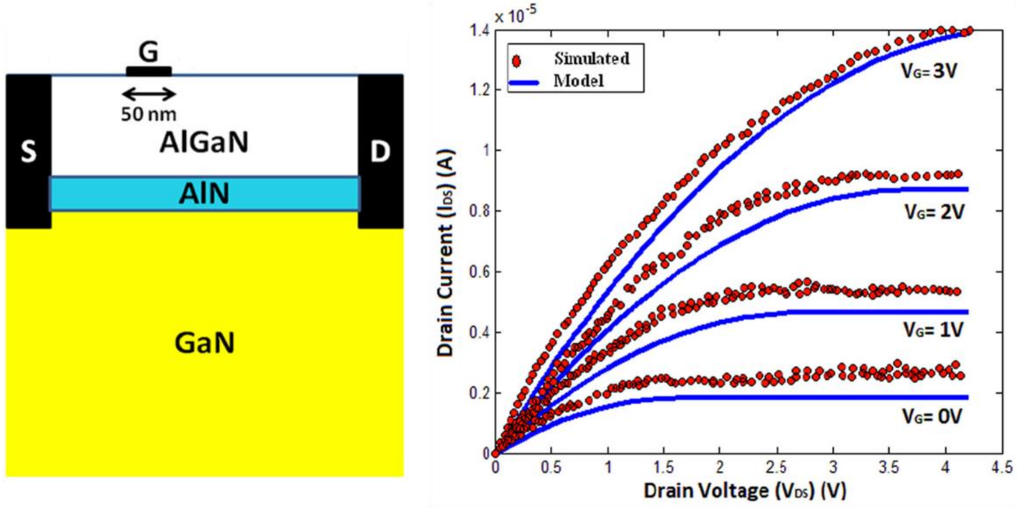

(a) (b)

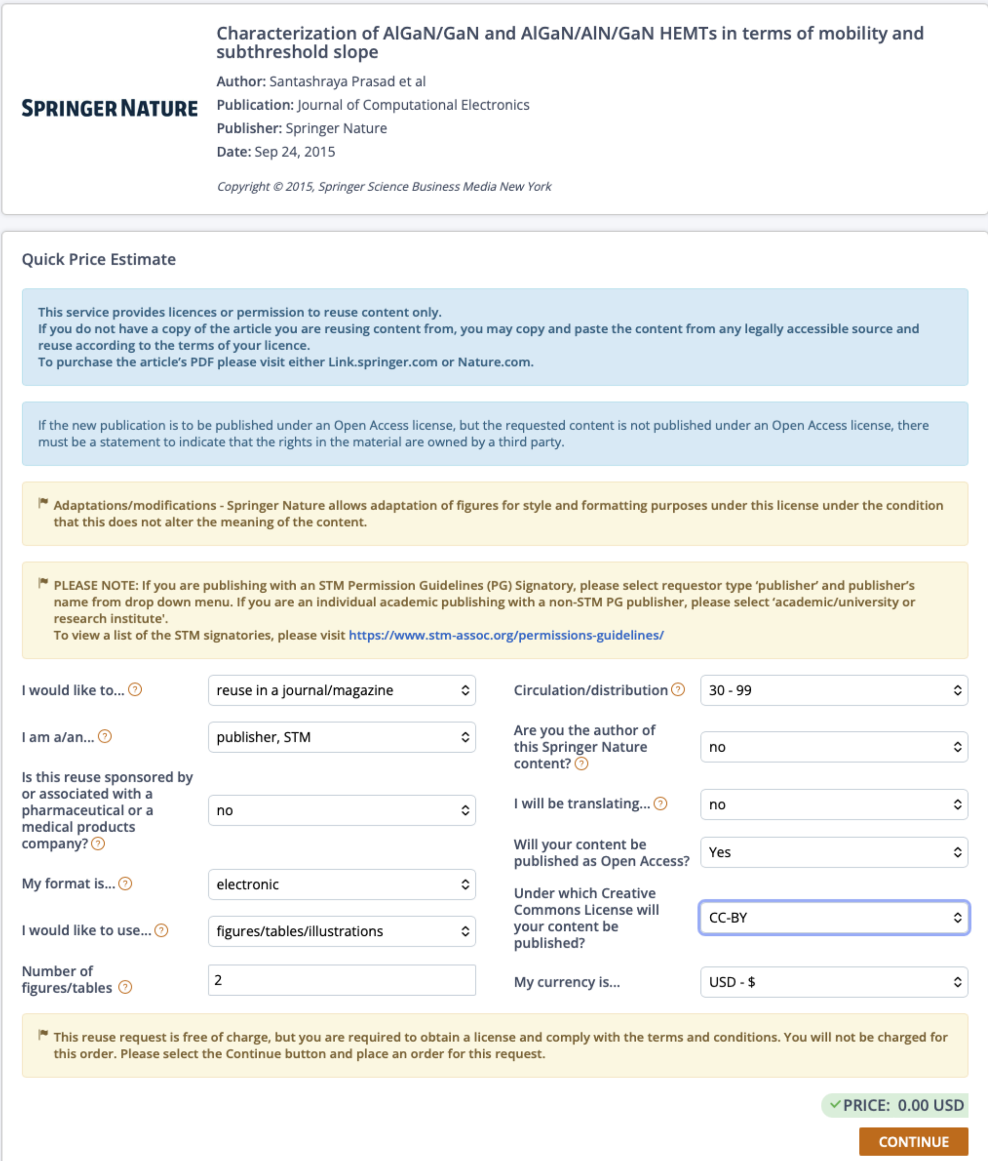

Characterization of AlGaN/GaN and AlGaN/AlN/GaN HEMTs in terms of mobility and subthreshold slope

SPRINGER NATURE

Author: Santashraya Prasad et al

Publication: Journal of Computational Electronics

Publisher: Springer Nature

Date: Sep 24, 2015

Copyright © 2015, Springer Science Business Media New York

Quick Price Estimate

This service provides licences or permission to reuse content only.
If you do not have a copy of the article you are reusing content from, you may copy and paste the content from any legally accessible source and reuse according to the terms of your licence.
To purchase the article's PDF please visit either Link.springer.com or Nature.com.

If the new publication is to be published under an Open Access license, but the requested content is not published under an Open Access license, there must be a statement to indicate that the rights in the material are owned by a third party.

Adaptations/modifications - Springer Nature allows adaptation of figures for style and formatting purposes under this license under the condition that this does not alter the meaning of the content.

PLEASE NOTE: If you are publishing with an STM Permission Guidelines (PG) Signatory, please select requestor type 'publisher' and publisher's name from drop down menu. If you are an individual academic publishing with a non-STM PG publisher, please select 'academic/university or research institute'.
To view a list of the STM signatories, please visit https://www.stm-assoc.org/permissions-guidelines/

I would like to...	reuse in a journal/magazine	Circulation/distribution	30 - 99
I am a/an...	publisher, STM	Are you the author of this Springer Nature content?	no
Is this reuse sponsored by or associated with a pharmaceutical or a medical products company?	no	I will be translating...	no
		Will your content be published as Open Access?	Yes
My format is...	electronic	Under which Creative Commons License will your content be published?	CC-BY
I would like to use...	figures/tables/illustrations		
Number of figures/tables	2	My currency is...	USD - $

This reuse request is free of charge, but you are required to obtain a license and comply with the terms and conditions. You will not be charged for this order. Please select the Continue button and place an order for this request.

PRICE: 0.00 USD

CONTINUE

Figure 17(a) Cross-sectional structure of an AlGaN/AlN/GaN HEMT, (b) output characteristic curves. Copyright 2014, Prasad et al., published by Elsevier.

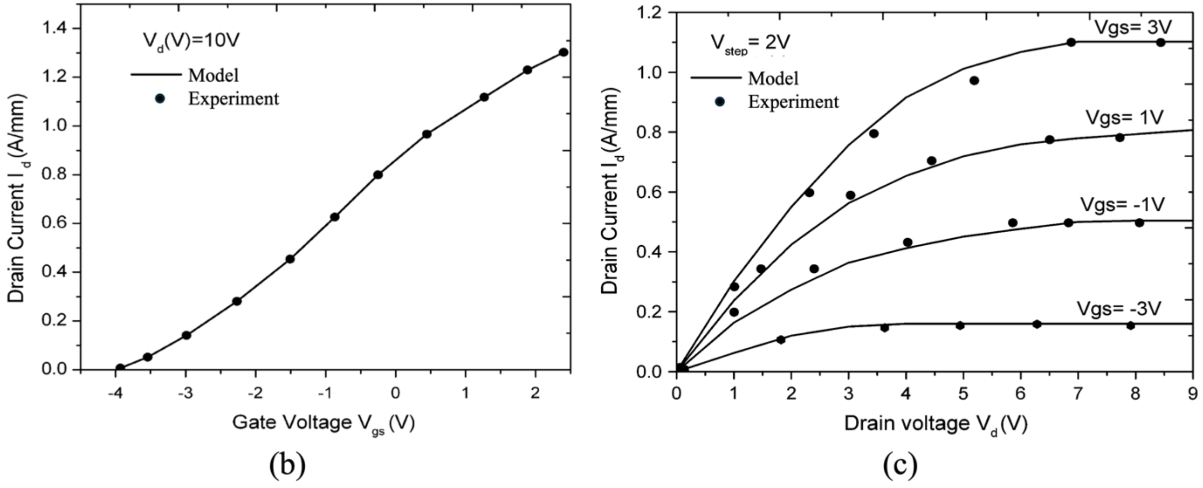

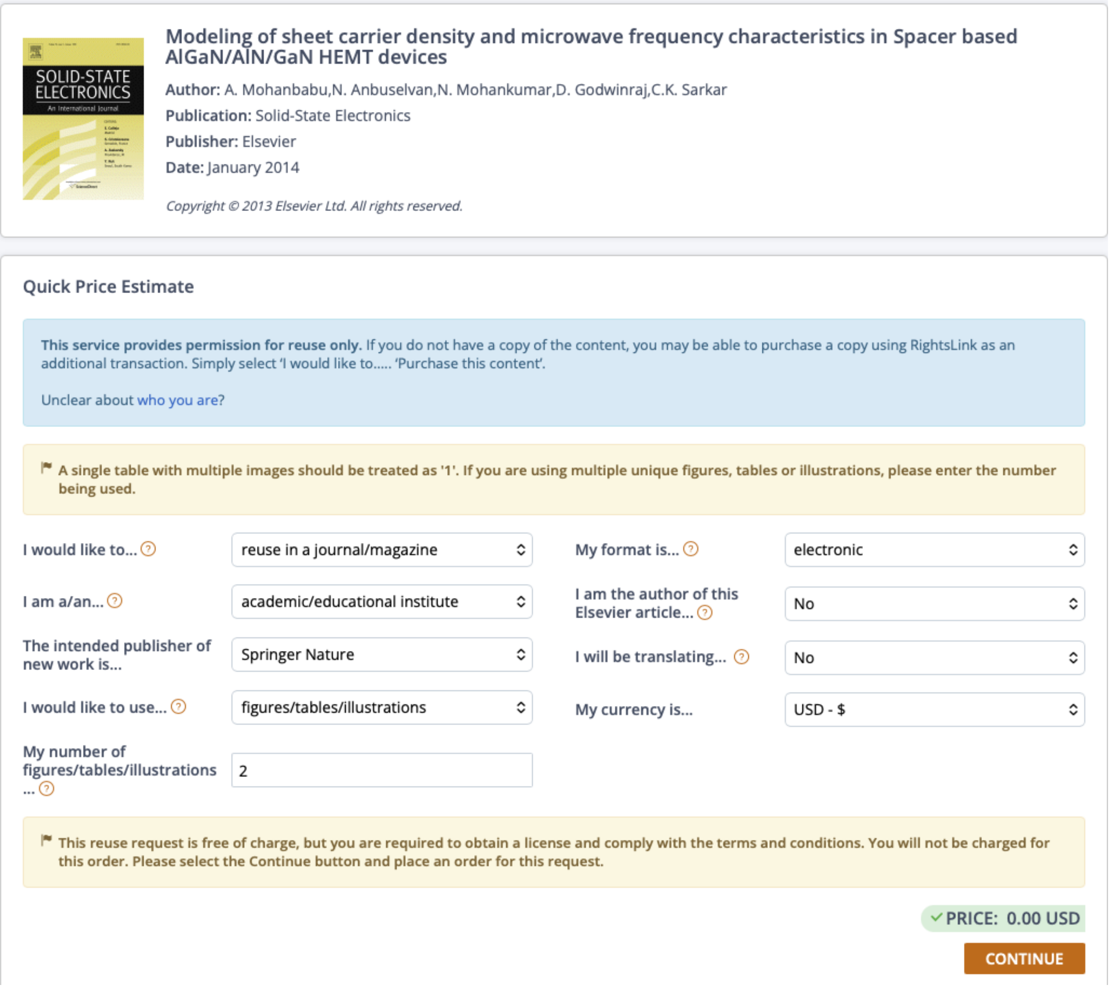

Modeling of sheet carrier density and microwave frequency characteristics in Spacer based AlGaN/AlN/GaN HEMT devices

Author: A. Mohanbabu,N. Anbuselvan,N. Mohankumar,D. Godwinraj,C.K. Sarkar
Publication: Solid-State Electronics
Publisher: Elsevier
Date: January 2014

Copyright © 2013 Elsevier Ltd. All rights reserved.

Quick Price Estimate

This service provides permission for reuse only. If you do not have a copy of the content, you may be able to purchase a copy using RightsLink as an additional transaction. Simply select 'I would like to..... 'Purchase this content'.

Unclear about who you are?

A single table with multiple images should be treated as '1'. If you are using multiple unique figures, tables or illustrations, please enter the number being used.

I would like to...	reuse in a journal/magazine	My format is...	electronic
I am a/an...	academic/educational institute	I am the author of this Elsevier article...	No
The intended publisher of new work is...	Springer Nature	I will be translating...	No
I would like to use...	figures/tables/illustrations	My currency is...	USD - $
My number of figures/tables/illustrations ...	2		

This reuse request is free of charge, but you are required to obtain a license and comply with the terms and conditions. You will not be charged for this order. Please select the Continue button and place an order for this request.

PRICE: 0.00 USD

CONTINUE

Figure 18 (b) comparison of transfer characteristics and (c) output characteristics between simulation results and experimental data (Mohanbabu et al., 2014). Copyright 2014, Mohanbabu et al., published by Elsevier.

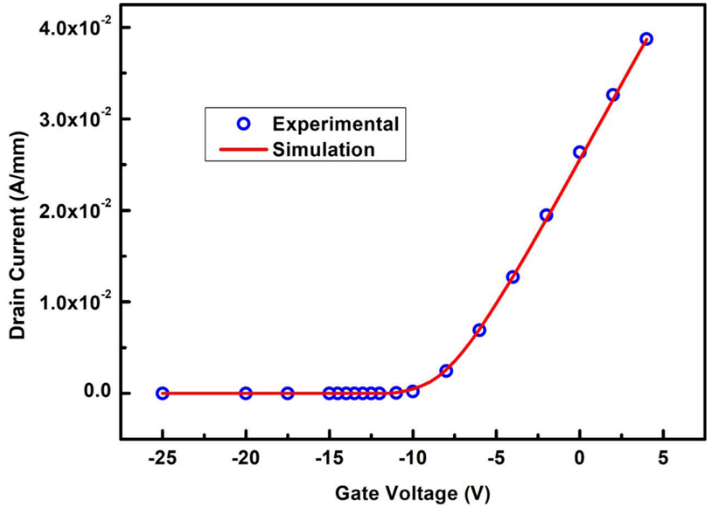

(b)

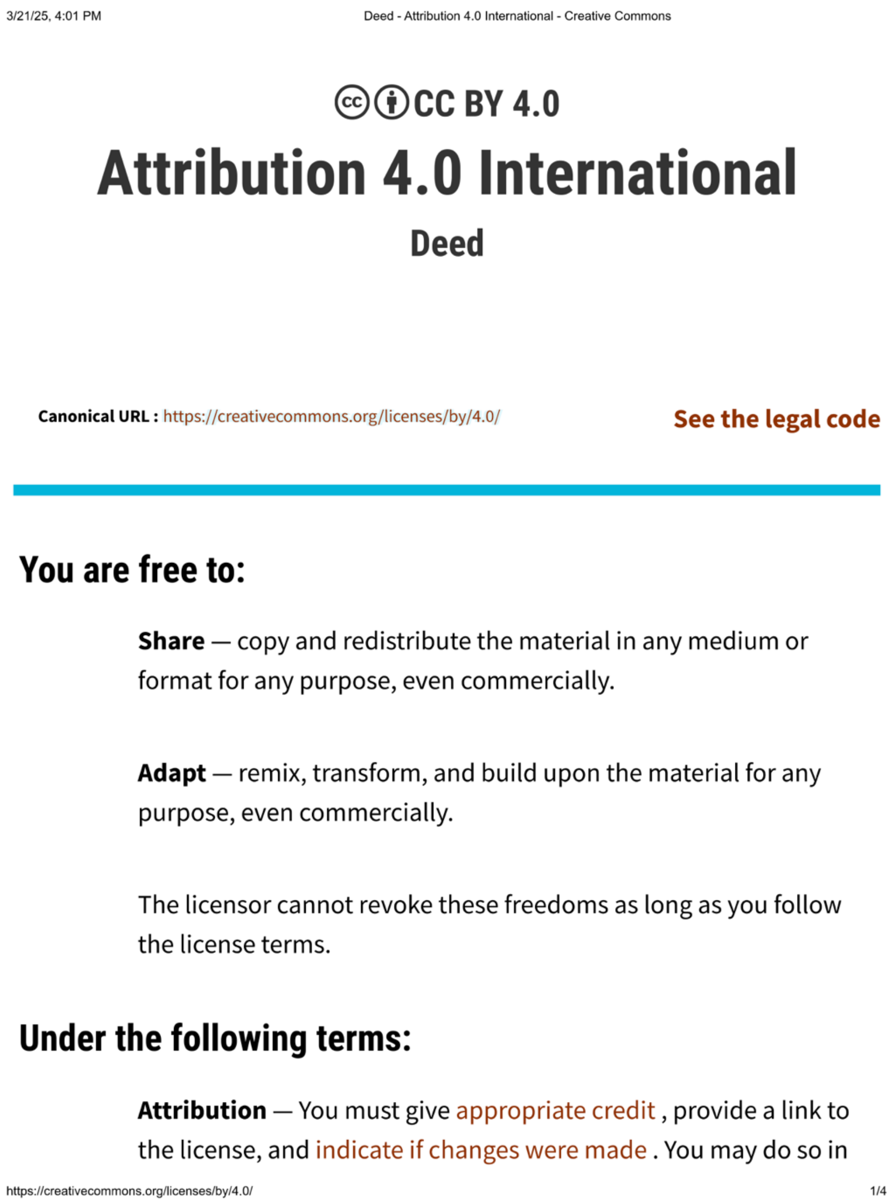
3/21/25, 4:01 PM Deed - Attribution 4.0 International - Creative Commons

CC BY 4.0

Attribution 4.0 International

Deed

Canonical URL : https://creativecommons.org/licenses/by/4.0/

See the legal code

You are free to:

Share — copy and redistribute the material in any medium or format for any purpose, even commercially.

Adapt — remix, transform, and build upon the material for any purpose, even commercially.

The licensor cannot revoke these freedoms as long as you follow the license terms.

Under the following terms:

Attribution — You must give appropriate credit , provide a link to the license, and indicate if changes were made . You may do so in

https://creativecommons.org/licenses/by/4.0/ 1/4

Figure 19 (b) comparison of simulation and experimental results for AlN/β−Ga_2O_3 HEMT. Reprinted with permission (Tomar et al., 2025).

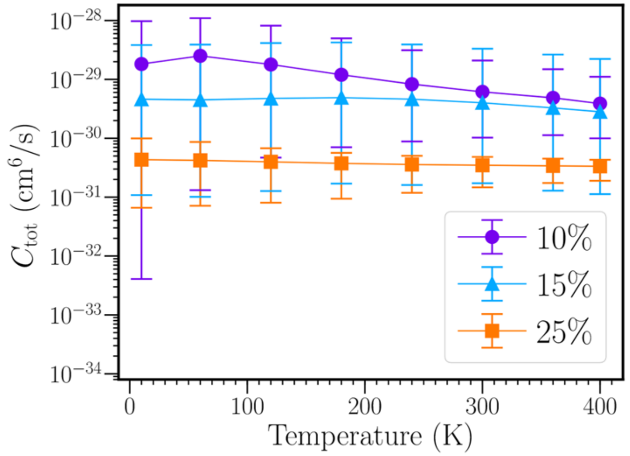

(a)

Figure 20 (a) total Auger coefficient of (In,Ga)N/GaN quantum trapped planes as the functions of temperature (10%, 15%, and 25%). Reproduced with permission from ref. (McMahon et al., 2022). Copyright 2022 American Physical Society.

American Physical Society
Reuse and Permissions License

18-Mar-2026

This license agreement between the American Physical Society ("APS") and Muhd Farizuan Farizuan ("You") consists of your license details and the terms and conditions provided by the American Physical Society and SciPris.

Licensed Content Information

**License Number:**	RNP/26/MAR/103264
**License date:**	18-Mar-2026
**DOI:**	10.1103/PhysRevB.105.195307
**Title:**	Atomistic analysis of Auger recombination in c-plane (In,Ga)N/GaN quantum wells: Temperature-dependent competition between radiative and nonradiative recombination
**Author:**	Joshua M. McMahon, Emmanouil Kioupakis, and Stefan Schulz
**Publication:**	Physical Review B
**Publisher:**	American Physical Society
**Cost:**	USD $ 0.00

Request Details

Does your reuse require significant modifications: No

**Specify intended distribution locations:**	Worldwide
**Reuse Category:**	Reuse in a journal/magazine
**Requestor Type:**	Academic Institution
**Items for Reuse:**	Figures/Tables
**Number of Figure/Tables:**	1
**Figure/Tables Details:**	total Auger coefficient of (In,Ga)N/GaN quantum trapped planes as the functions of temperature (10%, 15%, and 25%)
**Format for Reuse:**	Electronic

Information about New Publication:

**Publisher:**	Springer Nature
**Publication:**	Discover Nano
**Publication Date:**	Jun. 2025
**Article Title:**	Advanced Physical Modeling Approaches for High-Precision TCAD Simulation of GaN HEMT Power Devices: A Review
**Author(s):**	Haocheng Zhao, Amirul Firdaus, Muhammad Farizuan, Weng-Hooi Tan, Hiroshi Kawarada, Shaili Falina, Mohd Syamsul

License Requestor Information

**Name:**	Muhd Farizuan Farizuan
**Affiliation:**	Individual
**Email Id:**	farizuanfariz00@gmail.com
**Country:**	Malaysia

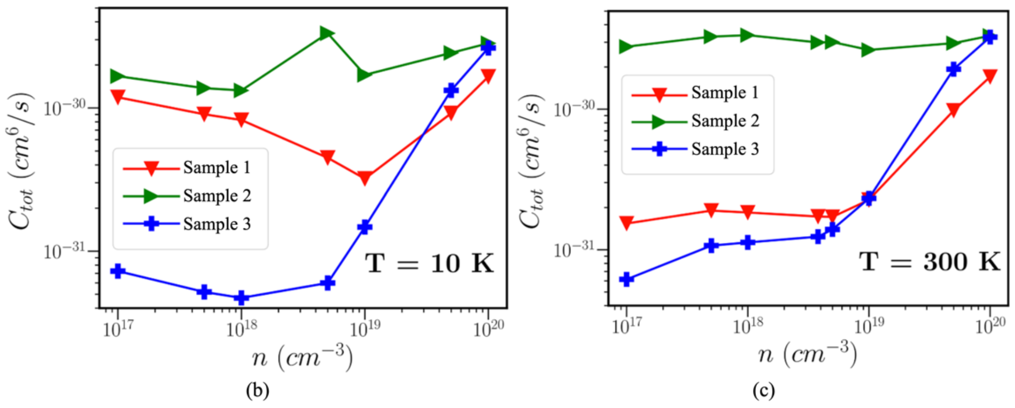

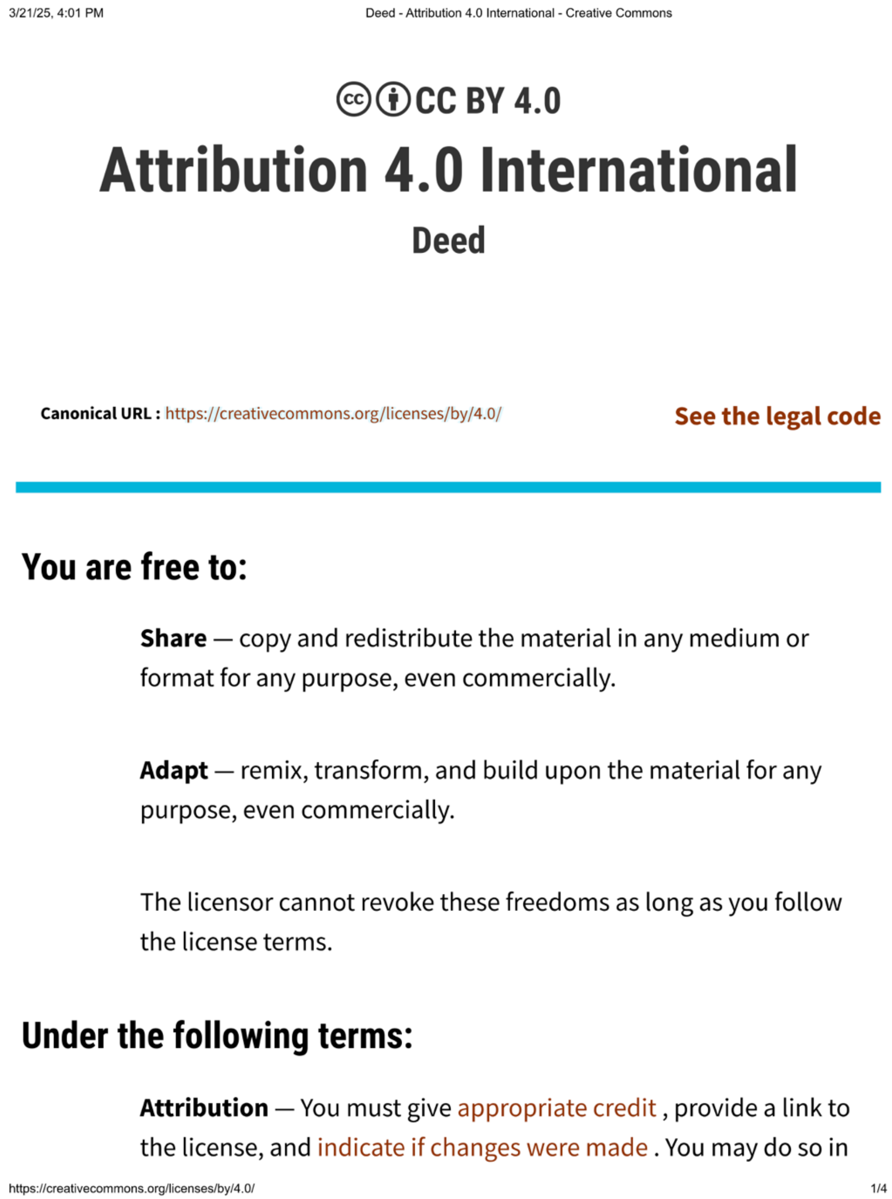

3/21/25, 4:01 PM Deed - Attribution 4.0 International - Creative Commons

CC BY 4.0

Attribution 4.0 International

Deed

Canonical URL : https://creativecommons.org/licenses/by/4.0/

See the legal code

You are free to:

Share — copy and redistribute the material in any medium or format for any purpose, even commercially.

Adapt — remix, transform, and build upon the material for any purpose, even commercially.

The licensor cannot revoke these freedoms as long as you follow the license terms.

Under the following terms:

Attribution — You must give appropriate credit , provide a link to the license, and indicate if changes were made . You may do so in

https://creativecommons.org/licenses/by/4.0/ 1/4

Figure 20 Dependence of the total Auger recombination coefficient on carrier density at (b) T = 10 K and (c) T = 300 K for 3 samples. Reproduce from IOP science under the terms of the Creative Commons Attribution 4.0 license (McMahon et al., 2024).

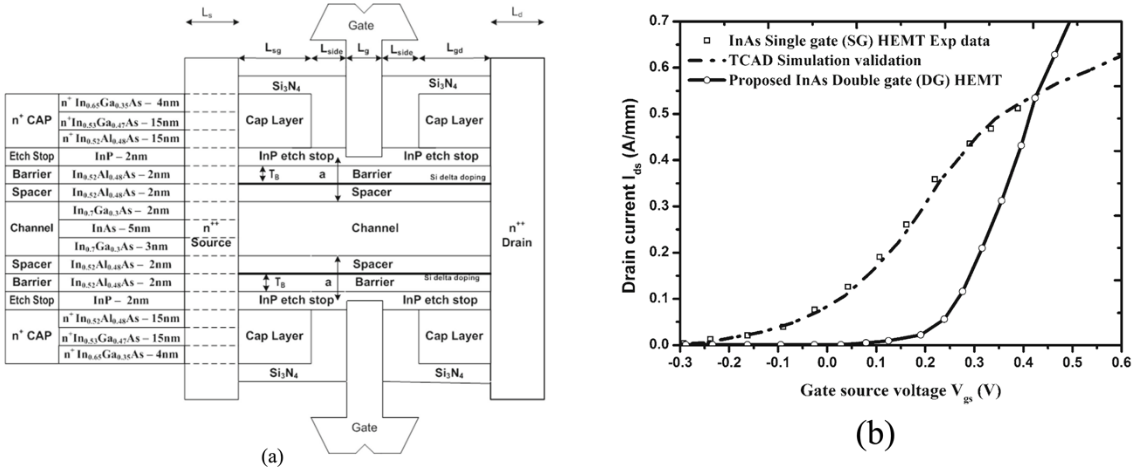

Figure 21 (a) Cross-sectional structure of the InGaAs/InAs/InGaAs composite channel dual-gate HEMT, and (b) transfer characteristics comparing experimental results, simulation verification, and the proposed dual-gate HEMT. Reprinted under terms of the CC-BY license (Saravana Kumar et al., 2017). Copyright 2017, Saravana et al., published by Springer Nature.

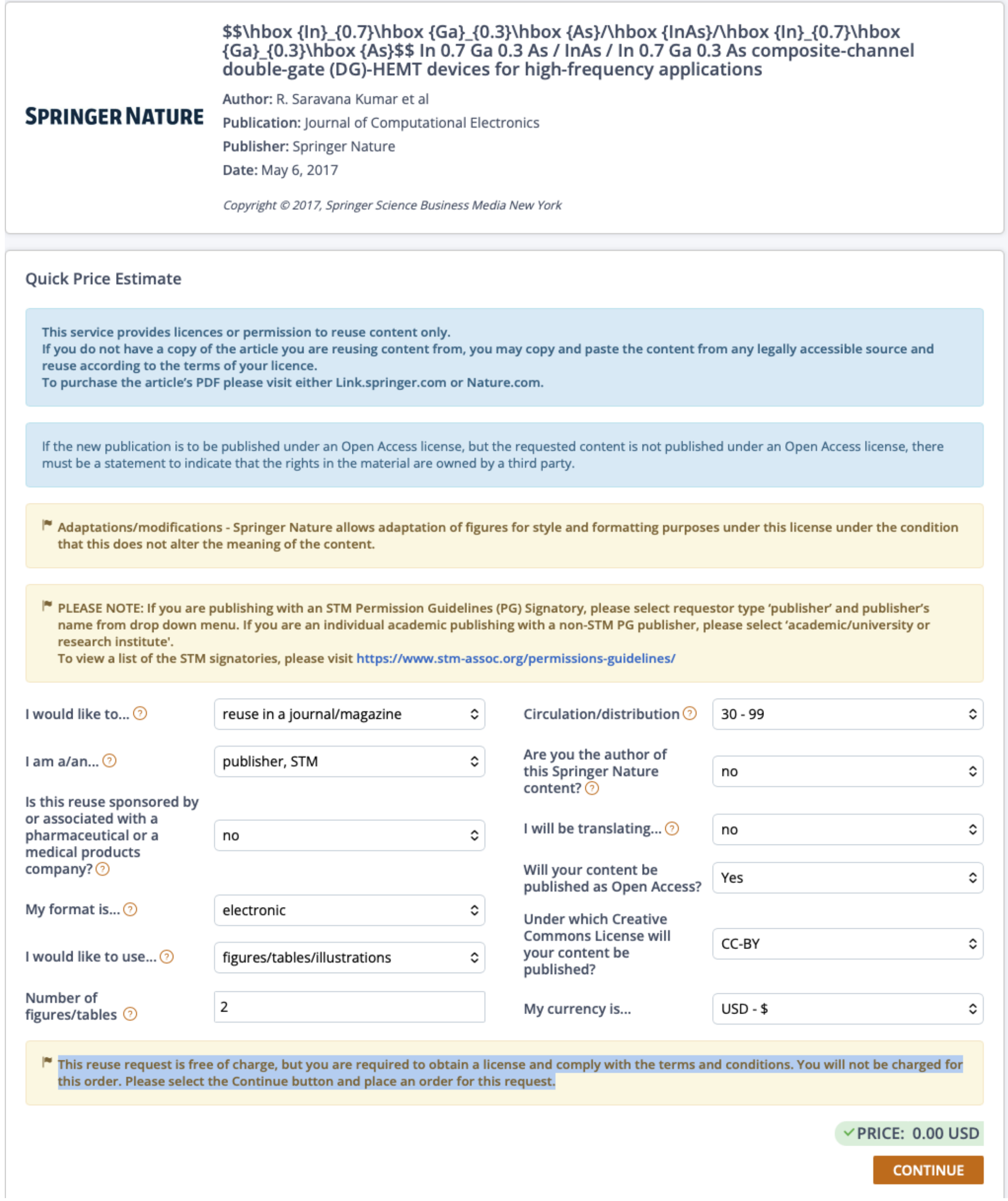

SPRINGER NATURE

$$\hbox {In}_{0.7}\hbox {Ga}_{0.3}\hbox {As}/\hbox {InAs}/\hbox {In}_{0.7}\hbox {Ga}_{0.3}\hbox {As}$$ In 0.7 Ga 0.3 As / InAs / In 0.7 Ga 0.3 As composite-channel double-gate (DG)-HEMT devices for high-frequency applications

Author: R. Saravana Kumar et al
Publication: Journal of Computational Electronics
Publisher: Springer Nature
Date: May 6, 2017

Copyright © 2017, Springer Science Business Media New York

Quick Price Estimate

This service provides licences or permission to reuse content only.
If you do not have a copy of the article you are reusing content from, you may copy and paste the content from any legally accessible source and reuse according to the terms of your licence.
To purchase the article's PDF please visit either Link.springer.com or Nature.com.

If the new publication is to be published under an Open Access license, but the requested content is not published under an Open Access license, there must be a statement to indicate that the rights in the material are owned by a third party.

Adaptations/modifications - Springer Nature allows adaptation of figures for style and formatting purposes under this license under the condition that this does not alter the meaning of the content.

PLEASE NOTE: If you are publishing with an STM Permission Guidelines (PG) Signatory, please select requestor type 'publisher' and publisher's name from drop down menu. If you are an individual academic publishing with a non-STM PG publisher, please select 'academic/university or research institute'.
To view a list of the STM signatories, please visit https://www.stm-assoc.org/permissions-guidelines/

I would like to...	reuse in a journal/magazine	Circulation/distribution	30 - 99
I am a/an...	publisher, STM	Are you the author of this Springer Nature content?	no
Is this reuse sponsored by or associated with a pharmaceutical or a medical products company?	no	I will be translating...	no
		Will your content be published as Open Access?	Yes
My format is...	electronic	Under which Creative Commons License will your content be published?	CC-BY
I would like to use...	figures/tables/illustrations		
Number of figures/tables	2	My currency is...	USD - $

This reuse request is free of charge, but you are required to obtain a license and comply with the terms and conditions. You will not be charged for this order. Please select the Continue button and place an order for this request.

PRICE: 0.00 USD

CONTINUE

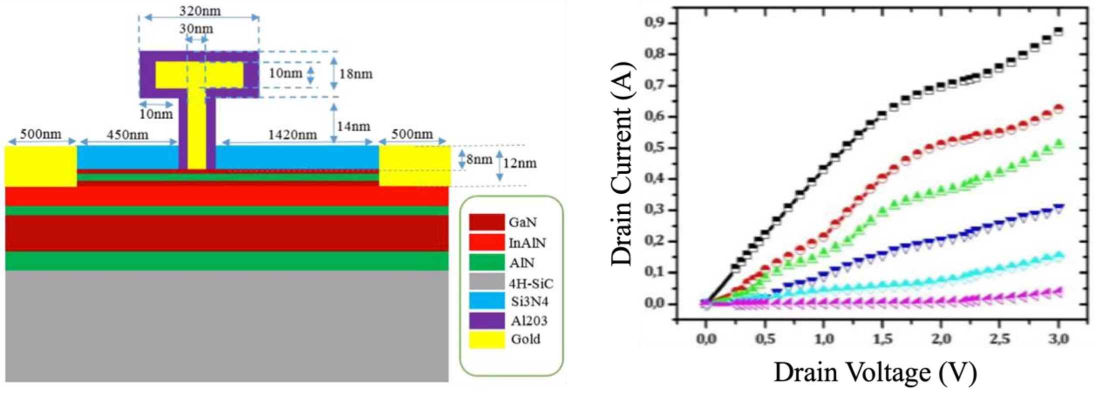

(a) (b)

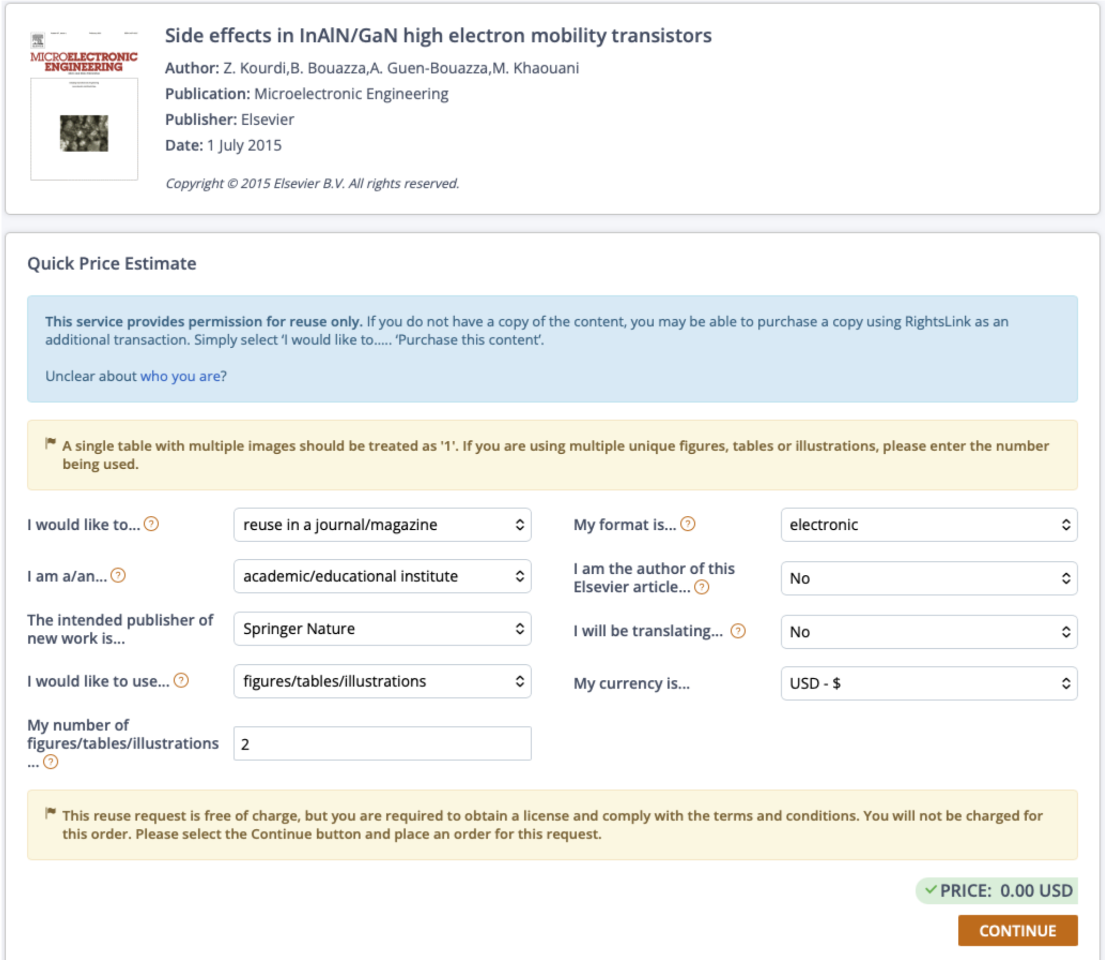

Side effects in InAlN/GaN high electron mobility transistors

Author: Z. Kourdi,B. Bouazza,A. Guen-Bouazza,M. Khaouani

Publication: Microelectronic Engineering

Publisher: Elsevier

Date: 1 July 2015

Copyright © 2015 Elsevier B.V. All rights reserved.

Quick Price Estimate

This service provides permission for reuse only. If you do not have a copy of the content, you may be able to purchase a copy using RightsLink as an additional transaction. Simply select 'I would like to..... 'Purchase this content'.

Unclear about who you are?

A single table with multiple images should be treated as '1'. If you are using multiple unique figures, tables or illustrations, please enter the number being used.

I would like to...	reuse in a journal/magazine	My format is...	electronic
I am a/an...	academic/educational institute	I am the author of this Elsevier article...	No
The intended publisher of new work is...	Springer Nature	I will be translating...	No
I would like to use...	figures/tables/illustrations	My currency is...	USD - $
My number of figures/tables/illustrations ...	2		

This reuse request is free of charge, but you are required to obtain a license and comply with the terms and conditions. You will not be charged for this order. Please select the Continue button and place an order for this request.

PRICE: 0.00 USD

CONTINUE

Figure 22 (a) Cross-sectional structure of the InAlN/GaN HEMT. (b) Simulated output characteristics showing the kink effect. Reprinted with permission from ref. (Kourdi et al., 2015). Copyright 2015 Elsevier.

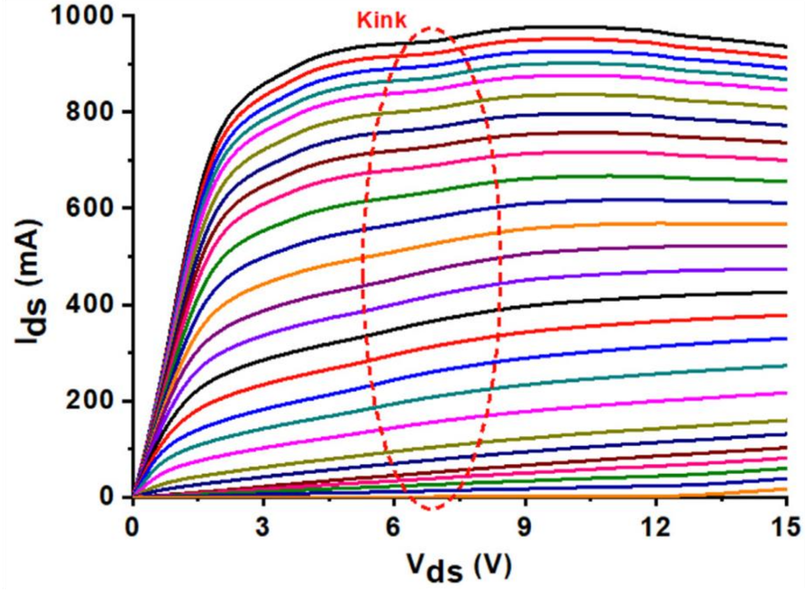

(c)

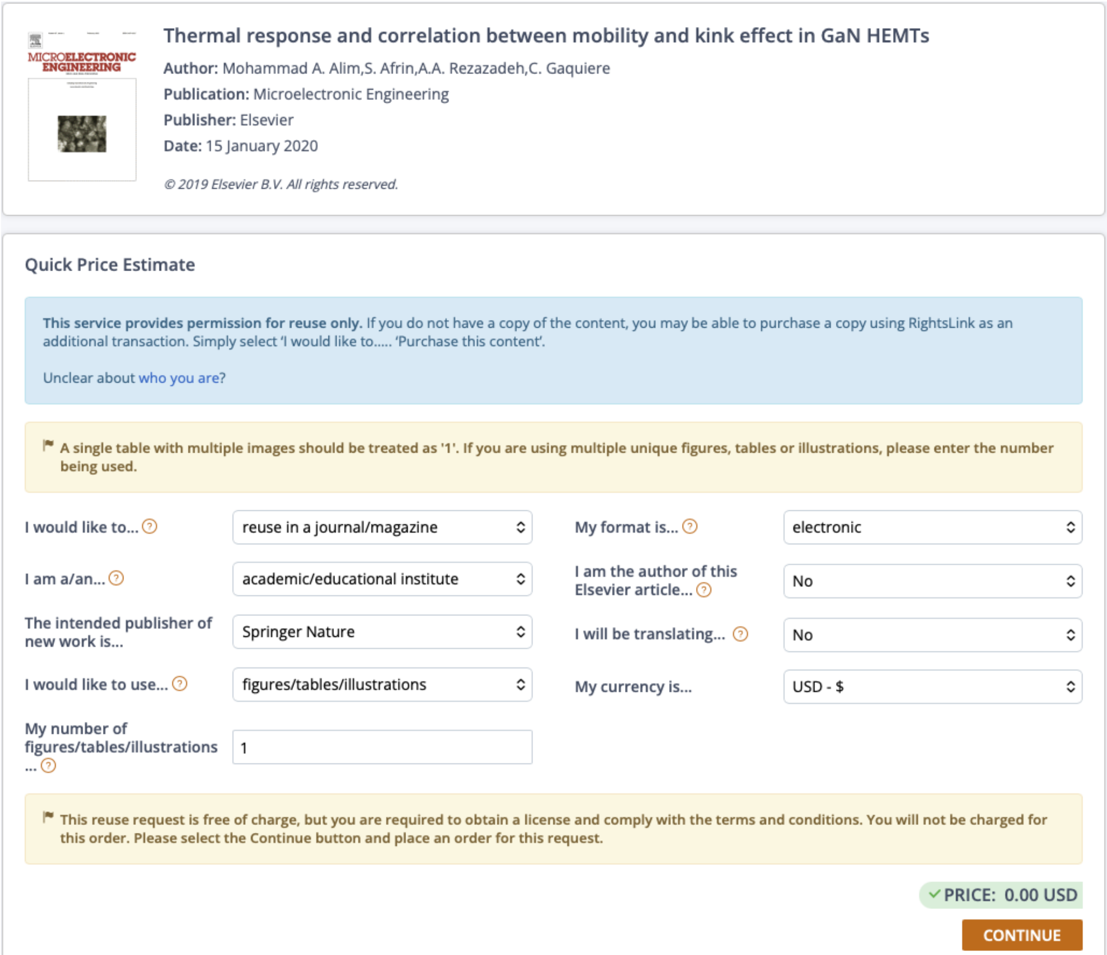
Thermal response and correlation between mobility and kink effect in GaN HEMTs

Author: Mohammad A. Alim,S. Afrin,A.A. Rezazadeh,C. Gaquiere

Publication: Microelectronic Engineering

Publisher: Elsevier

Date: 15 January 2020

© 2019 Elsevier B.V. All rights reserved.

Quick Price Estimate

This service provides permission for reuse only. If you do not have a copy of the content, you may be able to purchase a copy using RightsLink as an additional transaction. Simply select 'I would like to..... 'Purchase this content'.

Unclear about who you are?

A single table with multiple images should be treated as '1'. If you are using multiple unique figures, tables or illustrations, please enter the number being used.

I would like to...	reuse in a journal/magazine
I am a/an...	academic/educational institute
The intended publisher of new work is...	Springer Nature
I would like to use...	figures/tables/illustrations
My number of figures/tables/illustrations ...	1
My format is...	electronic
I am the author of this Elsevier article...	No
I will be translating...	No
My currency is...	USD - $

This reuse request is free of charge, but you are required to obtain a license and comply with the terms and conditions. You will not be charged for this order. Please select the Continue button and place an order for this request.

PRICE: 0.00 USD

CONTINUE

Figure 22 (c) Experimentally observed kink effect in AlGaN/GaN/SiC HEMT. Reprinted with permission from ref. (Alim et al., 2020). Copyright 2020 Elsevier.

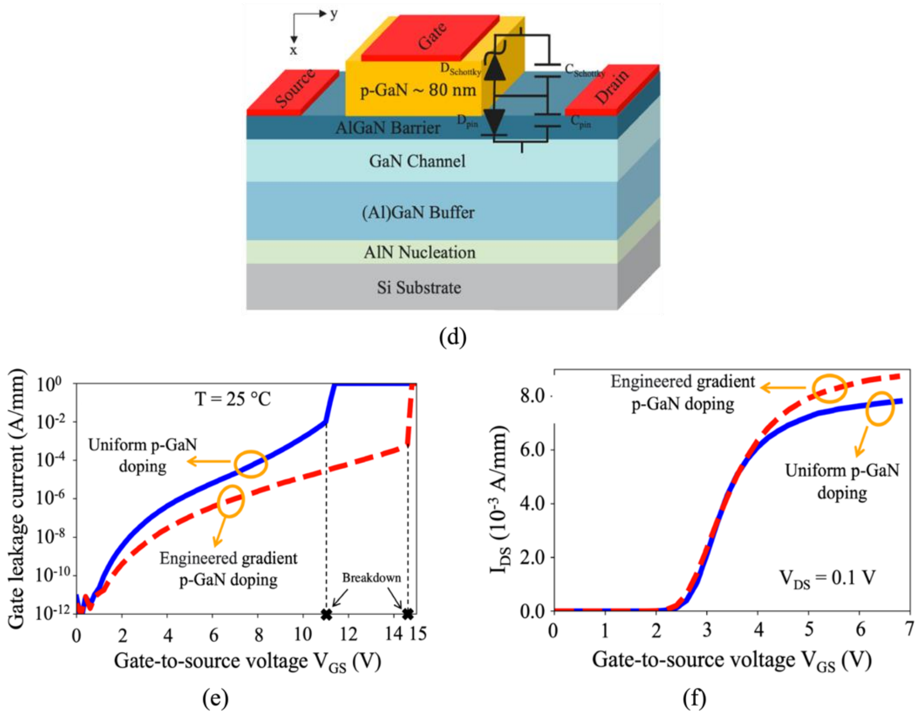

Figure 22 (d) Schottky gate HEMT structure with uniform p-GaN doping. (e) Gate leakage current comparison between uniform p-GaN doped HEMT and engineered gradient p-GaN doped HEMT. (f) Drain current characteristics of uniform and engineered gradient p-GaN doped HEMTs. Reprinted with permission (Alaei et al., 2024). Under the terms of the Creative Commons Attribution 4.0 license.

CC BY 4.0

Attribution 4.0 International

Deed

Canonical URL : https://creativecommons.org/licenses/by/4.0/

See the legal code

You are free to:

Share — copy and redistribute the material in any medium or format for any purpose, even commercially.

Adapt — remix, transform, and build upon the material for any purpose, even commercially.

The licensor cannot revoke these freedoms as long as you follow the license terms.

Under the following terms:

Attribution — You must give appropriate credit , provide a link to the license, and indicate if changes were made . You may do so in
